# CDIN1-Codanin-1 complex defective in congenital dyserythropoietic anaemia type I is an RNA nuclease

**DOI:** 10.1038/s41467-026-74766-7

**Published:** 2026-07-02

**Authors:** Sanja Brolih, Hafiz Saqib Ali, Caroline Scott, Aude-Anais Olijnik, Hazel Aitkenhead, Gemma Moir-Meyer, Angeline E. Gavard, Yuliana Yosaatmadja, Douglas R. Higgs, Veronica Buckle, Noemi Roy, Opher Gileadi, Joseph A. Newman, Fernanda Duarte, Christian Babbs, Peter J. McHugh

**Affiliations:** 1 Department of Oncology, MRC Weatherall Institute of Molecular Medicine, University of Oxford, John Radcliffe Hospital, Oxford, UK; 2 Centre for Medicines Discovery, University of Oxford, Oxford, UK; 3 Chemistry Research Laboratory, University of Oxford, Mansfield Road, Oxford, UK; 4 The Ineos Oxford Institute for Antimicrobial Research, University of Oxford, Oxford, UK; 5 MRC Weatherall Institute of Molecular Medicine, University of Oxford, John Radcliffe Hospital, Oxford, UK; 6 Department of Haematology, Oxford University Hospitals NHS Trust, Churchill Hospital, Old Road, Headington, Oxford, UK; 7 Present Address: Diamond Light Source Ltd., Harwell Science and Innovation Campus, Didcot, UK; 8 Present Address: School of Medical Sciences, Faculty of Medical Health and Sciences, University of Auckland, Auckland, New Zealand; 9 Present Address: SGC Karolinska, Centre for Molecular Medicine, Stockholm, Sweden

**Keywords:** Hydrolases, Molecular medicine

## Abstract

Congenital Dyserythropoietic Anaemia type I (CDA-I) is a rare inherited disorder of erythropoiesis, in which erythroid cells display a unique nuclear phenotype referred to as ‘spongy’ heterochromatin. The molecular basis of CDA-I remains unknown, with most cases of CDA-I caused by mutations in *CDAN1*, encoding Codanin-1, or *CDIN1*, encoding for Codanin-1-interacting nuclease 1 (CDIN1). To date, very little is known about the function of CDA-I disease proteins and the mechanism by which their associated mutations cause disease. Here, we demonstrate that endogenous CDIN1 interacts with Codanin-1, to form a stable complex. Structural and functional analysis of this complex reveals that the CDIN1-Codanin-1 complex is an RNA nuclease. We shed light on the key mechanistic features of the complex using biochemical and biophysical approaches, complemented by all-atom molecular dynamics (MD) structural simulations. We identify various functional consequences of founder patient mutations on the RNA nuclease activity of CDIN1, providing a framework for understanding the pathophysiology and developing therapeutic strategies for CDA-I.

## Abstract (web summary)

Congenital dyserythropoietic anaemia type I is caused by mutations in CDAN1 or CDIN1, but the molecular basis of disease is unclear. Here, the authors show that Codanin-1 and CDIN1 form an RNA nuclease complex and define how disease mutations disrupt its activity.

## Introduction

Congenital Dyserythropoietic Anaemia type I (CDA-I) is a rare recessive autosomal disorder in which anaemia arises because of ineffective erythropoiesis^1^. Light microscopy studies of CDA-I erythroblasts show maturation defects, binuclearity, and internuclear bridging while transmission electron microscopy (TEM) reveals a diagnostic pattern of heterochromatin in ~60% of erythroblasts, unique to CDA-I, termed ‘spongy’ or ‘Swiss cheese’ heterochromatin^2^. On a genetic level, ~90% of CDA-I cases can be accounted for by biallelic mutations in either of the two known causative genes, *CDAN1*, encoding Codanin-1, or *CDIN1*, coding for Codanin-1-interacting nuclease 1 (CDIN1)^3^. Although CDA-I is a disease that specifically affects the erythroid lineage, current insights into CDIN1 and Codanin-1 regulation and function are almost exclusively derived from studies performed in non-erythroid cells. Nonetheless, several reports from model cellular systems suggest that the CDIN1 and Condanin-1 proteins interact, consistent with their dual involvement in CDA-I pathogenesis^4–6^. Cellular studies to date imply that CDIN1 and Codanin-1 are widely expressed and complete loss of *CDAN1* is incompatible with life, underlining the importance of studying these proteins^7–9^.

Recent reports have shed light on Codanin-1 structure and function. Two cryogenic electron microscopy (Cryo-EM) studies demonstrated that (likely homodimeric) Codanin-1 tightly binds the ASF1A/ASF1B histone chaperone proteins via a two-fold interaction with highly conserved histone H3 mimic helix (HMH) and a B-domain^10,11^. The binding of ASF1 with Codanin-1 is competitive with histones H3 and H4, implying that histone supply might be controlled via sequestration of ASF1 isoforms by Codanin-1^4,10,11^, potentially within the cytoplasm, although nucleolar localisation has also been reported for this complex^12,13^. However, Codanin-1 was also able to interact with a preformed ASF1-H3-H4 complex. Therefore, the contribution of Codanin-1 to the regulation of histone deposition might be regulated by controlling handover of this histone-containing complex to deposition chaperones, including CAF-1 and HIRA^14–16^. The structural homology of the Codanin-1 with the CNOT1 protein, a scaffold which coordinates the activities of multiple RNA processing factors (as part of the CCR4-NOT complex), further supports the role for Codanin-1 as a scaffold for its binding partners ASF1A/ASF1B and CDIN1^4,5,17^.

CDIN1 is a largely uncharacterised ~34 kDa protein, predicted to be a member of the PD(D/E)XK superfamily of nucleases; however, no nuclease activity has been reported to date^18^. CDIN1 was not visible in the cryo-EM studies of the Codanin-1-ASF complexes cited above, despite being present. This is likely due to the intrinsic flexibility of the C-terminal (CDIN1-interacting portion) of Codanin-1 or linker region linking the C-terminal CDIN1-Codanin-1 complex to the Codanin-1-ASF1 complexes. Therefore, no experimental structural information is available for this factor, and CDIN1 characterisation remains poor.

Here, we have utilised genome engineering of HUDEP-2 (Human Umbilical Cord Derived Erythroid Progenitor-2) cells^19^ to develop an erythroid model of CDA-I. These cells were derived from CD34^+^ cord blood progenitors^19^ and recapitulate primary cell morphology and immunophenotype as they differentiate^20^. They can be maintained indefinitely as proerythroblasts and form late-stage orthochromatic erythroblasts with efficient chromatin condensation and haemoglobin (adult) accumulation^21^. Use of this erythroid model showed that endogenous CDIN1-Codanin-1 interact in a relevant physiological context. We then developed a strategy to purify the CDIN1-Codanin-1 complex, and identified CDIN1 as an RNA exonuclease, requiring a free 3′-terminus and displaying a preference for dsRNA-containing substrates. We find that the association between CDIN1 and Codanin-1 modulates the activity of the nuclease. This CDIN1-Codanin-1 complex is a versatile nuclease complex, able to digest a diverse range of RNA molecules, in both ssRNA and dsRNA. We also show the detrimental effects of CDIN1 Y94C and L178Q CDA-I patient mutations on nuclease activity, providing a possible explanation for the disease phenotype.

To gain an atomic-level understanding of the CDA-I disease mechanism, we investigated the structure, dynamics, and interactions of biomolecular complexes using a combination of AlphaFold (AF) and all-atom molecular dynamics (MD) simulations^22–24^. The simulations, validated by mutagenesis experiments, reveal the structural basis of the interaction between CDIN1 and Codanin-1, providing key insights into how formation of this complex modulates RNA nuclease activity. Additionally, we also explored how CDA-I-associated disease mutations (Y94C and L178Q) might alter conformational stability and functions of CDIN1 without the use of scarcely-available patient material.

Together, this study identifies the CDIN1-Codanin-1 complex as an RNA nuclease and details key molecular insights into the underlying defect of CDA-I, offering the foundation for future therapeutic strategies.

## Results

### Endogenous CDIN1 and Codanin-1 form a complex in vivo

The differentiation of human CD34^+^ human stem and progenitor cells (HSPCs) employing an ex-*vivo* culture system has been used to recapitulate the features of various erythroid disorders, including CDA-I^13^. Here we used the immortalised erythroid HUDEP-2 cell line as a model system to investigate the interactions of endogenous CDIN1 in a CDA-I disease-relevant erythroid lineage. HUDEP-2 cells can be rapidly expanded as proerythroblasts (22.7 ± 5 h doubling time^20^) to generate large quantities of material for protein interactions studies during the early stages of terminal erythroid differentiation, which have previously been limited by inadequate cell numbers.

**Fig. 1:**
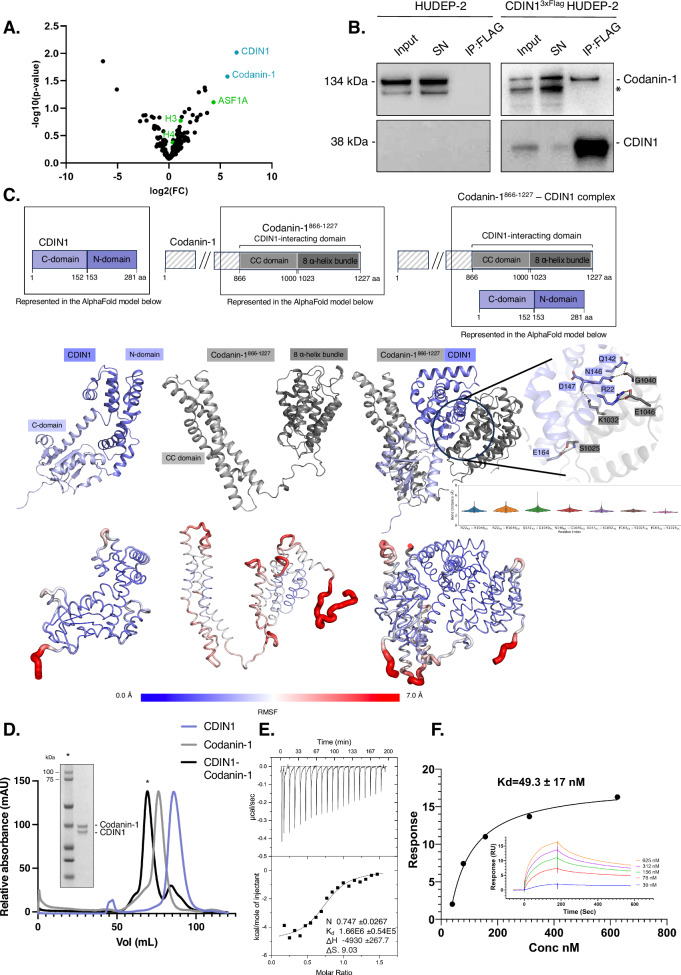
CDIN1 and Codanin-1 form a stable complex in vivo and in vitro. **A** Volcano plot of CDIN1^3*x*FLAG^ interaction partners identified by immunoprecipitation mass spectrometry. Codanin-1, the top hit, is labelled in light blue while the known Codanin-1-ASF1A-H3-H4 complex is highlighted in green. A list of binding partners is provided in Suppl. Data 1. The data were statistically analysed using PLGEM. **B** CDIN1^3*x*FLAG^ and Codanin-1 interact in the HUDEP-2 cell model. Endogenously Flag-tagged CDIN1 was immunoprecipitated and analysed by immunoblotting for CDIN1 and Codanin-1. A non-tagged cell line (HUDEP-2) served as a control for non-specific interactions (*n* = 3). Input: cell extract before immunoprecipitation; Supernatant (SN): Unbound fraction; IP: FLAG: Output fraction showing specific interaction partners of CDIN1^3*x*FLAG^, * indicates non-specific band, described in ref. ^4^. **C** Schematic diagrams and AlphaFold3-predicted structures of human CDIN1 (light blue, top left), the Codanin-1 C-terminus (residues 866-1227; grey) and the CDIN1-Codanin-1 complex (top right panel) showing predicted conformation of the complex compared to unbound subunits. Key binding interactions of the CDIN1-Codanin-1 complex during 3 $\times \,$10  ns equilibrium MD simulations are highlighted in the insets (*n* = 3 independent simulations). Embedded box plots show median (centre line), interquartile range (box), and minimum–maximum values (whiskers). Bottom panels show the corresponding computational modelling of results with the root means square fluctuations (RMSF) represented over 100 ns. RMSF colouring ranges from 0.00 to 7.00 Å and is displayed in a blue-white-red gradient corresponding to RMSF values. **D** Analytical size-exclusion chromatography demonstrates co-migration of CDIN1 and Codanin-1 as a complex of ~130 kDa (*n* = 3, see Suppl. Fig. 4D). Proteins in the CDIN1-Codanin-1 peak (labelled *) were confirmed by SDS-PAGE gel (as well as intact mass spectrometry, Suppl. Fig. 4B). **E** Isothermal titration calorimetry trace showing CDIN1 and Codanin-1 interaction is in the low µM range (K_d_= 1.66 ± 0.54 µM) and approximately 1:1 molar ratio. **F** Surface plasmon resonance identified a higher (nM) range interaction affinity (K_d_ = 49.3 ± 17 nM). The main plot shows a concentration-response curve fitted using a bivalent analyte model. The inset shows representative SPR concentration-response traces. Source data are provided as a Source Data file.

We generated a homozygous 5’ 3xFLAG-tagged CDIN1 at its endogenous locus in HUDEP-2 cells (CDIN1^3*x*F*lag*^ HUDEP-2). FLAG immunoprecipitation followed by mass spectrometry analysis (tandem mass MS/MS) was used to identify CDIN1 binding partners in an unbiased manner (Suppl. Data 1). The top hit for CDIN1 interaction was Codanin-1, consistent with previous observations of ourselves and others^5,6,12^, but where previous analyses were performed in a non-erythroid context and with overexpressed proteins. We noted the presence of an established Codanin-1 interaction partner, histone chaperone ASF1^4,12,17^, as well as histones H3 and H4 in the cohort of top hits (Fig. 1A, Suppl. Data 1). Immunoprecipitation experiments followed by immunoblot analysis confirmed the interaction of endogenous Codanin-1 and CDIN1 in the HUDEP-2 model, and this interaction was found to be resistant to high salt concentrations (500 mM), implying a tight association between the two proteins (Fig. 1B and Suppl. Fig. 1A).

### CDIN1 and Codanin-1 form a stable complex in in silico modelling studies

Despite reported attempts^10,11^, no experimental structural data is available for the CDIN1-Codanin-1 complex to date, likely due to the flexibility of the complex. To investigate the conformational effects of the CDIN1-Codanin-1 association on the overall structures of the proteins, we employed a combination of AlphaFold and molecular dynamics approaches^25^. AlphaFold3 (AF3) predicted the CDIN1 structure with a high predicted local distance difference test (pLDDT) score > 90 (Fig. 1C and Suppl. Fig. 1B). The CDIN1 protein consists of two domains: the N-terminal (N-domain, residues 1–152) and C-terminal domains (C-domain, residues 153-281) separated by a narrow cleft. The N-domain is predominantly α-helical, with eight α-helices and is connected to the C-domain by a single long α-helix. The C-domain adopts an α/β/α sandwich fold, with a central five-stranded mixed β sheet core flanked by α-helices on both sides. A Dali webserver^26^ structure-based search using the C-domain of CDIN1 revealed distant structural homology between this domain and a variety of Type II restriction nucleases, some of which have active sites predicted to belong to the same PD(D/E)XK superfamily of nucleases to which CDIN1 is proposed to belong^27^. Reassuringly, the conserved predicted catalytic residues (E166, D196, E214 and K216) of CDIN1, which define this family, cluster together above the central β-sheet, consistent with their predicted role in metal binding (Suppl. Fig. 1B, right panel and Suppl. Fig. 4C). This AF3 generated CDIN1 structure was further subjected to MD simulations, remaining stable during the simulation time (3 × 100 ns) as evidenced by root means square deviation (RMSD) analysis (RMSD around 1.5 Å, which converged after ~5 ns of simulation, Suppl. Fig. 1C, top left panel). The most flexible region, based on root means square fluctuations (RMSF) analysis, was observed at the C-terminal, comprising residues V274-A281 (Fig. 1C, bottom left panel). This finding is in agreement with the low pLDDT score <50 in AF3 predictions (Suppl. Fig. 1B). Additionally, two smaller flexible regions were identified, corresponding to the amino acid sequence H111-P116 and R187-D192 (Fig. 1C, left panel).

The AF3 predicted full-length Codanin-1 consists of a stable N-terminal subunit connected to a distinct eight α-helix bundle *via* long α-helices, termed the coiled-coil domain (CC domain). Several long, disordered loops are observed within and connecting the domains, especially within the N-terminus, as indicated by AF3’s pLDDT < 50 (Suppl. Fig. 2A). These observations were confirmed by MD simulations, where the full-length Codanin-1 model equilibrated well after ~5 ns and remained stable throughout the simulations (3 ×100 ns, RMSD of 1.0–1.5 Å). In contrast, the N-terminal loop of Codanin-1 was highly flexible, exhibiting an RMSF value of 10.0–15.0 Å (Suppl. Fig. 2C, top and middle left panel). The C-terminal region of Codanin-1 (residues 866-1227) is composed of a three-helix antiparallel coiled-coil domain (CC domain, residues 866–1000) linked by a flexible loop region to a terminal eight α-helix bundle (residues 1023-1202) consisting of four pairs of anti-parallel α-helices that show the characteristic repeat pattern and signature motifs of a HEAT repeat (Fig. 1C). This region, proposed as the binding region of CDIN1 by us and others^5,6^, had a high predicted aligned error (PAE) matrix in our AF model, suggesting it has a flexible association with the N-terminal subunit. This was confirmed through MD simulations (3 × 100 ns), which revealed flexibility at the linker region (residues 1000-1023) between the CC domain and the eight α-helix bundle. Flexibility was also observed in the C-terminal end loop (residues P1218 and S1227), while the rest of the structure remained relatively stable (Fig. 1C, middle panel and Suppl. Fig. 2C, right panel). To minimise structural variability, we remove this flexible loop and focused on the C-terminal interaction region of Codanin-1 and binding region of CDIN1 (Codanin-1 residues 866–1227) for our functional studies throughout (Fig. 1C and Suppl. Fig. 2A, B).

We next, examined the predicted structure of CDIN1 in complex with Codanin-1, using AF3^24^, focusing on full-length (residues 1-281) of CDIN1 and the C-terminal region of Codanin-1^866-1227^, hereafter referred to as Codanin-1 (Fig. 1C, right panel and Suppl. Fig. 3A). The two proteins form a compact complex, with the CDIN1 subunit interlocked between the CC domain and α-helix bundle of Codanin-1. The predicted complex displayed a high pLDDT score (> 90), although some regions showed lower values (pLDDT below 70. The PAE matrix indicates high confidence in the prediction of the relative positions of CDIN1 and the C-terminal Codanin-1 eight α-helix bundle, confirming previous findings that this region is essential for their interaction (Suppl. Fig. 3A). This is further reinforced by an overall interface predicted template modelling (ipTM) score of 0.84. MD simulations showed that the complex stabilised within 5 ns with an RMSD value of 1.5 Å and remained stable throughout the simulation time (3 × 100 ns, Suppl. Fig. 3B, top left). Amino acid fluctuations were notably low, particularly for Codanin-1, with only a small fraction of amino acids being more flexible, including terminal amino acids V626-A632 and the β-sheet connecting amino acids V324-D332. This result contrasts with the higher RMSD (3.0 Å) and higher fluctuations obtained when Codanin-1 was modelled alone (Compare Fig. 1C, right panel and Suppl. Fig. 3B to Fig. 1C, middle panel and Suppl. Fig. 2C, right panel). This stability is likely due to strong hydrogen-binding interactions between the helical N-domain of CDIN1 and Codanin-1, further highlighting the importance of interaction between the two proteins for mutual stability. Key hydrogen bonding interactions, including R22-P1041, R22-E1046, Q142-G1040, N146-G1040, A147-K1032 and E164-S1025, remained stable across all three MD trajectories (Suppl. Fig. 3C). Together, these findings highlight that the stable binding of CDIN1 is primarily facilitated by its interactions with the α-helix bundle of Codanin-1, while the CC domain and linker region remain in close proximity without forming a significant interface (Fig. 1C, right panel).

### Biophysical characterisation of the CDIN1-Codanin-1 complex

Despite the structural similarity of CDIN1 with the PD(D/E)XK superfamily of nucleases, no enzymatic activity has been reported to date. To further investigate this, we purified a stabilised construct of CDIN1 from *E. coli*, termed CDIN1_stable_, as early expression attempts of CDIN1 failed to produce soluble protein. We therefore employed a mutagenesis strategy, mutating three non-conserved residues (W237R, C223S and V18M) which in our hands and others^6,18^ significantly improved the yield of CDIN1_stable_ purifications. For clarity and to avoid confusion, the stabilised CDIN1_stable_ will be referred to as CDIN1 from hereon. We purified the individual CDIN1 and the C-terminal Codanin-1^866-1227^ protein subunits from *E. coli* to near homogeneity before combining these to form the CDIN1-Codanin-1^866-1227^ complex, hereafter referred to as CDIN1-Codanin-1 complex (Fig. 1D and Suppl. Fig. 4A). Despite the similar sizes of the CDIN1 and Codanin-1 constructs used, we noticed an earlier elution volume on gel filtration of Codanin-1, suggestive of a multimer, consistent with recent cryo-EM data^10,11^. When mixed in a 1:1 ratio, the two proteins co-migrate on size-exclusion chromatography (Fig. 1D) with an elution volume on an analytical column corresponding to an approximate mass of 130 kDa (and elution volume of 12.9 mL) (Suppl. Fig. 4D). This is consistent with the theoretical mass of a dimer-dimer complex (aabb complex, 149 kDa) (Fig. 1D and Suppl. Fig. 4D). The identity of the proteins was confirmed by intact mass spectrometry (Suppl. Fig. 4B). Isothermal titration calorimetry (ITC) experiments confirm a direct interaction between CDIN1 and Codanin-1 with an apparent affinity in the low µM range (K_d_ = 1.66 ± 0.54 µM) (Fig. 1E). We note that the ITC isotherm does not show a sigmoidal approach to the baseline as the analyse concentration increases, indicating a possible buffer mismatch or other pathology in the data. Nevertheless, the ITC data indicate an exothermic binding event with approximate 1:1 stoichiometry confirming that CDIN1 and the C-terminal 866-1227 amino acids of Codanin-1 form a stable high-affinity complex. We further characterised this interaction by surface plasmon resonance (SPR) (Fig. 1F). The data were fitted using a steady state affinity model showed a double-digit nM range interaction affinity (K_d_=49.3 ± 17 nM) although signs of mass transport limitation were evident in the data especially at higher protein concentrations.

In addition to the wild-type CDIN1-Codanin-1 complex (termed CDIN1-Codanin-1), we also purified a control complex bearing substitutions in the conserved core PD(D/E)XK catalytic motif residues of CDIN1, D196A and E214 A, referred to as CDIN1^D196,E214^. This variant is predicted to be catalytically inactive as determined by multiple sequence alignment with key members of the PD(D/E)XK superfamily of nucleases to which CDIN1 belongs while retaining the ability to interact with Codanin-1 (Suppl. Fig. 4A–C).

### CDIN1 is a novel RNA nuclease

**Fig. 2:**
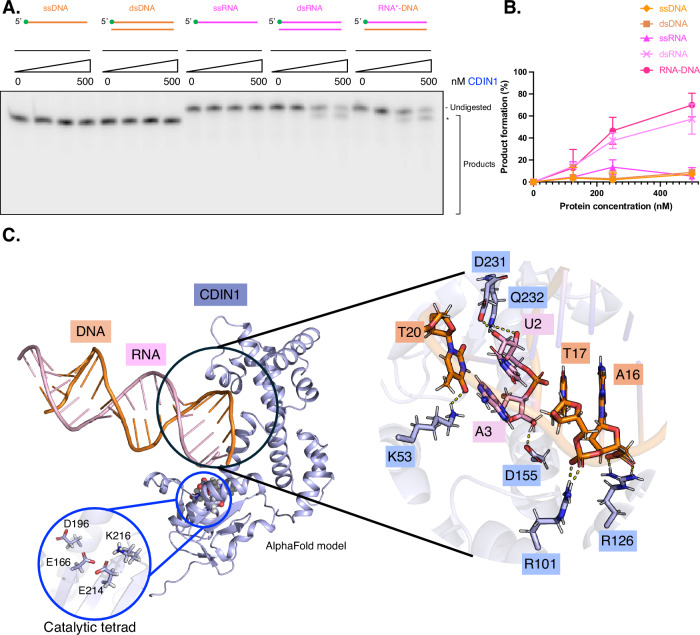
CDIN1 is a RNA nuclease. **A** CDIN1 is a RNAse able to incise 20-mer dsRNA-containing substrates in a single 2-nucleotide fashion (product *) from the 3’-end at higher protein concentrations ( > 250 nM). For DNA substrates (see Suppl. Table 1 for details), oligo 7 was labelled. For RNA substrates, oligo 3 was labelled. **B** Product formation (%) was quantified for Fig. 2A comparing CDIN1 nuclease activity of ssRNA, dsDNA, ssRNA, dsRNA and the RNA strand of an RNA:DNA hybrid as outlined in the Methods. All data are shown as mean ± s.e.m and three biological replicates (*n* = 3) were used for each substrate. CDIN1 shows activity on only dsRNA-containing substrates with a slight preference for the RNA strand of an RNA:DNA hybrid. **C** AlphaFold3 model of the complex structure of CDIN1 (light blue) bound to an RNA:DNA hybrid (RNA in light pink, DNA in light orange). The terminus of the RNA strand enters the active-site cleft of CDIN1 (represented as Van der Waals spheres) while the DNA strand forms stable hydrogen bonds with CDIN1, highlighted by the dashed lines. Increasing concentrations of protein (as indicated) were incubated with substrate at 37 ^o^C for 45 min and reactions were subsequently analysed by 20% denaturing PAGE to visualize product formation. Size of products was determined as shown in Suppl. Fig. 6A. Main products are labelled *, **, ***, **** and ***** corresponding to 18-mer, 16-mer, 14-mer, 13-mer and 12-mer respectively. All oligos used are indicated in Suppl. table 1A and 1B. Source data are provided as a Source Data file.

The PD(D/E)XK superfamily of nucleases, such as DNA repair nucleases XPF-ERCC1 and Mus81-EME1, process a variety of different DNA repair intermediate substrates. We initially tested any observable nuclease activity by incubating increasing concentrations of purified CDIN1 with a variety of simple 20-mer 5’-end terminally radiolabelled DNA substrates and observed no nucleolytic activity (Fig. 2A, B). We then tested the activity of CDIN1 on simple 20-mer 5’-end terminally radiolabelled RNA substrates and observed digestion products on double-stranded RNA and on the RNA strand only of an RNA-DNA hybrid, with a single cut present on these substrates at CDIN1 concentrations in excess of 250 nM employing 10 nM substrate (product * in Fig. 2A, B). This implies an RNA nuclease activity for CDIN1, with a preference for double-stranded RNA-containing substrates.

To further characterise key interactions between various nucleic acid substrates and CDIN1, we generated a CDIN1-RNA-DNA hybrid complex using AF3 (preferred substrate from Fig. 2A; Suppl. Fig. 5A). The AF3-derived structure was then used for 3 x 100 ns MD simulations, which showed that the complex equilibrated within 10 ns across all three replicas. Throughout the simulations, CDIN1 exhibited limited flexibility, as indicated by low RMSF values compared its apo state (Compare Suppl. Fig. 5B and Suppl. Fig. 1C). The RNA-DNA duplex adopts a canonical A-form structure and is predicted to position itself towards the cleft between the two domains of CDIN1 (Fig. 2C). Interestingly, while AF3 initially placed the DNA strand closest to the active site amino acid residues, MD simulations consistently re-orientated the nucleic acids, positioning the RNA strand toward the active amino acid residues E166, D196, E214 & K216 of CDIN1 (highlighted with VDW spheres). The phosphate backbone of the RNA strand is within approximately 8 Å of the predicted catalytic residues, in line with the observed nuclease activity on RNA. Furthermore, the RNA strand forms hydrogen bonding interactions with residues D155, D231, and Q232 in the C-domain of CDIN1 whilst the DNA strand interacts with N-domain residues K53, R101, and R126, positioned opposite the active site residues (Fig. 2C).

### CDIN1-Codanin-1 is an RNA nuclease complex where Codanin-1 modulates CDIN1 activity

Considering the strong evidence for CDIN1-Codanin-1 complex formation in vivo, supported by our in vitro analysis and molecular modelling (Fig. 1C–F), we generated an RNA-DNA hybrid substrate complex using AF3. The AF3-generated complex showed a high pLDDT > 90 for the protein complex whereas substrate binding predictions were less certain (Suppl. Fig. 5C), highlighting the intrinsic flexibility of the substrate-protein complex.

**Fig. 3:**
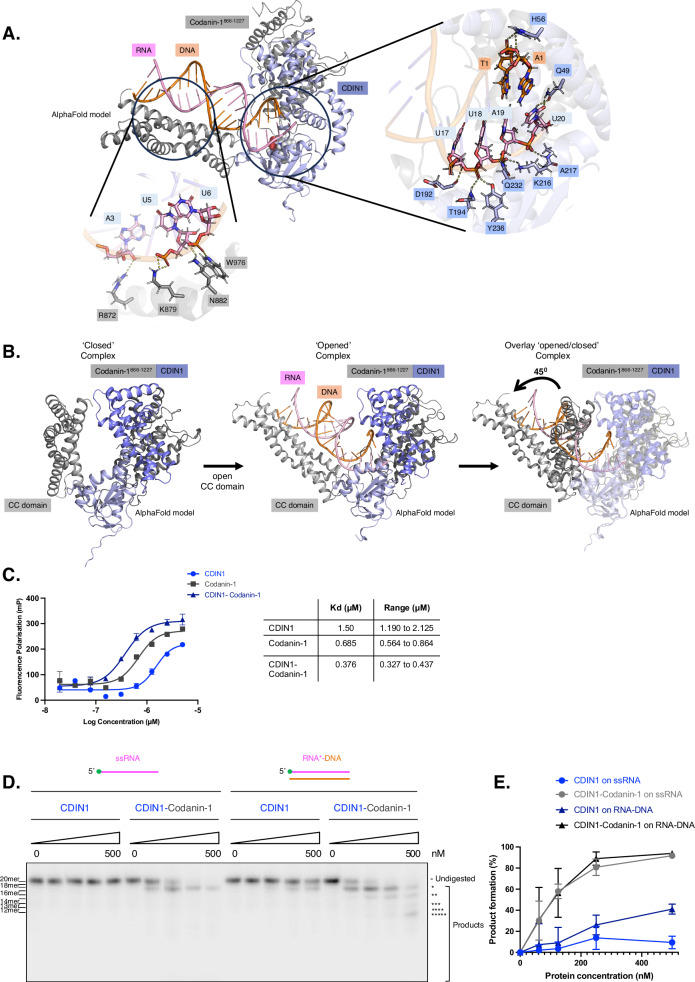
The CDIN1-Codanin-1 complex is an RNA nuclease where Codanin-1 modulates the activity of CDIN1. **A** AlphaFold3 model of the CDIN1 (light blue)–Codanin-1 (grey) complex bound to an RNA:DNA hybrid (RNA, light pink; DNA, light orange). The RNA substrate forms extensive hydrogen bonds with CDIN1 and additional contacts with Codanin-1, highlighted by dashed lines in the insets. **B** AlphaFold3 model of the CDIN1-Codanin-1 complex structure bound in apo ‘closed’ and RNA:DNA-bound ‘open’ conformations (RNA light pink, DNA light orange). Overlay of the two states shows an approximately 45° shift of the Codanin-1 CC-domain, enabling optimal RNA:DNA substrate binding. **C** Fluorescence polarisation analysis of dsDNA binding by CDIN1, Codanin-1, and the CDIN1–Codanin-1 complex (*n* = 3; mean ± SEM). CDIN1 displayed the weakest DNA-binding affinity (K_d_ = 1.50 µM), Codanin-1 bound with higher affinity (K_d_ = 0.685 µM). The CDIN1–Codanin-1 complex showed the highest affinity (K_d_ = 0.376 µM), indicating that Codanin-1 enhances substrate interaction. **D** Nuclease activity of CDIN1 versus the CDIN1-Codanin-1 complex. CDIN1 alone incised only the RNA strand of an RNA:DNA hybrid generating a single-cut product (*) at concentrations > 250 nM. In contrast, CDIN1-Codanin-1 cleaved both a 20-mer ssRNA substrate and the RNA strand of an RNA:DNA hybrid. On ssRNA, the complex produced * from > 62 nM. On RNA:DNA hybrids, the complex produced a ladder of cleavage products (*, **, ***, ****, ****) from the 3’-end of the RNA strand at 500 nM protein. Oligo 3 was labelled in all substrates. **E** Quantification of product formation from panel D comparing nuclease activities of CDIN1 alone and the CDIN1-Codanin-1 complex on ssRNA and RNA:DNA hybrids. Data are the mean ± SEM (*n* = 3). CDIN1 alone cleaved only the RNA strand of RNA:DNA hybrids, whereas the CDIN1–Codanin-1 complex cleaved both substrates more efficiently and processively. Proteins were incubated with substrates at 37 °C for 45 min and analysed by 20% denaturing PAGE. Products *, **, ***, **** and ***** correspond to 18-, 16-, 14-, 13- and 12-mer species, respectively. Oligos are listed in Suppl. Tables 1A and 1B. Source data are provided in the Source Data file.

To further investigate substrate-complex interactions, we performed 3 × 100 ns MD simulations, which showed that the complex binding pose equilibrates within 5 ns of MD simulation (RMSD values ~ 1.5 Å) and remained stable during the rest of the simulations (Suppl. Fig. 5D). RMSF analysis of the amino acid residues in the interacting region of CDIN1-Codanin-1 also showed a stable binding pose (Suppl. Fig. 5D) whilst the terminal loop, especially in three chains of amino acids i.e., P282-V291, L320-Q337 and R361-I386 of Codanin-1, exhibited higher fluctuations (Suppl. Fig. 5D). Compared to CDIN1-substrate complex alone, we observed a significant difference when looking at the substrate-protein interactions (Fig. 3A). Firstly, we find the nucleic acid orientation is shifted by around 10 degrees when Codanin-1 is present, allowing for deeper access of substrate into CDIN1’s active site cleft. The RNA strand now forms additional, strong hydrogen binding interactions with key residues, including U20-Q49 in the N-domain of CDIN1 and U20-A217, A19-K216, A19-Q232, U18-Y236, U18-T194 and U17-D192, located in the C-domain of CDIN1, which remain stable during MD simulations (Fig. 3A and Suppl. Fig. 5D). Further, two nucleotides of the DNA strand also form additional hydrogen binding interactions namely A1 and T1 with H56 in the N-domain of CDIN1 (Fig. 3A). The importance of the Codanin-1 subunit in substrate positioning is further highlighted when comparing the structures of apo ‘closed’ CDIN1-Codanin-1 and ‘open’ CDIN1-Codanin-1 complex with an RNA-DNA substrate (Fig. 3B). When no substrate is present, the CC domain of Codanin-1 (residues 866-1000) remains in close proximity of CDIN1 complexed with the terminal Codanin-1 eight α-helix bundle (residues 1023-1202) forming a tight, ‘closed’ structure. Upon substrate presence, we observe a clear shift of the CC domain (residues 866-1000) of about 45 degrees, likely mediated by the flexible linker region of Codanin-1 (residues 1000-1023), creating an ‘opening’ for the substrate and guiding it into CDIN1’s active site. Interestingly, the CC domain of Codanin-1 has a positively- and negatively-charged face and in our model, this positively-charged face forms an extensive contact with the RNA-DNA substrate (Suppl. Fig. 5E). Specific interactions include hydrogen binding interactions comprising U6 with W976 and N882, U5 with K879 and A3 with R872 as shown in Fig. 3A. These additional contacts (compared to substrate-CDIN1 alone) suggest the Codanin-1 subunit of the complex is involved in substrate stabilisation and helps present the substrate in a preferred orientation in the CDIN1’s active site (compare Fig. 2C and Fig. 3A). As our model suggests Codanin-1 is necessary for optimal substrate binding, we decided to assess the substrate binding capacity of the CDIN1 and Codanin-1 subunits alone compared to the CDIN1-Codanin-1 complex using a dsDNA substrate and fluorescence polarization (FP) assay (Fig. 3C). A DNA substrate was chosen for this experiment as CDIN1 has the ability to bind DNA but not digest it thus preventing substrate hydrolysis (Fig. 2A, Suppl. Fig. 7C and Suppl. Fig. 7D). CDIN1 alone bound to the probe with a K_d_ in the low µM range (K_d_=1.50 µM) whereas Codanin-1 alone showed a sub-µM range interaction affinity (K_d_ = 0.68 µM). This increase in affinity is expected considering Codanin-1’s large positively-charge interface (Suppl. Fig. 5E). Most notably, the highest affinity was observed between the substrate and CDIN1-Codanin-1 complex, with a sub-µM K_d_ of 0.37 µM, further validating our model that Codanin-1 plays a critical role in substrate mediation for CDIN1.

To test the nucleolytic activity of the CDIN-Codanin-1 complex, the nuclease activity of CDIN1 alone versus CDIN1-Codanin-1 complex was compared on 20-mer ssRNA and a CDIN1-favoured RNA-DNA hybrid substrate (Fig. 3D, E). The addition of the C-terminal fragment of Codanin-1 dramatically enhances and modulates the nuclease activity of CDIN1 on both substrates. The complex is not only able to digest the substrates at lower concentrations than CDIN1 alone, it also presents nuclease activity on ssRNA, a substrate that was not processed by CDIN1 alone (Fig. 3D, E). On the RNA-DNA hybrid substrate, CDIN1-Codanin-1 shows enhanced activity, processing the RNA-strand beyond a single 2-nucleotide cut (product *, the major product observed with CDIN1 alone) to generate a complex, laddering digestion pattern as the protein concentration increases (Fig. 3D and Suppl. Fig. 6A). The size of the digestion products was precisely determined using a single-nucleotide RNA ladder (Suppl. Fig. 6A). CDIN1-Codanin-1 digests RNA from the 3’-end displaying a distinct digestion pattern: at lower protein concentrations ( < 125 nM), an initial 2-nucleotide cut is visible (forming at 18-mer product, *). As the protein concentration is increased, the RNA is further cleaved in 2-nucleotide steps from the 3’-terminus until reaching a 14-mer length (products *, **, ***) which initiates single-nucleotide RNA laddering to a final 12-mer product (products ****, *****). No products smaller than a 12-mer were observed, potentially relating to a minimum footprint necessary for the enzyme to bind. Moreover, time course assays employing 125 nM CDIN1-Codanin-1 demonstrated that the emergence of digestion products is a function of duration of incubation of CDAN-CDIN1 with RNA substrate (Suppl. Fig. 6B).

### The CDIN1-Codanin-1 complex is a versatile RNA exonuclease

**Fig. 4:**
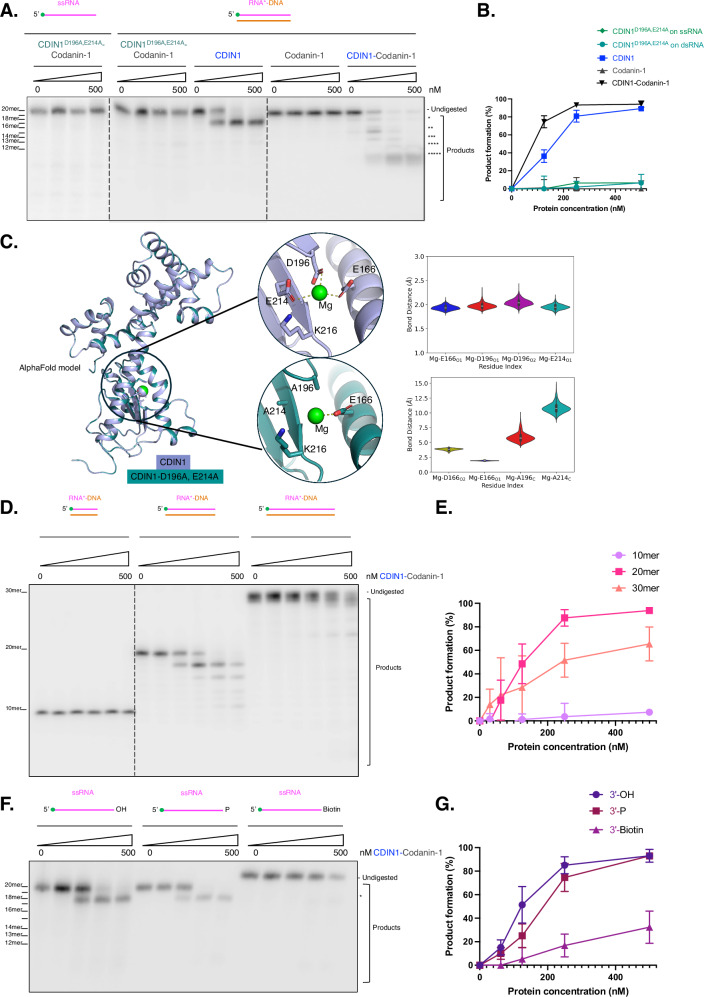
CDIN1 is a versatile RNA exonuclease. **A** Comparative activity gel of the predicted nuclease-inactive CDIN1^D196A,E214A^- Codanin-1 complex, CDIN1 alone and Codanin-1 alone on an RNA:DNA hybrid substrate. Digestion was observed only for CDIN1 (product *) and the wild-type CDIN1-Codanin-1 complex used as a positive control. No cleavage products were detected for CDIN1D^196A,E214A^-Codanin-1 or Codanin-1 alone. Green dot indicates the labelled strand. Oligos 3 and 7 were used. **B** Quantification of product formation from (**A**). Data are shown as mean ± SEM from *n* = 3. Digestion is only observed for CDIN1 alone (product *) and the wild-type CDIN1-Codanin-1 complex (products *-*****). **C** Overlay of the AlphaFold3 structure of wild-type CDIN1 (light blue) coordinated with Mg^2+^ (green sphere) and mutant CDIN1^D196A,E214A^ (teal), showing loss of Mg^2+^ coordination. Middle panel shows active site interactions in wild-type and mutant CDIN1. Stability of active-site interactions over 100 ns MD simulations (*n* = 3) are shown in right panels. Box plots show the median (centre line), interquartile range (box), and minimum–maximum values (whiskers). **D** CDIN1-Codanin-1 preferentially cleaves 20-mer RNA:DNA hybrid substrates. No activity was detected on a 10-mer substrate, while weak activity was observed on a 30-mer substrate. The 20-mer substrate showed the characteristic digestion products. Oligos 1, 3, and 14 were labelled. **E** Quantification of product formation from (**D**) comparing nuclease activity on 10-, 20-, and 30-mer RNA:DNA hybrids. Data are the mean ± SEM from *n* = 3. The complex displayed strong preference for the 20-mer substrate. **F** CDIN1-Codanin-1 functions as an RNA exonuclease complex requiring a free 3′ RNA terminus. No digestion occurred with a 3′-biotin-blocked substrate, whereas substrates containing 3′-hydroxyl or 3′-phosphate groups showed comparable activity. Data are shown as mean ± SEM (*n* = 3). **G** Quantification of data in (**F**). Proteins were incubated with substrates at 37 °C for 45 min and analysed by 20% denaturing PAGE. Products *, **, ***, **** and ***** correspond to 18-, 16, 14-, 13- and 12-mer species. Oligos are listed in Suppl. Table 1A, B. Source data are provided in the Source Data file.

In order to confirm that the observed nuclease activity is intrinsic to the CDIN1-Codanin-1 complex, several complementary approaches were employed. First, the activity of the predicted nuclease-inactive complex, where key residues D196 and E214 of the PD(D/E)XK catalytic motif were substituted (CDIN1^D196A,E214A^- Codanin-1) (Suppl. Fig. 4C), was assessed on the (favoured) 20-mer RNA-DNA hybrid substrate. As predicted, no activity was observed (Fig. 4A, B). Next, the nuclease activity of each individual component of the complex, namely CDIN1 alone and Codanin-1 alone, were assessed on the same substrate, where the CDIN1-Codanin-1 complex was used as a positive control. Nucleolytic digestion was only observed for CDIN1 and CDIN1-Codanin-1 complex, confirming that the enhanced nuclease activity of the complex is not due to any intrinsic nuclease activity of Codanin-1 or any contaminant within the Codanin-1 sample but rather, due to the direct association of the two factors (Fig. 4A).

Since we confirmed that CDIN1-Codanin-1 has an intrinsic nuclease activity, we next conducted an in-depth biochemical characterisation of the complex and the requirements for its RNA nuclease activity. As previous biochemical and structural studies imply that the nuclease activity of the PD(D/E)XK family of nucleases is dependent upon divalent metal cations, we investigated whether this is the case for CDIN1-Codanin-1. First, we determined which divalent cations support maximal activity of the complex. Magnesium, manganese and zinc promoted the RNase activity of CDIN1-Codanin-1, although it should be noted that at high concentrations zinc is reactive with RNA (Suppl. Fig. 6C). Consistent with a requirement for metal ions for activity, three metal-chelating agents, ethylenediaminetetraacetic acid (EDTA), ethylene glycol-bis(b-aminoethyl ether)-N,N,N’,N’-tetraacetic acid (EGTA), and ο-phenanthroline were strongly inhibitory (Suppl. Fig. 6D). For subsequent analyses, we chose to perform reactions in the presence of magnesium as this efficiently supports CDIN1-Codanin-1 activity while being abundant in mammalian cells. MD analysis shows that the metal bound CDIN1 structure is well equilibrated with small RMSD values in reference to starting structure and stays stable during the course of 100 ns MD simulations across all these replicas, similar to observations with apo CDIN1 (without metal) (Suppl. Fig. 7B). Interestingly, the Mg^2+^ binds to CDIN1 with three ligands with an approximately octahedral geometry coordinated by active site amino acids E166, D196 and E214 (Fig. 4C_,_ middle panel). In MD simulations, the structure of mutant CDIN1^D196A,E214A^ showed no significant difference in RMSD and RMSF values compared to the wild type (WT) CDIN1 (Suppl. Fig. 7B). However, these mutated residues altered the coordination sphere of Mg^2+^, resulting in the metal moving away from the active site, as evidenced by the increased distance of Mg^2+^ with carbon atoms of A196 and A214 at a distance of 6.5 and 11.5 Å respectively (Fig. 4C, right panel). Additionally, the overlay of the final equilibrated structural coordinates of WT and mutant CDIN1 shows notable conformation changes with an overall RMSD value of 3.12 Å (Suppl. Fig. 7A), which could potentially contribute to the loss of Mg^2+^ coordination in the mutant CDIN1^D196A, E214A^ protein. In addition, our simulation of both one-metal and two-metal ions (Mg^2+^) configurations in the presence of the hybrid RNA / DNA substrate shows that each arrangement is compatible with nuclease activity (Suppl. Fig. 14A, B). Both modes of Mg^2+^ coordination have been reported in related nuclease systems, supporting the plausibility of multiple catalytically relevant metal-binding states^28,29^.

To more definitively determine whether CDIN1-Codanin-1 activity is limited to RNA only, a variety of DNA substrates of various lengths and structures were tested on a broad range of complex concentrations. DNA substrates modelling DNA replication and recombination structures were prioritised, as notable members of the PD(D/E)XK family of nucleases, including DNA repair nucleases Mus81-EME1 and XPF-ERCC1, act on these substrates. These include structures mimicking replication forks, both with and without leading and lagging strands. In all cases, no DNase activity was observed, even at concentrations 10-fold higher than tested on the RNA substrates (Suppl. Fig. 7D). A DNA-RNA hybrid was also tested where the DNA strand was radiolabelled. Again, no DNase activity could be observed, further validating that CDIN1 activity is specific to RNA (Suppl. Fig. 7D). Interestingly, when performing electrophoretic mobility shift assays (EMSAs), wherein increasing concentrations of wild-type and mutant CDIN1-Codanin-1 (as indicated) were incubated with 1 nM RNA or DNA substrates, both complexes showed robust complex formation with both RNA and DNA substrates (Suppl. Fig. 7C). In contrast, digestion was only observed for the wild-type complex on RNA substrates and not DNA substrates (Fig. 3D and Suppl. Fig. 7D). This suggests CDIN1 differentiates between deoxyribose and ribose sugar-containing substrates for hydrolysis, while retaining the ability to bind to both deoxy- and ribo-nucleic acids.

Next, the activity of CDIN1-Codanin-1 on a range of structurally distinct RNA-containing substrates was assessed. First, the activity of CDIN1-Codanin-1 was compared on RNA substrates of different lengths to investigate a potential length preference. 10-mer, 20-mer, and 30-mer RNA-DNA hybrids were tested and revealed that the complex has a strong preference for the 20-mer substrates with no distinguishable digestion observed on any of the 10-mer substrates. Weaker activity (for protein concentrations above 250 nM) was observed on the 30-mer RNA-DNA hybrid (Fig. 4D, E). In order to better understand the complex digestion pattern of CDIN1-Codanin-1, its activity was compared on sequence-identical 20-mer ssRNA substrates containing either a 3’-hydroxyl, 3’-phosphate, or 3’-biotin moiety. Activity was observed only on the substrates containing the 3’-hydroxyl and 3’-phosphate but not the 3’-biotin moiety. The characteristic 2-nucleotide cut (product *) was only present on the RNA substrates with a free 3’-end, with CDIN1-Codanin-1 showing equal ability to process the 3’-hydroxyl and the 3’-phosphate substrates equally. In contrast, no activity was observed for the 3’-biotin substrate suggesting CDIN1-Codanin-1 has properties consistent with other RNA exonucleases whose activity is dependent upon the presence of an unblocked 3’-terminus (Fig. 4F, G). To further confirm this, the same substrates as detailed above were incubated with a classical RNA exonuclease, Exonuclease T (RNaseT)^30^. As expected, Exonuclease T was able to efficiently digest both the 3’-hydroxyl and the 3’-phosphate substrates but its activity was blocked by the 3’-biotin group, similar to CDIN1-Codanin-1 (Suppl. Fig. 8D). The requirement for a free RNA 3’- terminus (as opposed to a DNA 3’-terminus) was again demonstrated when testing the activity of CDIN1-Codanin-1 on ssDNA and dsDNA substrates containing an embedded ribonucleotide (analogous to the types of substrates processed by the ribonucleotide excision repair (RER) enzymes) and’Okazaki-like’ DNA replication substrates with a 5’-RNA section linked to a 3’-DNA section^31,32^. No digestion was observed, even at the highest concentrations in either case, suggesting the complex is not able to process any substrates containing a 3’-DNA terminus (Suppl. Fig. 8A, B). A common RNA-containing structural intermediate, primarily associated with transcription, is the R-loop^33,34^. Therefore, CDIN1-Codanin-1 activity was assessed on substrates mimicking R-loops, consisting of a DNA loop and a small 10-mer radiolabelled RNA fragment annealed within the loop region of the DNA. Results revealed that CDIN1-Codanin-1 is able to make a single incision within the RNA fragment, even though it is not able to nucleolytically incise the ssRNA fragment alone, which corroborates earlier results showing the enzyme requires a minimum length of substrate for digestion (Suppl. Fig. 8E). Furthermore, the ability of CDIN1-Codanin-1 to incise the RNA fragment is not dependent on the presence of a DNA loop structure, but rather on the longer bottom DNA strand which likely provides a scaffold for the enzyme to bind and overcome its minimum length requirement (Suppl. Fig. 8E). As a control, a D-loop structure was also tested where the short RNA fragment was replaced with a short ssDNA fragment. As expected, no activity is observed on any of the DNA components of this structure, further validating that CDIN1-Codanin-1 activity is specific to RNA only (Suppl. Fig. 8F).

Finally, the impact of common RNA modification 6-methyladenine, an artificial base modification 2-methyladenine, and the abundant variant RNA base inosine were tested on CDIN1-Codanin-1 nuclease activity. 6-methyladenine is the most common RNA modification, mainly present in messenger RNA, where it serves as a regulatory marker for translation and stability^35^. Inosine is present in tRNA molecules and is also involved in innate immune sensing through A-to-I editing^36^. When any of these modified bases were placed two ribonucleotides from the 3’-terminus of the 20-mer substrate, CDIN1-Codanin-1 activity was differentially affected by the presence of certain RNA modifications when compared with an unmodified RNA substrate (Suppl. Fig. 8C). Whilst the 6-methyladenine and the unmodified substrate were processed almost identically, the presence of the non-natural 2-methyladenine modification was slightly inhibitory, with digestion observed only at the highest protein concentration (500 nM). In contrast, the addition of the inosine base to the substrate was strongly inhibitory to CDIN1-Codanin-1, both quantitatively and qualitatively. Partial digestion can only be observed at the highest protein concentration (500 nM), with only a single incision present and no characteristic RNA laddering as seen on the unmodified substrate (Suppl. Fig. 8C).

We conclude that CDIN1 is a 3’-5’ RNA exonuclease, able to digest a variety of ss- and ds-RNA substrates, that requires association with Codanin-1 for its full range of nuclease activities.

### Validation of AF/MD structural model through site-directed mutagenesis

To overcome the lack of structural data of the CDIN1-Codanin-1 complex, we employed a combination of AlphaFold and molecular dynamics techniques to provide insight about the complex’s structure and how it relates to the observed nuclease activity.

**Fig. 5:**
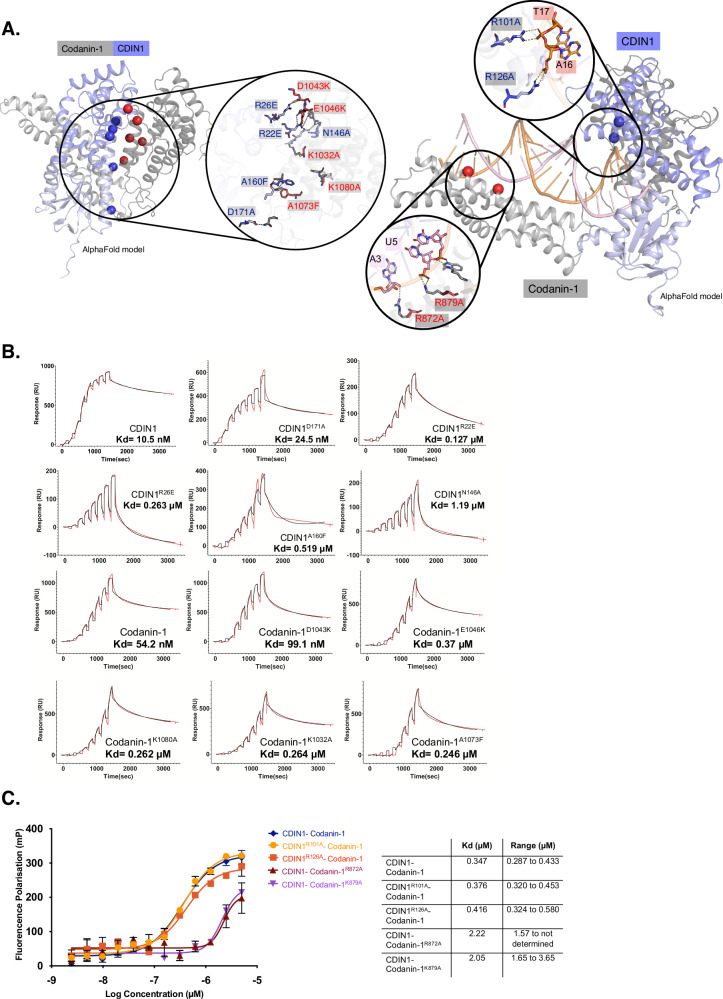
Validation of our AF/MD structural model through site-directed mutagenesis. **A** Right panel: AlphaFold3 model of the complex structure of CDIN1 (light blue) and Codanin-1 (grey) showing the positioning of the generated interaction mutants in CDIN1 (blue spheres) and Codanin-1 (red spheres). Insets show the predicted disruptions in hydrogen bonding and salt-bridges between the two sub-units caused by the mutations. Left panel: AlphaFold3 model of the complex structure of CDIN1 (light blue) and Codanin-1 (grey) bound to an RNA:DNA hybrid (RNA in light pink, DNA in light orange) showing the positioning of the generated nucleic-acid binding mutants in CDIN1 (blue spheres) and Codanin-1 (red spheres). Insets show the predicted disruptions in hydrogen bonding and salt-bridges between the proteins and substrate caused by the mutations. **B** Analysis of binding of interaction mutants in CDIN1 and Codanin-1 by surface plasmon resonance using single-cycle kinetics to examine interaction pairs. Mutations in CDIN1 affected binding to Codanin-1 more severely than mutations in Codanin-1 to CDIN1, with Kd as low as 100-fold less for the N146A mutant in CDIN1 compared to wild-type CDIN1 (K_d_=1.19 µM vs K_d_ = 10.5 nM). Mutations R22E, R26E and D171A in CDIN1 lowered the binding affinity to Codanin-1 between 2 and 20-fold (all within a sub- µM range) whereas mutation A160F lowered the binding affinity to Codanin-1 50-fold (K_d_=0.51 µM compared to K_d_ = 10.5 nM for wild-type CDIN1). For Codanin-1 mutants, apparent dissociation constants ranged from K_d_ = 99 nM for Codanin-1^D1043K^ to K_d_ = 0.37 µM for Codanin-1^E1046^, between 2 and 7-fold lower than wild-type Codanin-1 (K_d_=54 nM). **C** Analysis of DNA binding by fluorescence polarization of nucleic-acid mutant complexes CDIN1^R101A^-Codanin-1, CDIN1^R126A^-Codanin-1, CDIN1-Codanin-1^R872A^ and CDIN1-Codanin-1^K879A^ compared to wild-type CDIN1-Codanin-1 (*n* = 3, data represented as mean values +/− SEM). Only mutations in Codanin-1 affected DNA binding, with both showing an almost 10-fold reduction in binding affinity compared to wild-type. Source data are provided as a Source Data file.

Guided by our AF and MD models of the CDIN1-Codanin-1 complex (Fig. 1C), we performed site-directed mutagenesis generating mutants that would abolish or decrease the interaction between the two subunits. The point mutants R22E, R26E, N146A, D171A and A160F were chosen in CDIN1 and D1043K, E1046K, K1080A, K1032A and A1073F in Codanin-1: as shown in Fig. 5A, these mutations fall within the proposed protein-protein interface and are predicted to disrupt key hydrogen bonding or salt bridge interactions either through charge-reversal (e.g. R22E or D1043K), charge neutralisation (e.g. D171A or K1032A), change in polarity (e.g. change from polar N146 to non-polar A146) or by size-constraint (e.g. inserting a bulky F160 instead of a smaller A160 side chain). In order to investigate the proposed nucleic acid binding interfaces (Fig. 2C and Fig. 3A), we also generated point mutations in key charged residues on either CDIN1 or Codanin-1 predicted to mediate these interactions (R101A and R126A in the N-domain of CDIN1, and R872A and K879A in the CC domain of Codanin-1) (Fig. 5A). We purified individually residue-substituted subunits in *E. coli*, namely CDIN1^R22E^, CDIN1^R26E^, CDIN1^N146A^, CDIN1^D171A^, CDIN1^A160F^, CDIN1^R101A^, CDIN1^R126A^, Codanin-1^D1043K^, Codanin-1^E1046K^, Codanin-1^K1080A^, Codanin-1^K1032A^, Codanin-1^A1073F^, Codanin-1^R872A^ and Codanin-1^K879A^ to near homogeneity (Suppl. Fig. 9A). All substituted protein variants showed minimal changes in melting temperature compared to wild-type in a thermal shift assay, indicating correct protein folding (Suppl. Fig. 9B).

We first tested the ability of our predicted protein-protein interaction mutants to form Codanin-1-CDIN1 complexes. To this end, CDIN1^R22E^, CDIN1^R26E^, CDIN1^N146A^, CDIN1^D171A^, CDIN1^A160F^ were mixed in a 1:1 ratio with wild-type Codanin-1 and Codanin-1^D1043K^, Codanin-1^E1046K^, Codanin-1^K1080A^, Codanin-1^K1032A^, Codanin-1^A1073F^ were mixed in a 1:1 ratio with wild-type CDIN1 and analysed by analytical size-exclusion chromatography. In all cases, we observed some evidence for complex formation, with both proteins co-migrating on an analytical size-exclusion column, similarly to the wild-type CDIN1-Codanin-1 complex (Suppl. Fig. 9C), indicating that none of our predicted interaction mutations completely abolished the interaction between the two proteins. To quantitatively determine affinities, we further characterised these binding interactions by SPR (Fig. 5B). Due to the large number of protein combinations to test, we used single-cycle kinetics to examine interaction pairs rapidly, without the need for intermediate regeneration. Using this experimental set up, a significantly higher affinity was observed for the wild-type complexes (K_d_=10 nM with CDIN1 as analyte and K_d_ = 54 nM when Codanin-1 was the analyte) than our previous multi-cycle result of K_d_ = ~500 nM (Fig. 1F). This discrepancy can be explained by the relatively slow kinetic rates (association rate around 3 × 10^4^ M^−^^1^ s^−1^) which result in failure to reach equilibrium at lower concentrations and thus a systematic underestimation of the K_d_ when fit with dose response (as seen in Fig. 1F).

Our SPR results identify that all mutants generated lowered the affinity between CDIN1 and Codanin-1, albeit to different extents. Overall, mutations in CDIN1 affected binding to Codanin-1 more severely than mutations in Codanin-1 to CDIN1. For CDIN1, the N146A substitutions from a polar to a non-polar residue, showed the greatest effect, lowering the binding affinity to Codanin-1 approximately 100-fold, from a nM- range interaction for wild-type CDIN1 (K_d_=10.5 nM) to a low-µM range interaction affinity for CDIN1^N146A^ (K_d_=1.19 µM) (Fig. 5B and Suppl. Fig. 10A). The charge-modulating mutations R22E, R26E and D171A in CDIN1 lowered the binding affinity to Codanin-1 between 2 and 20-fold (all within a sub-µM range) whereas the bulky substitution A160F lowered the binding affinity to Codanin-1 50-fold (K_d_=0.51 µM compared to K_d_ = 10.5 nM for wild-type CDIN1). Interestingly, the CDIN1 mutants mostly affected the off rate of the interactions (from ~4 × 10^−4^ per second to ~2 × 10^−2^ per second) (Fig. 5B and Suppl. Fig. 10A). This parameter is independent of protein concentration indicating these effects are not influenced by any differences in protein quantification or purity.

For Codanin-1, all mutations displayed lower binding affinity to CDIN1 although less markedly than the CDIN1 mutants. Apparent dissociation constants ranged from K_d_ = 99 nM for Codanin-1^D1043K^ to K_d_ = 0.37 µM for Codanin-1^E1046^, between 2 and 7-fold lower than wild-type Codanin-1 (K_d_= 54 nM which is in accordance with the previously-measured multi-cycle analysis in Fig. 1F). In contrast to CDIN1 mutants, Codanin-1 mutants displayed a similar off rate, with slight differences in the association rate constants ( ~ 3 × 10^4^ M^−1^s^−1^ to ~3 × 10^3^ M^−1^s^−1^) (Fig. 5B and Suppl. Fig. 10A). Taken together, these results are consistent with our predicted interface derived from the AF/MD models. While substitutions within the proposed interface reduced complex formation, the magnitude of effects varied, suggesting that individual residues contribute differently to complex stability. Notably, no single mutation abolished the interaction, indicating that cooperative contributions from multiple residues likely stabilize the complex.

We next tested the DNA binding affinity (noting that the Codanin-1-CDIN1 complex binds duplex DNA efficiently without the confounding effects of digestion, Suppl. Fig. 7C) of CDIN1^R101A^, CDIN1^R126A^, Codanin-1^R872A^ and Codanin-1^K879A^, compared to the respective wild-types (Fig. 5C). Fluorescence polarization (FP) analysis revealed that the nucleic acid-binding affinity of complexes was affected most by Codanin-1 mutations, CDIN1-Codanin^R872A^ and CDIN1-Codanin-1^K879A^, both showing an almost 10-fold reduction in binding affinity compared to wild-type CDIN1-Codanin-1 complex (K_d_=2.22 µM compared to K_d_ = 0.347 µM) (Fig. 5C and Suppl. Fig. 10B). Interestingly, complexes with mutations in CDIN1, CDIN1^R101A^-Codanin-1 and CDIN1^R126A^-Codanin-1, showed no significant difference in substrate-binding affinity compared to wild-type complex, even though these mutants did appear to affect the DNA binding when measured alone (K_d_ =2.5 µM compared to K_d_ = 1.5 µM) (Suppl. Fig. 10C).

This data is consistent with the predictions from our structural modelling and supports a mechanism where the additional contacts formed by the CC domain of Codanin-1 with the substrate that we identified in our AF/MD model (compared to substrate-CDIN1 alone, see Fig. 3A) play an integral part in optimal substrate position into CDIN1’s active site cleft.

### CDA-I founder mutations Y94C and L178Q have detrimental effects on the nuclease activity of CDIN1

**Fig. 6:**
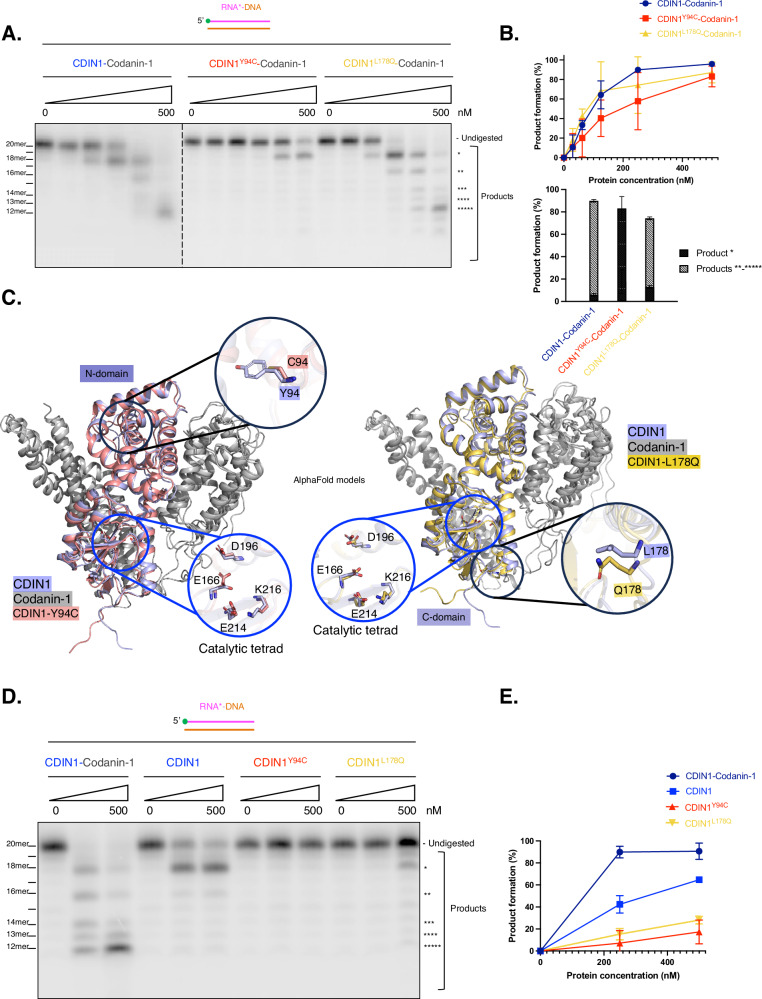
The CDA-I founder mutations Y94C and L178Q affect the nuclease activity of CDIN1. **A** Wild-type CDIN1-Codanin-1 activity on an RNA*:DNA hybrid compared to mutant CDIN1-Codanin-1 complexes bearing CDA-I patient Y94C and L178Q (CDIN1^Y94C^-Codanin-1 and CDIN1^L178Q^-Codanin-1 complexes, respectively). The L178Q mutation had a moderate effect, the Y94C mutation reduced the nuclease activity, both qualitatively and quantitively (only product * formed versus products *-***** for the wild-type complex). Green dot indicates the labelled strand. Oligos 3 and 7 were used. **B** Product formation (%) was quantified for Fig. 5A comparing wild-type CDIN1-Codanin-1 to CDIN1^Y94C^-Codanin-1 and CDIN1^L178Q^-Codanin-1. All data are shown as mean ± s.e.m and *n* = 3 performed for each substrate. The bar graph compares formation of product * vs product **-***** of wild-type CDIN1-Codanin-1, CDIN1^Y94C^-Codanin-1, and CDIN1^L178Q^-Codanin-1 complexes to highlight the qualitative difference in product formation for mutant CDIN1^Y94C^-Codanin-1 compared to wild-type complex. **C** Overlay of MD-equilibrated wild-type CDIN1-Codanin-1 structure (light blue and grey) with CDIN1^Y94C^-Codanin-1 (left panel, salmon) and CDIN1^L178Q^-Codanin-1 (right panel, yellow) highlighting the conformation of the proteins and positioning of the patient mutations in relation to the active site of CDIN1 (as seen in the insets). **D** Comparison of wild-type CDIN1 to mutants CDIN1^Y94C^ and CDIN1^L178Q^ activity on an RNA: DNA hybrid. Wild-type CDIN1-Codanin-1 complex was used as a positive control. CDIN1^Y94C^ and CDIN1^L178Q both^ display reduced nuclease activity when compared to wild-type CDIN1. Green dot indicates the labelled strand. Oligos 3 and 7 were used. **E** Product formation (%) was quantified for Fig. 5E comparing wild-type CDIN1- to CDIN1^Y94C^ and CDIN1^L178Q^. All data are shown as mean ± s.e.m and *n* = 3 were performed for each substrate. Increasing concentrations of protein (as indicated) were incubated with substrate at 37 ^o^C for 45 min and reactions were subsequently analysed by 20% denaturing PAGE to visualize product formation. Size of products was determined as shown in Suppl. Fig. 6A. Main products are labelled *, **, ***, **** and ***** corresponding to 18-mer, 16-mer, 14-mer, 13-mer and 12-mer respectively. All oligos used are indicated in Suppl. table 1A, B. Source data are provided as a Source Data file.

CDA-I is caused by mutations in *CDIN1*, whereby inherited missense mutations give rise to substitutions in the protein and cause anaemia with the characteristic ‘spongy’ heterochromatin phenotype in the erythropoietic lineage. We therefore investigated the effect of the two founder patient mutations in CDIN1, producing amino acid substitutions Y94C and L178Q, on the nuclease activity of CDIN1. To this end, we purified the individual patient substitution proteins CDIN1^Y94C^ and CDIN1^L178Q^ in *E. coli* before forming the CDIN1^Y94C^-Codanin-1 and CDIN1^L178Q^-Codanin-1 complexes to near homogeneity (Suppl. Fig. 11A). Similar to the wild-type complex, the two patient mutant complexes co-migrated on size-exclusion chromatography and the identity of the proteins was confirmed by intact mass spectrometry (Suppl. Fig. 11B, C). CDIN1^Y94C^-Codanin-1 and CDIN1^L178Q^-Codanin-1 were then incubated with an RNA-DNA hybrid and their activity compared to wild-type CDIN1-Codanin-1. Whilst the L178Q mutation did not diminish the nuclease activity significantly, the Y94C mutation reduced the nuclease activity, both qualitatively and quantitively (Fig. 6A, B). The mutant CDIN1^Y94C^-Codanin-1 complex was only able to form a single incision 2 nucleotides away from the 3’-terminus (product *), reminiscent of the activity of CDIN1 alone on this substrate (in Fig. 2A) and its activity was reduced compared to the more complex digestion pattern of the wild-type CDIN1-Codanin-1 observed at 500 nM protein concentration, possibly alluding to a disease mechanism (Fig. 6A, B).

Looking at our structural models, both residues are located away from the predicted interaction domain of CDIN1 and Codanin-1. Instead, the Y94 residue is located in the N-domain of CDIN1 with the tyrosine side chain approaching close to the substrate binding site (within approx. 5.5 Å). In contrast, the L178 residue is located on the periphery of the C-domain of CDIN1, forming a hydrophobic interaction with nearby non-polar residues and is distal from the nucleic acid interface (Fig. 6C). We therefore decided to investigate whether these observed changes in the nuclease activities could be explained by any effects on the conformation of the mutant complexes. The MD simulations of both mutant CDIN1^Y94C^ and CDIN1^L178Q^ structures, alone, and in complex with Codanin-1 showed that the overall structures are well equilibrated within a few nanoseconds. All structures remained stable for the rest of the MD simulations trajectories, with an average RMSD value of 1.5 Å across all three replicas for each. The CDIN1^Y94C^-Codanin-1 complex exhibited lower RMSF values for two chains of amino acids residues P282-R285 and A369-S381, while showing higher RMSF values for the other two amino acids loops Q314-Q320 and P424-E431 as compared to the CDIN1^L178Q^_-_Codanin-1 complex (Suppl. Fig. 12B, C).

An overlay of the final snapshot of 100 ns MD simulations of both mutants alone, in complex with Codanin-1, and by comparison to wild-type CDIN1, was performed to highlight the key differences. The overlay of WT complex with CDIN1^Y94C^-Codanin-1 complex (Suppl. Fig. 13A, left panel), showed minimal changes between WT CDIN1 and CDIN1^Y94C^, but the terminal loop of amino acids E316-Q335 of Codanin-1 exhibited higher conformational changes with an RMSD values of 3.40 Å with respect to the WT complex. CDIN1^Y94C^ alone exhibited fewer conformational changes with only one loop of amino acids (C14-S36) showing slightly higher RMSD with a value of 1.34 Å (Suppl. Fig. 13B, left panel). In contrast, the overlay of mutant CDIN1^L178Q^-Codanin-1 complex with WT complex (Suppl. Fig. 13A, right panel), showed almost no conformational changes with lower changes in the loop of amino acids E316-Q335 of Codanin-1 with an RMSD values of 2.87 Å (compared to CDIN1^Y94C^-Codanin-1 complex) whilst the overlay of CDIN1^L178Q^ with WT CDIN1 showed higher RMSD, including in two loops of amino acids C14-S36 and E110-P226 with RMSD value of 2.27 and 2.08 Å respectively (Suppl. Fig. 13B, right panel). Neither patient mutation led to any significant effects on the interacting region between CDIN1 with Codanin-1, wherein the key interactions remained stable during the course of 100 ns MD simulations across all three replicas for each system, as observed with the WT complex structure and in agreement with our successful in vitro patient mutant complex formation (Suppl. Fig. 12A).

Since our MD analysis revealed a differential effect of the patient mutations on the conformation of the proteins, with the Y94C mutation in CDIN1 primarily causing conformational changes to Codanin-1, whilst the L178Q mutation in CDIN1 induced changes within the CDIN1 structure itself, we also tested the nuclease activity of the individual CDIN1 subunits bearing these patient mutations. Notably, this revealed an effect of the CDIN1^L178Q^ patient substitution on the nuclease activity that was previously not observed when CDIN1^L178Q^ was complexed with Codanin-1. For the mutant CDIN1 subunits alone, both the Y94C and L178Q patient substitutions reduced the nuclease activity of CDIN1, where only very weak digestion is observed, even at the highest protein concentrations (Fig. 6E, F).

Taken together, we suggest that the L178Q mutant, which results in a hydrophobic to a hydrophilic residue substitution, may be affecting the stability of the CDIN1 protein as it is some distance from the substrate binding site and part of the hydrophobic core of CDIN1. In this case, we presume the association of Codanin-1 with CDIN1^L178Q^ might stabilise the protein, which is consistent with the restored nuclease activity, and the lower fluctuations observed in the MD simulations when CDIN1^L178Q^ is complexed with Codanin-1. The Y94C mutant is located on the periphery of the substrate-binding interface suggesting a potential role in substrate binding, which may explain the stronger defects observed in the activity assays. The restoration of low-level activity of CDIN^Y94C^ upon Codanin-1 complex formation reflects once again the important role of Codanin-1 as a modulator of CDIN1. The observed MD changes in the CC domain of Codanin-1 when complexed with CDIN^Y94C^ could explain why this mutant complex is only able to restore partial activity and further strengthens our theory that correct conformation of the CC domain is crucial for optimal substrate orientation into CDIN1’s active site.

## Discussion

In this study, we identify complex formation of the two CDA-I disease-proteins CDIN1 and Codanin-1 and demonstrate that the complex has RNA nuclease activity. Whilst sequence alignments recognised that CDIN1 is distantly related to members of the PD(D/E)XK family of nucleases, this work represents the first evidence of such enzymatic activity. An analysis of genetic co-dependencies using the DepMap platform^37^ further supports the role of this complex as an RNA nuclease. While the top hit for positively correlated co-dependent genes for *CDIN1* was *CDAN1* (and vice versa), consistent with CDIN1-Codanin-1 association, several RNases including the key RNase *RNaseH2* were also amongst the top results for *CDIN1*, supporting a potential RNase role (Suppl. Fig. 15A).

Two recent studies^10,11^ have attempted to define the structure of the higher-order complex containing ASF1-CDIN1-Codanin-1, but neither study was able to resolve the structure of CDIN1 and its interacting region on Codanin-1, likely due to the flexible nature of this complex. Therefore, we employed a combination of activity assays coupled with AlphaFold3 (AF3) modelling and molecular dynamics (MD) simulations to address this problem, revealing key insights into the molecular architecture and mechanism of action of CDIN1-Codanin-1. Our results suggest CDIN1 and Codanin-1 form a tight complex, likely in a dimer form. Recent cryo-EM studies describe ASF1-Codanin-1 multimers (dimers or more)^10,11^. It is not understood how the C-terminal regions of Codanin-1 used in our study may contribute to these dimer/multimer formations: AF3 modelling suggests a possible additional contribution provided by the CC domain which is distinct from the CC domain interaction with CDIN1 and RNA described here (Suppl. Fig. 16A). However, this part of the interface lacks experimental support and actually overlaps with the ASF1-A2 site in the cryo-EM structures, suggesting careful interpretation is needed^10,11^. As there is no overlap between the resolved cryo-EM structures and our AF/MD models, we tentatively suggest a model where both functional ends of Codanin-1 (namely the ASF1-Codanin-1 and the CDIN1-Codanin-1 end) are likely linked by the large flexible regions identified in our AF models. This would allow for both enzymatically-active proteins to be effectively accommodated by the scaffold Codanin-1. We also identify key residues involved in the binding interface between CDIN1 and Codanin-1 and provide functional support for our AF/MD approach using site-directed mutagenesis on these residues. Collectively, the data are consistent with the proposed interface, although the variable impact of individual mutations suggests residues contribute differentially to complex stability. Disruption of multiple residues may be required to more substantially destabilize the complex. Overall, this study supports the use of AF in combination with MD to guide experiments where structural data is not available.

We find CDIN1 to be a versatile RNA nuclease where its association with Codanin-1 modulates its activity, allowing for further product processing compared to CDIN1 alone. This observation is supported by our AF modelling combined with MD strategy. The CDIN1-Codanin-1 complex was uniquely able to process substrates with free 3’-RNA termini, despite being able to bind DNA and RNA substrates. The reasons for the restricted substrate selectivity remain unclear, but it seems likely the cleavage reaction may require a ribose 2’ hydroxyl, and the fine details of substrate discrimination will be a focus for future efforts. RNA:RNA duplexes and RNA:DNA hybrids produce a helical conformation that is distinct from the B-form which is typical of dsDNA, forming A-form and A-like form conformations, respectively. It is possible this difference in helical conformation plays a role in CDIN1-Codanin-1 substrate specificity by allowing the substrate to better orient in the active-site groove, although it should be noted that this may only be the case for CDIN1 alone as the complex retains the ability to process unstructured ssRNA molecules. This observation further highlights the importance of the association of CDIN1 and Codanin-1, wherein complex formation confers CDIN1 the ability to process ssRNA substrates as well as process dsRNA-containing substrates past the initial 2-nucleotide product (product *). We note our model suggests the positioning of 2nd and 3rd phosphate of the substrate within the CDIN1 active site is in accordance with the initial 2-nucloetide product (product *) predominantly seen as the first cleavage product. Upon association with Codanin-1, we note more extensive contact formation between CDIN1, Codanin-1, and the substrate, as well as a shift in substrate orientation. In particular, the CC domain of Codanin-1 stands out as a key modulator of substrate accessibility: through its large positively-charged surface and a footprint of about 1.5 helix turns, the CC domain of Codanin-1 appears to act as a substrate ‘feeder’ to CDIN1, positioning the 3’-RNA termini more optimally and further into the active site cleft of CDIN1. The footprint of the CC domain may offer an explanation as to why a strong preference was observed for the 20-mer substrates in this study, since smaller substrates may not be able to reach the active site when in contact with the CC domain. This model is further confirmed by the increased substrate-binding affinity of CDIN1-Codanin-1 as opposed to CDIN1 alone, highlighting the importance of Codanin-1 association. Further to this, the lower substrate-binding affinity of point mutants in key substrate-binding residues R872 and K879 in the CC domain of Codanin-1 reinforces this model. We note that a substrate positioning role for nuclease interacting partners has been observed in other nuclease complexes such as for DNA-PKcs and the endonuclease Artemis^38^. Presumably this type of model is useful to assure a tight regulation of the nuclease activity of the catalytic subunits, where un-refrained nuclease activity could have detrimental cellular effects.

By simulating the effects of CDA-I-associated disease mutations (Y94C and L178Q) on the CDIN1 structure, we were able to predict how these substitutions might alter conformational stability and functions of CDIN1. The study of a rare disease like CDA-I offers a unique opportunity to characterise essential pathways: *CDIN1* and *CDAN1* are both currently considered to be cell essential genes, and complete loss of CDIN1 activity could be lethal to cells especially considering Codanin-1 loss is incompatible with life^7,9^. It is therefore critical to understand the structure and function of this complex to improve patients’ outcome. Considering the critical cellular role of CDIN1 as well as the fact that the residues mutated in CDA-I (p. 94 and p.178) are not particularly well-conserved, we expect the effect of disease mutations on CDIN1 to be of moderate nature, which is in accordance with our results. We note that both mutations affected the nuclease activity of CDIN1 negatively but in both cases, the association of Codanin-1 has a positive effect on restoring the complex’s activity. Both patient mutations are not located in the predicted interface between CDIN1 and Codanin-1. The L178Q mutation is located on the periphery of CDIN1 and is part of the hydrophobic core. The resultant hydrophobic to a hydrophilic residue substitution is likely to affect the stability of the CDIN1^L178Q^ protein where the association of Codanin-1 is key to stabilisation. Conversely, residue 94 of CDIN1 is located within distance of the substrate-binding interface, where the mutation from a tyrosine to a cysteine residue has a detrimental effect on the nuclease activity. Interestingly, our MD simulations show that the Y94C mutation also leads to significant conformational changes in the CC domain of Codanin-1, likely contributing to mutant CDIN1^Y94C^-Codanin-1 complex’s partial activity compared to wild-type CDIN1-Codanin-1 and further highlighting the importance of the CC domain of Codanin-1 for substrate ‘feeding’.

Finally, it is important to address the question of why an RNA nuclease is present in Codanin-1-containing complexes, which are at times associated with ASF1 and non-chromatinised histones H3 and H4^4,10–12,17^. We note that DepMap^37^ cites *CDAN1* as a ‘common essential gene’, and *CDIN1* as ‘strongly selective’ for survival across a broad range of cell lines, rendering this question difficult to address in the cellular context meaning stringently controlled degron systems^39^ or similar selective expression systems, will be required to characterise the cellular phenotype including any chromatin assembly defects of cells fully deficient for either factor. Still, some (non-mutually exclusive) possibilities that explain the defective chromatin state in CDA-1 are: (i) It is established that RNA is an important structural component of chromatin^40,41^ and here CDIN1-Codanin-1 might play a role in regulating chromatin assembly by processing chromatin-associated RNAs^42^. By virtue of processing specific RNA intermediates, the CDIN1-Codanin-1 complex might facilitate the deposition and/or maturation of heterochromatin where defects in this process may lead to the characteristic ‘spongy’ heterochromatin phenotype of CDA-I patients^12,18^; (ii) R-loops are a common occurrence during normal gene expression and can also be pathological if persistent^33,34,43,44^. These might need to be digested and purged in order to promote chromatin maintenance or deposition^43–45^; (iii) It has been known for over 60 years that soluble histones, by virtue of their positive surface charge, will attract soluble nucleic molecules in the cytoplasm and nucleus, of which RNA is by far the dominant species^46–48^. It has been proposed that there might be a requirement to purge these in order that normal histone deposition can take place. It is provocative, therefore, that the CDIN1-Codanin-1 complex associated with ASF1, which is localised to both the nucleus and cytoplasm^4,10,11^, is perhaps involved in a pathway that shuttles histones H3 and H4 for their deposition. Future work, employing sophisticated cellular models that can address the dynamic temporospatial biology of this nuclease and RNA-protein cross-linking and immunoprecipitation (CLIP-based) methods^49^ to identify whether CDIN1 displays a selective capacity to bind specific classes of RNA molecule, potentially involved in chromatin assembly and maintenance, will help shed light on this point.

## Methods

### Immunoprecipitations (IP)

5 × 10^6^ HUDEP-2 cells (validated as described in ref. ^50^), endogenously 3xFLAG tagged at the *CDIN1* N-terminus were harvested and cell pellets washed twice with ice-cold PBS. Cells were then lysed on ice for 45 min using lysis buffer (50 mM Tris (pH 7.5), 150 mM NaCl, protease inhibitor (cOmplete mini, EDTA-free, Sigma), 2 mM Mg^2+^, 5 U Benzonase (Sigma), 0.5% NP-40 and 5% Glycerol). 1 mM EDTA was then added to each sample and samples incubated on ice for a further 15 min. Once lysis is complete, samples were centrifuged at > 20,000 x g for 20 min at 4 °C. The supernatant was applied to 100 μL of pre-washed FLAG-M2 magnetic beads (Sigma, M8823) and incubated on a rotator at 4 °C for a minimum of 2 h. This was followed by washing steps using wash buffer (50 mM Tris (pH 7.5), 1 mM EDTA, protease inhibitor (cOmplete mini, EDTA-free, Sigma), 0.5% NP-40, and either 150 mM, 250 mM or 500 mM NaCl as indicated). Immuno-precipitated protein was eluted using  3x FLAG peptide (F4799, Sigma) in TBS buffer (50 mM Tris (pH 7.5), 150 mM NaCl) as per manufacturer’s guidelines, and the supernatant collected. Samples were then analysed by western blotting.

### Cloning and site directed mutagenesis of CDIN1 and Codanin-1 constructs

We have previously reported the purification of full-length CDIN1 (C15ORF41) from baculovirus-infected insect cells^18^. The protein was unstable and precipitated upon storage. To generate a more stable protein, we aligned the sequences of CDIN1 from multiple animal species and introduced mutations at 46 sites with interspecies variance into the human sequence, individually and in combinations. We found that a triple mutant, W237R, C223S and V18M, could be expressed in *E. coli* to generate high yields of a stable protein. This stable version, termed CDIN1_S_, is referred to as CDIN1 for clarity. It forms the basis for all bacterially-expressed CDIN1 versions. The CDIN1 coding sequence was cloned into the vector pNIC28-Bsa4^51^ which appends a hexahistidine and a TEV protease cleavage site at the N-terminal. Further mutations were introduced by site-directed mutagenesis to generate a nuclease-inactive (CDIN1^D196A/E214A^) protein, versions bearing patient mutants (CDIN1^Y94C^ and CDIN1^L178Q^) and interaction-mutants (CDIN1^R22E^, CDIN1^R26E^, CDIN1^N146A^, CDIN1^D171A^, CDIN1^A160F^, CDIN1^R101A^, CDIN1^R126A)^.

Initial attempts to purify Codanin-1 from multiple constructs in *E.coli*, insect or mammalian cells failed to yield any protein. However, when co-infecting insect cells with baculoviruses expressing CDIN1 and Codanin-1, we observed a new fragment of ~34 kDa which mapped to the C-terminal of Codanin-1. Guided by this, we generated new constructs of Codanin-1. A construct encompassing amino acids 866-1227, in the vector pNIC-ZB^51^ yielded copious amounts of purified protein, referred to as Codanin-1. Further mutations were introduced by site-directed mutagenesis to generate interaction-mutants (Codanin-1^D1043K^, Codanin-1^E1046K^, Codanin-1^K1080A^, Codanin-1^K1032A^, Codanin-1^A1073F^, Codanin-1^R872A^ and Codanin-1^K879A^).

Site-directed-mutagenesis (SDM) was carried out using an ‘inverse’ PCR experiment, whereby an entire plasmid is amplified using complementary mutagenic primers with minimal cloning steps. The Herculase II Fusion DNA Polymerase (Agilent) was utilised and SDM PCR was performed to amplify the whole plasmid, according to the manufacturer’s instructions. The PCR product was then added to a standard KLD enzyme mix (NEB) reaction and was incubated at room temperature for 1 h, prior to transformation into chemically competent *E. coli* cells.

### Expression and purification of CDIN1, Codanin-1 and CDIN1-Codanin-1

Full-length CDIN1 was cloned into a pNIC28-Bsa4 expression vector containing a Histidine (His_6_) tag in a 22 amino acid N-terminal fusion peptide with a recombinant tobacco etch virus (rTEV) protein cleavage site. A C-terminal fragment of Codanin-1 (amino acids 866-1227) was cloned into a pNIC-ZB expression vector with an N-terminal His_6_ and Z-basic tags followed by a rTEV protease cleavage site. The following protocol was used to purify all wild-type and mutant versions of CDIN1 (CDIN1^D196A/E214A^, CDIN1^Y94C^, CDIN1^L178Q^, CDIN1^R22E^, CDIN1^R26E^, CDIN1^N146A^, CDIN1^D171A^, CDIN1^A160F^, CDIN1^R101A^, CDIN1^R126A^) as well as wild-type and mutant versions of Codanin-1 (Codanin-1^D1043K^, Codanin-1^E1046K^, Codanin-1^K1080A^, Codanin-1^K1032A^, Codanin-1^A1073F^, Codanin-1^R872A^ and Codanin-1^K879A^).

Initially, CDIN1 and Codanin-1 were purified separately. The proteins were generated by transformation into *E. coli* BL21 Rosetta2 cells and expression in Terrific Broth media supplemented with 30 μg mL^−1^ kanamycin and 25 μg mL^−1^ chloramphenicol. The cultures were incubated with shaking (170 rpm) at 37 °C until an OD_600_ of 2.0 was reached. Cultures were then transferred to an incubator at 18 °C for 30 min before induction with 0.3 mM isopropyl β-D-1-thiogalactoyranoside (IPTG) and incubated for 18 hours with shaking (170 rpm).

Cells were harvested by centrifugation at 4000 x g for 30 min at 4 °C. The cell pellets were resuspended in lysis buffer (50 mM HEPES pH 7.5, 500 mM NaCl, 10 mM imidazole, 5% v/v glycerol, and 1 mM tris(2-carboxyethyl)phosphine (TCEP) and lysed by sonication. The lysates were clarified by centrifugation at >20,000 rpm for 30 min, and the supernatants were loaded onto an equilibrated (lysis buffer) immobilised metal affinity chromatography column (IMAC) (Ni-NTA resin, GE Healthcare).

The immobilised proteins were washed with lysis buffer, wash buffer (50 mM HEPES pH 7.5, 500 mM NaCl, 30 mM imidazole, 5% v/v glycerol, and 1 mM TCEP) and eluted with elution buffer (50 mM HEPES pH 7.5, 500 mM NaCl, 300 mM imidazole, 5% v/v glycerol, and 1 mM TCEP). The protein-containing fractions were pooled and dialysed overnight at 4 °C in dialysis buffer (50 mM HEPES pH 7.5, 500 mM NaCl, 5% v/v glycerol, and 1 mM TCEP) supplemented with rTEV protease for cleavage of the N-terminal 6xHis tag on Codanin-1 only. Codanin-1 was then subjected to a second IMAC step to separate His-tagged rTEV protease, cleaved His tag, and untagged Codanin-1.

To form the CDIN1-Codanin-1 complex, His_6_-CDIN1 was loaded on an equilibrated IMAC and an excess of untagged Codanin-1 added to the same column to allow for on-column complex formation. To allow for better complex formation, low-salt buffer was used (50 mM HEPES pH 7.5, 150 mM NaCl, 10 mM imidazole, 5% v/v glycerol, and 1 mM TCEP). The immobilised complex was then washed with low salt wash buffer (50 mM HEPES pH 7.5, 150 mM NaCl, 30 mM imidazole, 5% v/v glycerol, and 1 mM TCEP) and eluted with low salt elution buffer (50 mM HEPES pH 7.5, 150 mM NaCl, 300 mM imidazole, 5% v/v glycerol, and 1 mM TCEP). Protein-containing fractions were pooled, concentrated and further purified by a Superdex 200 increase 10/300 GL column equilibrated with SEC buffer (50 mM HEPES pH 7.5, 150 mM NaCl, 5% v/v glycerol and 1 mM TCEP) at 0.8 mL/min. The purified complex was concentrated to 0.5 mg mL^−^^1^ and was snap frozen in liquid nitrogen for later use.

For purification of CDIN1 and Codanin-1 subunits individually, protein-containing fractions after the first IMAC step were pooled, concentrated and further purified by a Superdex 200 increase 10/300 GL column equilibrated with SEC buffer (50 mM HEPES pH 7.5, 150 mM NaCl, 5% v/v glycerol and 1 mM TCEP) at 0.8 mL/min. The purified protein was concentrated to 0.5 mg mL^−1^ and was snap frozen in liquid nitrogen for later use.

The presence of the CDIN1-Codanin-1 proteins was confirmed via SDS-PAGE, and ESI-Q-TOF (electrospray-ionisation quadrupole time-of-flight) mass spectroscopy (MS).

### Isothermal Titration Calorimetry (ITC) of CDIN1 and Codanin-1

Measurements were made using a MicroCal VP-ITC (Malvern, United Kingdom) in a buffer containing 10 mM Tris-HCl pH 8.0, 250 mM NaCl and 1 mM TCEP. Experiments were performed at 10 °C with mixing (1000 rpm stirring). The syringe was loaded with 300 µl of a 360 µM solution of Codanin-1 (amino acids 866–1227) which was titrated into the cell (1400 µl) containing 38 µM CDIN1. Following baseline equilibration 20 injections of 15 µl were performed with 240 s between each injection. The collected data were corrected for heats of dilution (measured separately by titrating Codanin-1 into buffer) and deconvoluted using the MicroCal Origin software to yield enthalpies of binding (ΔH) and binding constants (K_B_). Data were fitted to a single site model and thermodynamic parameters calculated using the formula (Δ*G* = Δ*H* - TΔ*S* = -RTln*K*_B_).

### Surface plasmon resonance (SPR) with CDIN1 and Codanin-1

Codanin-1 (amino acids 866-1227) binding to CDIN1 by SPR was performed on a Biacore s200 machine using a Series S CM5 sensor chip. Reference and active flow cells were activated with a 1:1 mixture of 0.05 M NHS / 0.2 M EDC for 7 min at 10 µl/min. CDIN1 was immobilised on the active flow cell by two 30 second injections of 750 nM CDIN1 solution in a 10 mM sodium acetate buffer at pH 6.0, corresponding to approximately 250 RU increase. Both flow cells were blocked by the injection of 1 M Ethanolamine hydrochloride pH 8.5 for 7 min. For interaction analysis of the wild-type complex in multi-cycle mode, an 8 ×2-fold dilution series of Codanin-1 (5000 nM highest concentration) was prepared an injected over the sensor surface for 180 s with a 400 s dissociation phase at a flow rate of 35 µl/min in multi-cycle mode in a buffer containing 20 mM HEPES pH 7.5, 150 mM NaCl. Regeneration was performed following each injection with a 60 second injection of a buffer consisting of 500 mM NaCl and 50 mM Tris pH 9.0. The data were fit as affinity data using Biacore S200 evaluation software using a 1:1 binding model.

Analysis of CDIN1 and Codanin-1 interaction mutants (CDIN1^R22E^, CDIN1^R26E^, CDIN1^N146A^, CDIN1^D171A^, CDIN1^A160F^, CDIN1^R101A^, CDIN1^R126A^, Codanin-1^D1043K^, Codanin-1^E1046K^, Codanin-1^K1080A^, Codanin-1^K1032A^, Codanin-1^A1073F^, Codanin-1^R872A^ and Codanin-1^K879A^) was performed in single-cycle mode in a buffer containing 20 mM HEPES pH 7.5, 150 mM NaCl. Approximately 2000 Ru of wild-type CDIN1 and wild-type Codanin-1 were immobilized onto a CM5 chip using amine coupling (as above). After surface equilibration, 8 successive injections of a 2- fold dilution series ranging from 30 to 5000 nM of mutant protein were injected onto the surface and a single dissociation phase was followed for 2000 s. Data were fit using Biacore S200 evaluation software using a 1:1 binding model with a correction for baseline drift.

### Generation of radiolabelled substrates

A total of 10 pmol of single-stranded RNA or DNA (Eurofins MWG Operon, Germany) were incubated with 6.8 pmol γ-^32^P-ATP (Perkin Elmer), and 10 U T4 PNK (ThermoFisher Scientific) at 37 ^o^C for 1 h then the solution passed through a P6 Micro Bio-Spin chromatography column (BioRad) for 4 min at 1000 x rcf. For all assays, a final substrate concentration of 10 nM was used unless indicated otherwise.

For double-stranded structures, radiolabelled RNA or DNA was annealed to unlabelled complementary molecules at a 1:1.5 ratio in 10 mM Tris-HCl (pH 7.5), 50 mM NaCl and 1 mM EDTA by heating the sample to 95 ^o^C for 3 min before cooling to room temperature. A detailed list of all oligonucleotide sequences is provided in Suppl. Table 1A, B.

### Nuclease assays

Nuclease assays were carried out in 10 μL reactions containing 50 mM HEPES pH 7.5, 50 mM NaCl, 5 mM MgCl_2_, 5% glycerol, 0.5 mM DTT and 10 nM RNA or DNA substrate and serially diluted two-fold CDIN1-Codanin-1 complex or individual proteins from 500 nM unless indicated otherwise. Reactions were incubated at 37 °C for 60 min, and quenched by the addition of 5 μL stop solution (95% formamide (v/v), 10 mM EDTA, 0.25% xylene cyanol, and 0.25% bromophenol blue) and boiling at 95 °C for 5 min.

Reactions were then analysed by 20% denaturing polyacrylamide gel electrophoresis (40% solution of 19:1 acrylamide:bis-acrylamide, BioRad) and 7 M urea (Sigma Aldrich) in 1 x TBE (Tris-borate EDTA) buffer. Electrophoresis was carried out at 525 V for 1.5 h; gels were subsequently fixed for 60 min in fixing solution (50% methanol, 10% acetic acid), and dried at 80 °C for two hours under a vacuum. The dried gels were exposed to a Kodak phosphorimager screen and scanned using a Typhoon 9400 instrument (GE).

Where applicable, gel-based nuclease assays were quantified by analysing the product formation (digestion) of the radiolabelled RNA substrate(s). Gel images collected on a Typhoon scanner were analysed in Image J to determine the amount of substrate remaining in the well (undigested) in comparison to the amount of substrate that entered the gel (product) reported as a percent digested. This was plotted versus protein concentration to indicate digestion rate. Error is standard error. Three or more gels were analysed for each substrate.

### Computational modelling

#### System preparation

Since no crystallographically resolved structures are available for the individual CDIN1 and Codanin-1 proteins, we used the UniProt^52^ AlphaFold 2^25^ predicted Monomers for CDIN1 (UniProt: Q9Y2V0) and Codanin-1(UniProt: Q8IWY9). The predicted Local Distance Difference Test (pLDDT)) per-residue score was used to evaluate the quality of the prediction. For CDIN1 pLDDT > 90% was obtained for 257 of the 281 amino acids. For Codanin1 the globular domain and C-terminal region showed pLDDT > 70%, but the N-terminal loop exhibited pLDDT values below 50%.

The initial docking simulations of CDIN1 with C-terminal (residues 866-1227) of Codanin-1 were performed using HADDOCK v2.4.^53^. However, subsequent equilibrium molecular dynamics (MD) simulations showed that the binding pose was not stable, suggesting that the generated complex might not accurately represent the true interactions between the proteins. We then used AlphaFold 3 (accessed on 09-05-2024^24^), which allowed the prediction of the interacting pose of CDIN1 with C-terminal of Codanin-1, as well as DNA and RNA substrates. Since the nuclease activity of the PD(D/E)XK family depends on divalent metal cations, we investigated Mg^2+^ binding to CDIN1 using the Metal Ion-Binding online server (Lin et al. 2026).

For all the generated structures, the protonation and rotameric states of titratable residues were assigned at pH 7.4 based using MolProbity^54^, H + + ^55^, and visual inspection. Lys and Arg residues were modelled as positively charged, Asp/Glu in their anionic form, and histidine amino acids were all assigned to be neutral and singly protonated on either N_d_ or N_e_ atoms. The mutation study of different amino acids was constructed using the Dunbrack backbone dependent rotamer library^56,57^ within UCSF Chimera software^58^.

### MD simulations

Model structures and their binding complexes were prepared using the LEaP module of AmberTools22^59^. The protein was modelled with the ff14SB force field^60^ while DNA and RNA substrates were modelled with OL21^61^ and OL3^61^ force field respectively. Further, the solvation of each model system was accomplished by the construction of a truncated octahedral box of TIP3P^62^ water using LEaP, with a 10 Å distance extending from protein structures to the edge of the box, which was further neutralised by adding Na^2+^ ions whose parameters were taken from Joung and Cheatham^63^. The non-bonded interactions of Mg^2+^ ion were represented by the Lennard-Jones potential, and parameters were taken from Allnér et al.^64^. Full systems set-ups are detailed in Suppl. Table 2.

All the classical MD simulations were carried out AMBER 22 software^59^. The systems were initially minimised using 2000 steps of steepest decent minimisation with 5.0 kcal mol^−1^ as a restrains potential on heavy atoms (C, N, O, S) of the protein and allowing relaxation of solvent and ions. After that, the systems were heated at 400 ps by raising the temperature to 298.15 K in an NVT ensemble using a Langevin thermostat^65^, with the collision frequency of 5 ps^−^^1^ and backbone atoms were restrained with the force constant of 5.0 kcal mol^–1^. NPT equilibration was carried out for a total of 1 ns using Berendsen barostate^66^ and gradually removing the backbone restraints. Furthermore, three repeats of 100 ns NPT MD simulations have been carried out for each system with random seed generator using 2 fs time step, SHAKE algorithm^67,68^ on hydrogen atoms and 10 Å non-bonded cut-off for van der Waals interactions with periodic boundary conditions. While the long-range electrostatic interactions were calculated using Particle Mesh Ewald (10 Å cut-off). Data were stored at every 100 ps and analysed and visualised with CPPTRAJ and PYTRAJ (Darden, York, and Pedersen 1993), MDAnalysis^69^, VMD software^70^ and PyMOL (open source, v.2.3.0) (The PyMOL Molecular Graphics System, Schrödinger LLC).

## Supplementary information

Supplementary Information

Description of Additional Suppl. Files

Supplementary Data 1

Transparent Peer Review file

## Source data

Source Data

## Data Availability

The data supporting the findings of this study are available from the corresponding authors upon request. Source data for this paper can be found at GitHub (https://github.com/HafizSaqibAli/CDIN1-Codanin1-RNA-Nuclease) and is also provided with this paper. Proteomic mass-spectrometry data is available at ProteomeXchange repository (http://www.proteomexchange.org/) with PRIDE accession number PXD078989. Source data are provided with this paper.

## Code availability

Source code for this paper can be found at GitHub (https://github.com/HafizSaqibAli/CDIN1-Codanin1-RNA-Nuclease).
