# Supplementary material for: CDIN1-Codanin-1 complex defective in congenital dyserythropoietic anaemia type I is an RNA nuclease
Source: Nat Commun. 2026 Jul 2;17:8253. doi: 10.1038/s41467-026-74766-7 (PMC13463051; doi:10.1038/s41467-026-74766-7)
Supplement: Supplementary file 1 [file 41467_2026_74766_MOESM1_ESM.pdf]

SUPPLEMENTARY METHODS

Mass-Spectrometry (MS) Sample preparation

Samples were digested on-bead, where immobilized proteins were denatured with 8M urea in ammonium bicarbonate buffer (100 mM) for 10 min at room temperature. After denaturation, cysteines were reduced with TCEP (10 mM) for 30 minutes at room temperature and alkylated with 2-Chloroacetamide (50 mM) for 30 minutes at room temperature in the dark. Samples were then pre-digested with LysC (1 μg/100 μg of sample) for 2 hrs at 37°C. Prior to overnight digestion with trypsin (1 μg/40 μg of sample) at 37°C, urea was diluted down to 2M in ammonium bicarbonate buffer (100 mM) and calcium chloride was added at 2mM final. The next day, tryptic digestion was stopped with the addition of formic acid (5%). Digested peptides were centrifuged for 30 minutes at 13,200rpm at 4°C to remove undigested material. Supernatant was loaded onto handmade C18 stage tip, pre-activated with 100% acetonitrile, by centrifugation at 4,000rpm at room temperature. Peptides were washed twice in TFA 0.1%, eluted in 50% acetonitrile / 0.1% TFA and speed-vacuum dried. Dried peptides were resuspended into 5% acetonitrile / 5% formic acid before LC-MS/MS analysis. Peptides showing traces of detergent were cleaned-up using SP3 method as described in Hughes et al. 2019 .

Liquid Chromatography-MS/MS

Peptides were separated by nano liquid chromatography (Thermo Scientific Ultimate RSLC 3000) coupled in line a Q Exactive mass spectrometer equipped with an Easy-Spray source (Thermo Fischer Scientific). Peptides were trapped onto a C18 PepMac100 precolumn (300*μ*m i.d.x5mm, 100Å, ThermoFischer Scientific) using Solvent A (0.1% Formic acid, HPLC grade water). The peptides were further separated onto an Easy-Spray RSLC C18 column (75um i.d., 50cm length, Thermo-Fisher Scientific) using a 60 minutes linear gradient (15% to 35% solvent B (0.1% formic acid in acetonitrile)) at a flow rate 200nl/min. The raw data were acquired on the mass spectrometer in a data-dependent acquisition mode (DDA). Full-scan MS spectra were acquired in the Orbitrap (Scan range 350-1500m/z, resolution 70,000; AGC target, 3e6, maximum injection time, 50ms). The 10 most intense peaks were selected for

higher-energy collision dissociation (HCD) fragmentation at 30% of normalized collision energy. HCD spectra were acquired in the Orbitrap at resolution 17,500, AGC target 5e4, maximum injection time 120ms with fixed mass at 180m/z. Charge exclusion was selected for to 20 s.

MS Data Processing Protocol

Tandem mass (MS/MS) spectra were searched using Sequest HT in Proteome discoverer software version 1.4 were searched against a protein sequence database containing 20,602 protein entries, including 20,319 *Homo sapiens* proteins (Uniprot release from 2024-02-16) and 283 common contaminants. During database searching cysteines (C) were considered to be fully carbamidomethylated (+57,0215, statically added), methionine (M) to be fully oxidised (+15,9949, dynamically added), all N-terminal residues to be acetylated (+42,0106, dynamically added). Two missed cleavages were permitted. Peptide mass tolerance was set at 50ppm on the precursor and 0.6 Da on the fragment ions. Data was filtered at FDR below 1% at PSM level. Label free quantification was performed by Normalised Spectral Abundance (NSAF) as previously described (Florens et al. 2006), as the number of spectral counts (PSM) that identify a protein, divided by the protein length (L), the PSM/L value represents the SAF, which is then divided by the sum of PSM/L for all proteins in the experiment. NSAF values were calculated after common contaminants being removed. For better visualization of the data, NSAF values were multiplied by 100 (NSAF·100). Statistical analysis was performed by PLGEM (Pavelka et al. 2008) and volcano plots generated in GraphPad Prism version 10. All data is available online on PRIDE and in Suppl. Table 2A-D.

HUDEP-2 cell culture

HUDEP-2 cells were maintained as proerythroblasts at a density of 0.5×10^6 cells/mL to 2×10^6 cells/mL at 37°C and 5% CO_2. Every 48 hours, cells were counted, washed with PBS (300 x *g*, 5min) and resuspended in "expansion media" (StemSpan SFEM) supplemented with L-Glutamine (2mM), Penicillin/Streptomycin (100U Penicillin and 100μg/mL Streptomycin), human Stem Cell Factor (SCF) (50ng/mL), erythropoietin (3 IU/mL) and Dexamethasone (330μg/L). Doxycycline was added fresh to the culture at

the time of passaging, at a final concentration of 2μg/mL, due to the low stability of the compound.

Generation of CDIN1^{3xFlag} HUDEP-2

HUDEP-2 (Human Umbilical cord Derived Erythroid Progenitor-2) cells were obtained from the Cell Engineering Division, RIKEN BioResource Center, Tsukuba, Japan. Generation of FLAG-tagged CDIN1 at its endogenous locus in HUDEP-2 cells (CDIN1^{3xFlag} HUDEP-2) was conducted as per Moir-Meyer et al. 2018 with the following modifications: 24 hours prior to transfection, cell density was adjusted to 0.5x10^6 cells/mL in expansion media with 2μg/mL Doxycycline. On the day of transfection, cells were counted, 3.25x10^6 cells were aliquoted in a 15mL Falcon tube, washed with 8mL PBS and spun at 300 x *g* for 5 min. Supernatant was discarded, cells were resuspended in 1mL PBS and transferred to a 1.7 mL microfuge tube. Cells were spun at 300 x *g* for 5 min, supernatant discarded and cells were washed again with PBS. 199bp single stranded oligonucleotide donors (ssODN, IDT) were resuspended in nuclease-free H_2O at a final concentration of 200 μM. The ssODN sequence for the introduction of the 3x FLAG-tag at the 5' end of *CDIN1* was CTCATCCCTCGGCACCAAGGCTACTTGAGCCCCAGGGTGTTTTTTCCTTGTTCC CGCCACCTCCTGGTCCCTGGCCAAACATGGACTACAAGGACCACGACGGTGAC TACAAGGACCACGACATCGACTACAAGGACGACGACCCTCCACCGCCTATACT GACCAAAGCTCAGTACGACGAGATAGCCCAGTGCCTAGT. Transfection mix was prepared by mixing 9 μL of ssODN, 7.5μg pX458 plasmid, 18.8 μL CD34 supplement and 81.2 μL CD34 buffer (Lonza, Germany). The small guide RNA (sgRNA) for the introduction of the 3x FLAG-tag at the 5' end of *CDIN1* was GCTTTGGTCAGTATCATGT. Supernatant was carefully discarded, and cell pellets were resuspended using the transfection mix by gently pipetting up and down 3 times to avoid cell clumps. 110μL was transferred to an Amaxa 2B cuvette, care was taken not to introduce air bubbles. Cells were electroporated using the U-08 protocol on an Amaxa Nucleofector 2B machine. Immediately after electroporation, expansion media with 2μg/mL Doxycycline was added to the cuvettes and cells were transferred to 6-well plates at 1x10^6 cells/mL. 24 hours post-transfection, cells were assessed for GFP fluorescence via microscopy and 48 hours post-transfection, cells were counted, washed and resuspended in expansion media at 1x10^6 cells/mL. Fluorescence Activated Cell Sorting (FACS) was performed at 72 hours post-transfection.

Transfected cells were counted and washed with 5 mL PBS and centrifuged at 300 x *g* for 5 min. Cells were resuspended at $1x10^6$ cells/mL in 90% PBS/10% FBS and 1μg/mL Hoechst 33258. $1x10^5$ untransfected control cells were prepared as above. Untransfected cells were used to determine the population gating on a BD FACSAria Fusion machine. Transfected cells from the $Hoechst^-/GFP^+$ gate were single-cell sorted in 20μL expansion media + 2X Dox in Terasaki plates (Greiner Bio-One) and placed in a humidified chamber in an incubator. 5μL of expansion media + 4μg/mL Doxycycline was added 2 days after cell sorting and every other day in order to provide fresh media until more than 100 cells could be counted in each well. Clones were expanded daily until a sufficient number of cells could be frozen down and aliquoted for subsequent genotyping. To obtain genomic DNA ~$1x10^5$ cells per clone were lysed overnight in 50mM Tris pH 8.0, 1mM EDTA, 0.5% Tween-20 and 200μg/mL Proteinase K. Proteinase K was heat-inactivated at 95ºC for 10 min and DNA lysates were stored at -20ºC. For genotyping, 1 μL cell lysate was added to a PCR master mix containing 2 units FastStart Taq DNA Polymerase (Roche, UK), 3μL 2mM dNTPs, 1 x FastStart Taq Buffer, 0.5 μM forward and reverse primers spanning about 350 to 400bp around the cut site. The primers used for the introduction of the 3x FLAG-tag at the 5' end of *CDIN1* were: forward CTCCGCCCCGCTTTTGCAGCTAG and reverse GACACGTGGTTCCTAAGCGCTGCC. Reactions were placed in a thermocycler at 95ºC for 3min, then 30 cycles of [95ºC for 30 seconds, Tm for 30 seconds, 72ºC for 45 seconds] then 72ºC for 10min and 12ºC until use. Presence of amplification products determined using 2% agarose gels stained with SYBR Safe (1:10,000; ThermoFisher Scientific) or GelRed (1:100 000; Biotium). All amplification products were prepared for Sanger sequencing by removing dNTPs and dephosphorylation of residual primers. For sequencing 0.5μL primer, 0.4μL BigDye Terminator v3.1 ready reaction mix (ThermoFisher Scientific), 1.852μL 5X sequencing buffer and 2.248μL nuclease-free H_2O were added and amplified. Samples were then ethanol-purified and stored in 15μL HiDi Formamide prior analysis using and AB3730 capillary sequencing machine (Applied Biosystems). Clones showing a sequence introduced were revived and used for downstream experiments.

Generation of a single nucleotide RNA ladder

10 pmol of 5' radiolabelled single-stranded RNA was incubated in 50 mM potassium acetate (pH 7.9), 20 mM Tris-acetate (pH 7.9), 10 mM magnesium acetate (pH 7.9) and 1 mM DTT for 2, 5, 10, and 15 min at 95 °C respectively. 5 µL of stop solution (95% formamide, 10 mM EDTA, 0.25% xylene cyanol, 0.25% bromophenol blue) was added to each sample and samples combined to generate ladder.

Electrophoretic Mobility Shift Assay (EMSA)

EMSA reactions were carried out in 10 µL reactions containing 50 mM HEPES pH 7.5, 50 mM NaCl, 5% Glycerol, 0.5 mM DTT, 50 mM L- arginine, 0.01% Tween20 and 1 nM RNA or DNA substrate with either 20 mM Mg^{2+} or 5 mM Ca^{2+} and serially diluted two-fold CDIN1-Codanin-1 complex or individual proteins from 500 nM unless indicated otherwise. The reaction was incubated on ice for 15 min before the addition of 5 µL ice-cold loading buffer (50% glycerol, 1 mg/mL bromophenol blue).

Reactions were then analysed by 6% non-denaturing polyacrylamide gel electrophoresis in 0.5 x TBE (Tris-borate EDTA) buffer. Electrophoresis was carried out at 4 °C at 100 V for 3 hours in 0.5 x TBE; gels were subsequently fixed for 10 min in fixing solution (50% methanol, 10% acetic acid), and dried at 80 °C for two hours under a vacuum. The dried gels were exposed to a Kodak phosphorimager screen and scanned using a Typhoon 9400 instrument (GE).

Thermal shift assay

Thermal shift assay was used to assay correct protein folding and stability of CDIN1 and Codanin-1 point mutants. CDIN1 and Codanin-1 mutant proteins were diluted to 5 μM in assay buffer (10 mM HEPES, 150 mM NaCl) containing Sypro Orange (1000x) and incubated on ice for 30 min. Melting curves were obtained on an Mx3005p qPCR machine (Agilent), ramping up from 25°C to 95°C, at 1°C min^{-1}. The data were fitted to the Boltzmann equation. The melting temperature (Tm) was calculated by determining the maximum value of the first derivative of the fluorescence transition.

Fluorescence polarisation DNA binding assays

Reactions (volume 20 µl) were carried out in black 384 well plates (Corning, USA). CDIN1, Codanin-1 and CDIN1-Codanin-1 protein were serially diluted from 5 µM to 0.288 nM and incubated with a 19-mer dsDNA substrate (Top strand: 5' ACTCGTCTCTAGCTTTTTT-FITC 3'; Bottom strand: 5' AAAAAAGCTAGAGACGATGT 3' at 25 nM final concentrations in a buffer containing 20 mM HEPES, pH 7.5, 50 mM KCl, 0.5 mM TCEP at room temperature for 15 min. Fluorescence polarisation was measured with excitation at 485 nm and emission at 520 nm using a PHERAstar FS plate reader (BMG Labtech). Fluorescence polarisation was plotted against $\log_{10}$ protein concentration, and binding curves were fitted using a non-linear regression (curve fit) in GraphPad Prism (GraphPad Software Inc., La Jolla, CA, USA).

References

1. Florens, L, M Carozza, S Swanson, M Fournier, M Coleman, J Workman, and M Washburn. 2006. 'Analyzing Chromatin Remodeling Complexes Using Shotgun Proteomics and Normalized Spectral Abundance Factors'. *Methods* 40 (4): 303–11. https://doi.org/10.1016/j.ymeth.2006.07.028.
2. Hughes, Christopher S., Sophie Moggridge, Torsten Müller, Poul H. Sorensen, Gregg B. Morin, and Jeroen Krijgsveld. 2019. 'Single-Pot, Solid-Phase-Enhanced Sample Preparation for Proteomics Experiments'. *Nature Protocols* 14 (1): 68–85. https://doi.org/10.1038/s41596-018-0082-x.
3. Moir-Meyer, Gemma, Pak Leng Cheong, Aude-Anais Olijnik, Jill Brown, Samantha Knight, Andrew King, Ryo Kurita, et al. 2018. 'Robust CRISPR/Cas9 Genome Editing of the HUDEP-2 Erythroid Precursor Line Using Plasmids and Single-Stranded Oligonucleotide Donors'. *Methods and Protocols* 1 (3): 28. https://doi.org/10.3390/mps1030028.
4. Pavelka, Norman, Marjorie L. Fournier, Selene K. Swanson, Mattia Pelizzola, Paola Ricciardi-Castagnoli, Laurence Florens, and Michael P. Washburn. 2008. 'Statistical Similarities between Transcriptomics and Quantitative Shotgun Proteomics Data'. *Molecular & Cellular Proteomics* 7 (4): 631–44. https://doi.org/10.1074/mcp.M700240-MCP200.

Suppl. Fig. 1

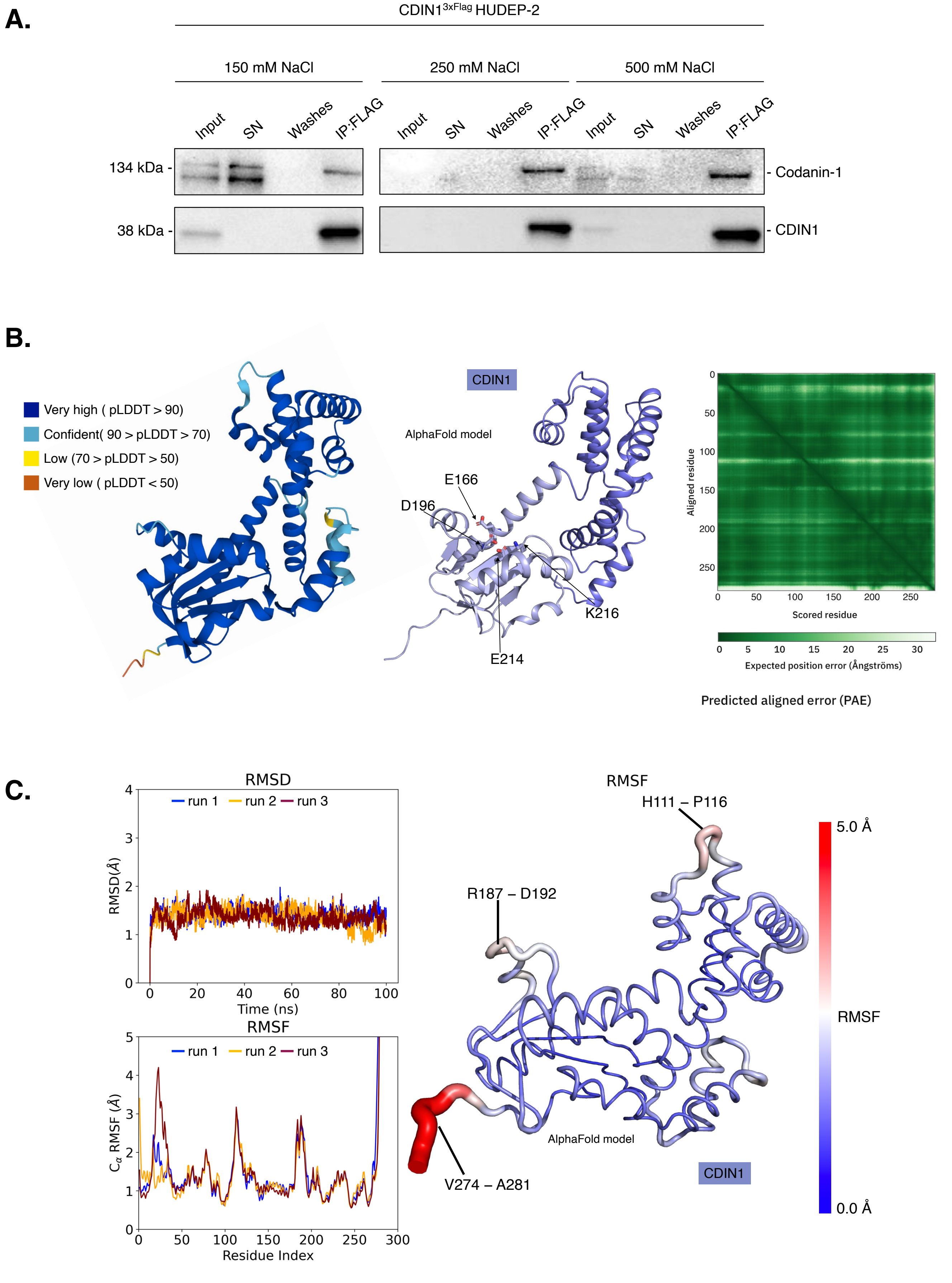
A.
CDIN1 3xFlag HUDEP-2
150 mM NaCl
250 mM NaCl
500 mM NaCl
Input
SN
Washes
IP:FLAG
134 kDa -
38 kDa -
- Codanin-1
- CDIN1
B.
Very high (pLDDT > 90)
Confident(90 > pLDDT > 70)
Low (70 > pLDDT > 50)
Very low (pLDDT < 50)
CDIN1
AlphaFold model
E166
D196
K216
E214
Aligned residue
Scored residue
Expected position error (Ångströms)
Predicted aligned error (PAE)
C.
RMSD
run 1
run 2
run 3
RMSD(Å)
Time (ns)
RMSF
Cα RMSF (Å)
Residue Index
H111 – P116
R187 – D192
V274 – A281
AlphaFold model
CDIN1
5.0 Å
RMSF
0.0 Å

Suppl. Fig. 2

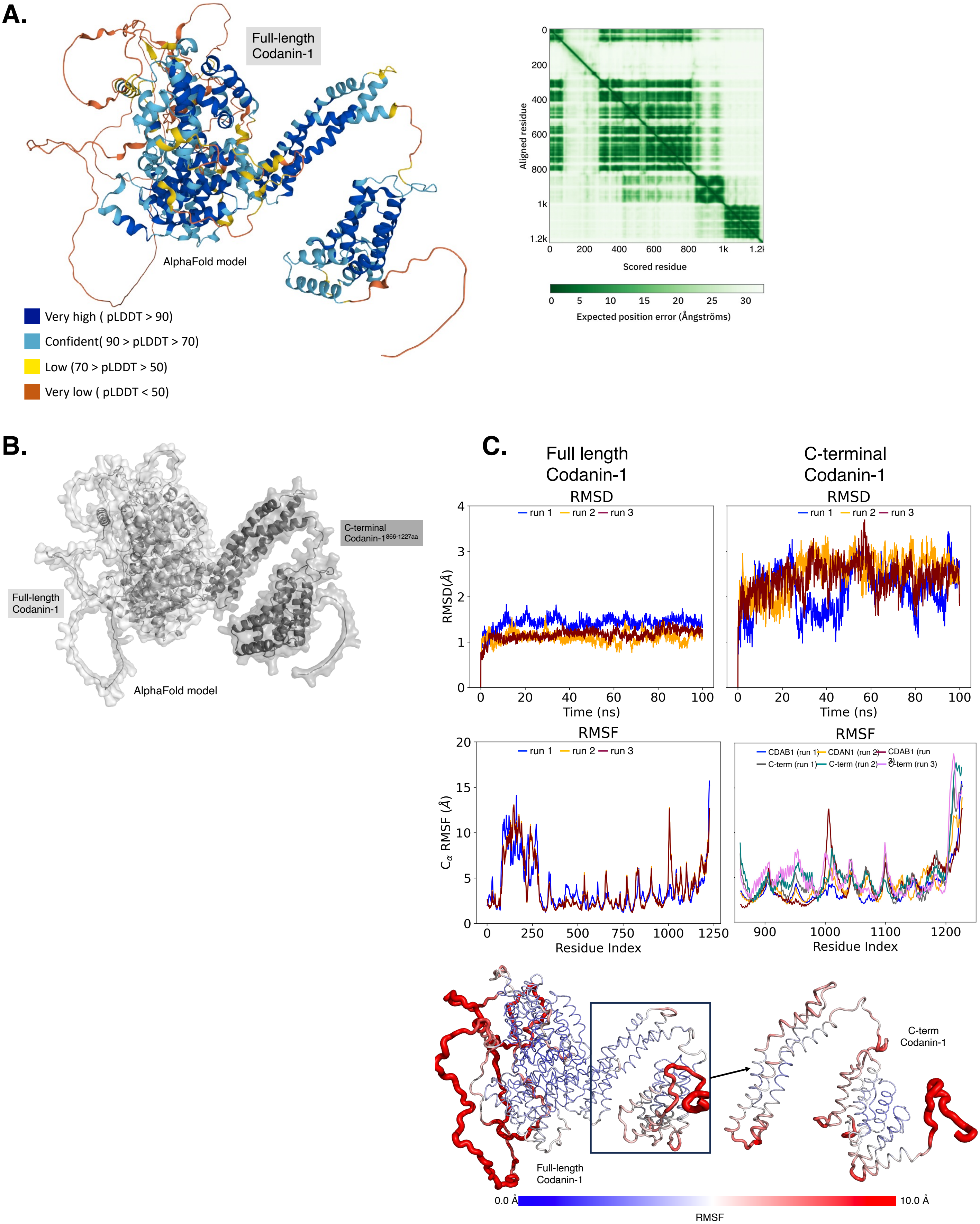
A.
Full-length Codanin-1
AlphaFold model
Very high (pLDDT > 90)
Confident(90 > pLDDT > 70)
Low (70 > pLDDT > 50)
Very low (pLDDT < 50)
Aligned residue
Scored residue
Expected position error (Ångströms)
B.
C-terminal Codanin-1866-1227aa
Full-length Codanin-1
AlphaFold model
C.
Full length Codanin-1
C-terminal Codanin-1
RMSD
run 1 run 2 run 3
RMSD(Å)
Time (ns)
RMSF
Cα RMSF (Å)
Residue Index
CDAB1 (run 1) CDAN1 (run 2) CDAB1 (run 3)
C-term (run 1) C-term (run 2) C-term (run 3)
C-term Codanin-1
Full-length Codanin-1
0.0 Å
10.0 Å
RMSF

Suppl. Fig. 3

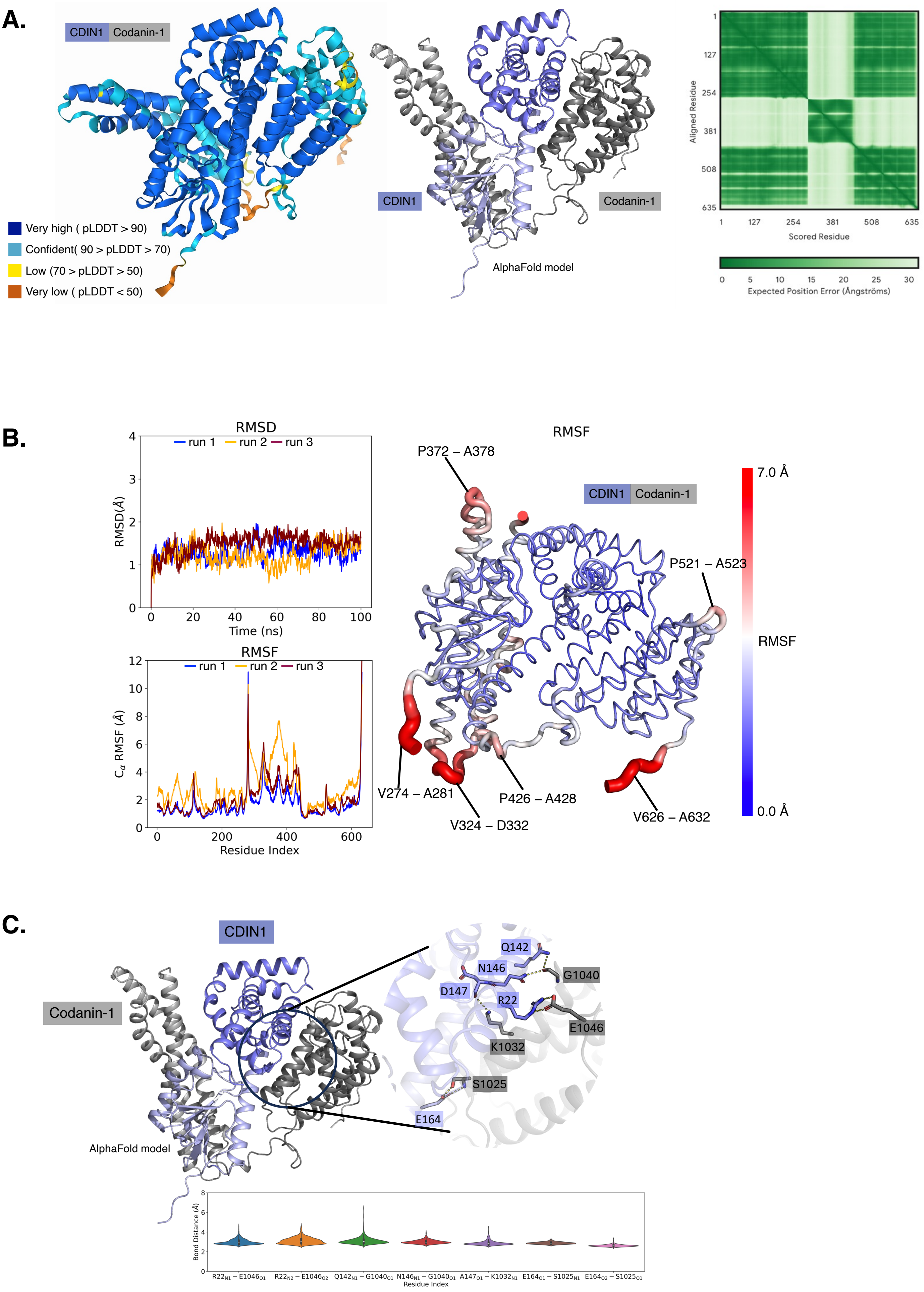

A.
CDIN1
Codanin-1
Very high (pLDDT > 90)
Confident(90 > pLDDT > 70)
Low (70 > pLDDT > 50)
Very low (pLDDT < 50)
CDIN1
Codanin-1
AlphaFold model
Aligned Residue
Scored Residue
Expected Position Error (Ångströms)
B.
RMSD
run 1
run 2
run 3
RMSD(Å)
Time (ns)
RMSF
Cα RMSF (Å)
Residue Index
RMSF
P372 – A378
CDIN1
Codanin-1
P521 – A523
7.0 Å
RMSF
0.0 Å
V274 – A281
P426 – A428
V324 – D332
V626 – A632
C.
CDIN1
Codanin-1
AlphaFold model
Q142
N146
G1040
D147
R22
E1046
K1032
S1025
E164
Bond Distance (Å)
Residue Index

Suppl. Fig. 4

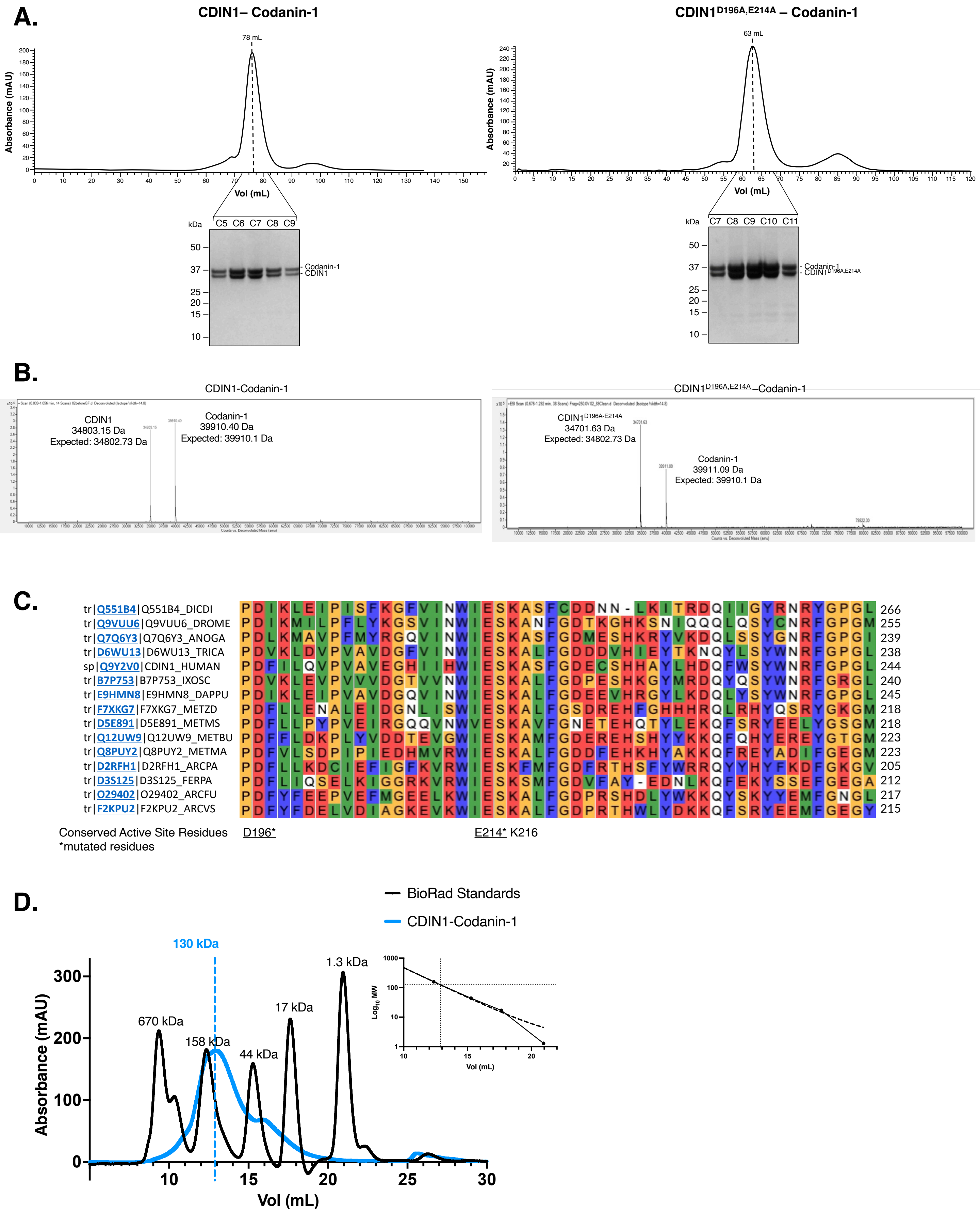
A.
CDIN1– Codanin-1
78 mL
Absorbance (mAU)
Vol (mL)
kDa
C5 C6 C7 C8 C9
50
37
25
20
15
10
Codanin-1
CDIN1
CDIN1^{D196A,E214A} – Codanin-1
63 mL
C7 C8 C9 C10 C11
CDIN1^{D196A,E214A}
B.
CDIN1-Codanin-1
CDIN1
34803.15 Da
Expected: 34802.73 Da
Codanin-1
39910.40 Da
Expected: 39910.1 Da
CDIN1^{D196A,E214A} –Codanin-1
CDIN1$^{D196A-E214A}$
34701.63 Da
Expected: 34802.73 Da
Codanin-1
39911.09 Da
Expected: 39910.1 Da
C.
tr|Q551B4|Q551B4_DICDI PDIKLEIPISFKGFVINWIESKASFCDDNN-LKITRDQIIGYRNRYGPGL 266
tr|Q9VUU6|Q9VUU6_DROME PDIKMILPFLYKGSVINWIESKANFGDTKGHKSNIQQQLQSYCNRFGPGM 255
tr|Q7Q6Y3|Q7Q6Y3_ANOGA PDLKMAVPFMYRGQVINWIESKASFGDMESHKRYVKDQLSSYGNRFGPGI 239
tr|D6WU13|D6WU13_TRICA PDVKLDVPVAVDGFVINWIESKALFGDDDVHIEYTKNQYLSYWNRFGPGL 238
sp|Q9Y2V0|CDIN1_HUMAN PDFILQVPVAVEGHIIHWIESKASFGDECSHHAYLHDQFWSYWNRFGPGL 244
tr|B7P753|B7P753_IXOSC PDVKLEVPVVVDGTVVNWIESKALFGDPESHKGYMRDQYQSYWNRFGRGL 240
tr|E9HMN8|E9HMN8_DAPPU PDIKLEIPVAVDGQVINWIESKALFGDEEVHRGYLKDQLYSYWNRFGPGL 245
tr|F7XKG7|F7XKG7_METZD PDFLLENALEIDGNLISWIESKALFGSDREHFGHHRQLRHYQSRYGKGM 218
tr|D5E891|D5E891_METMS PDFLLPYPVEIRGQQVNWVESKAVFGNETEHQTYLEKQFSRYEELYGSGM 218
tr|Q12UW9|Q12UW9_METBU PDFFLDKPLYVDDTEVGWIESKAMFGDEREHSHYYKKQFQHYEREYGTGM 223
tr|Q8PUY2|Q8PUY2_METMA PDFVLSDPIPIEDHMVRWIESKALFGDDFEHKHYAKKQFREYADIFGEGM 223
tr|D2RFH1|D2RFH1_ARCPA PDFLLKDCIEFIGFKVRWIESKFMFGDFRTHSFYWRRQYYHYFKDFGKGV 205
tr|D3S125|D3S125_FERPA PDFLIQSELKIGGRKVRWIESKSMFGDVFAY-EDNLKQFEKYSSEFGEGA 212
tr|O29402|O29402_ARCFU PDFYFEEPVEFMGEELKWIESKALFGDPRSHDLYWKKQYSKYYEMFGNGL 217
tr|F2KPU2|F2KPU2_ARCVS PDFYFDELVDIAGKEVKWIESKALFGDPRTHWLYDKKQFSRYEEMFGEGY 215
Conserved Active Site Residues D196* E214* K216
*mutated residues
D.
BioRad Standards
CDIN1-Codanin-1
130 kDa
670 kDa
158 kDa
44 kDa
17 kDa
1.3 kDa
Absorbance (mAU)
Vol (mL)
Log10 MW

Suppl. Fig. 5

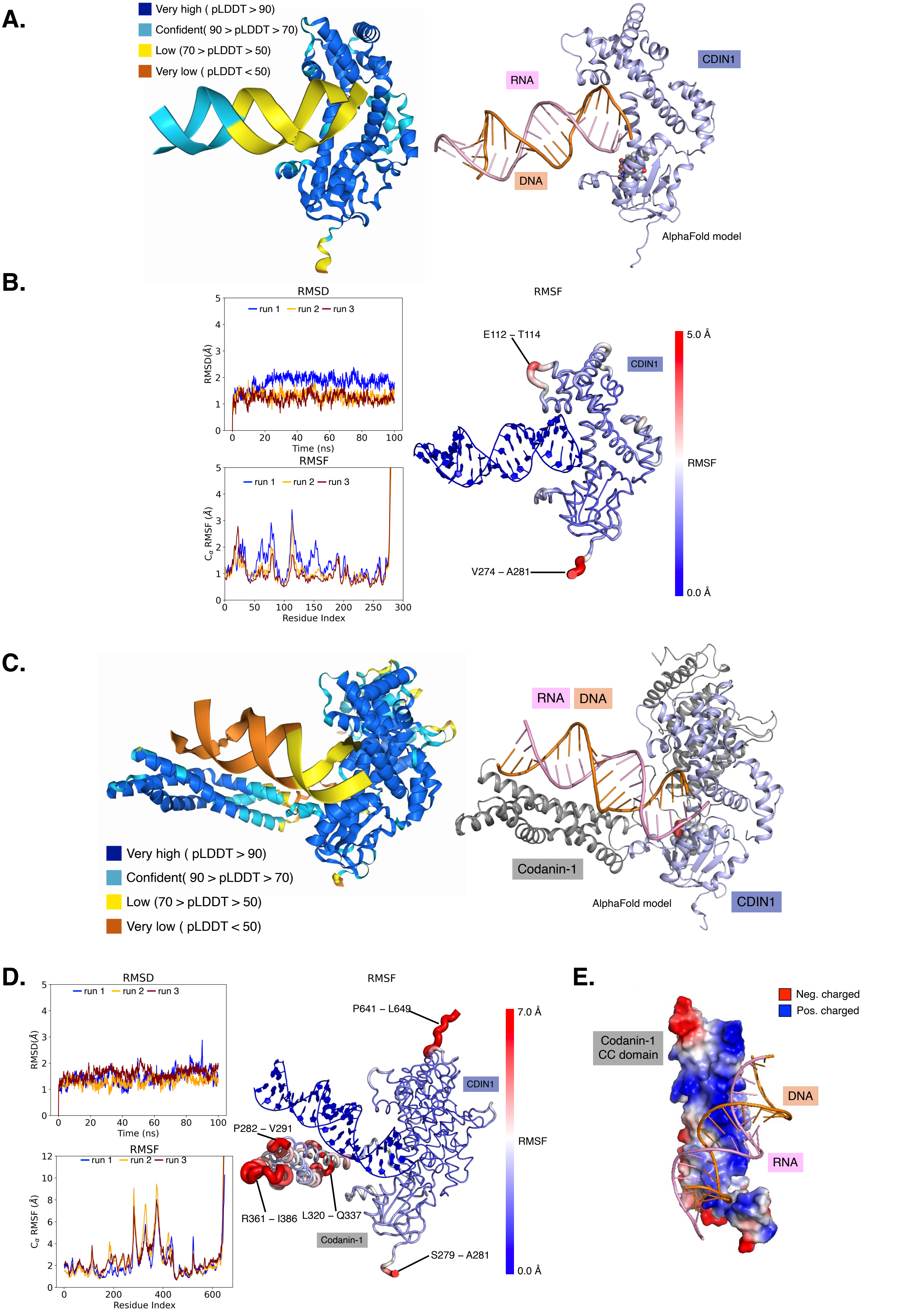

A.
Very high (pLDDT > 90)
Confident(90 > pLDDT > 70)
Low (70 > pLDDT > 50)
Very low (pLDDT < 50)
RNA
DNA
CDIN1
AlphaFold model
B.
RMSD
run 1
run 2
run 3
RMSD(Å)
Time (ns)
RMSF
Cα RMSF (Å)
Residue Index
E112 – T114
CDIN1
V274 – A281
5.0 Å
RMSF
0.0 Å
C.
RNA
DNA
Codanin-1
AlphaFold model
CDIN1
Very high (pLDDT > 90)
Confident(90 > pLDDT > 70)
Low (70 > pLDDT > 50)
Very low (pLDDT < 50)
D.
RMSD
run 1
run 2
run 3
RMSD(Å)
Time (ns)
RMSF
Cα RMSF (Å)
Residue Index
P641 – L649
CDIN1
P282 – V291
R361 – I386
L320 – Q337
Codanin-1
S279 – A281
7.0 Å
RMSF
0.0 Å
E.
Neg. charged
Pos. charged
Codanin-1
CC domain
DNA
RNA

Suppl. Fig. 6

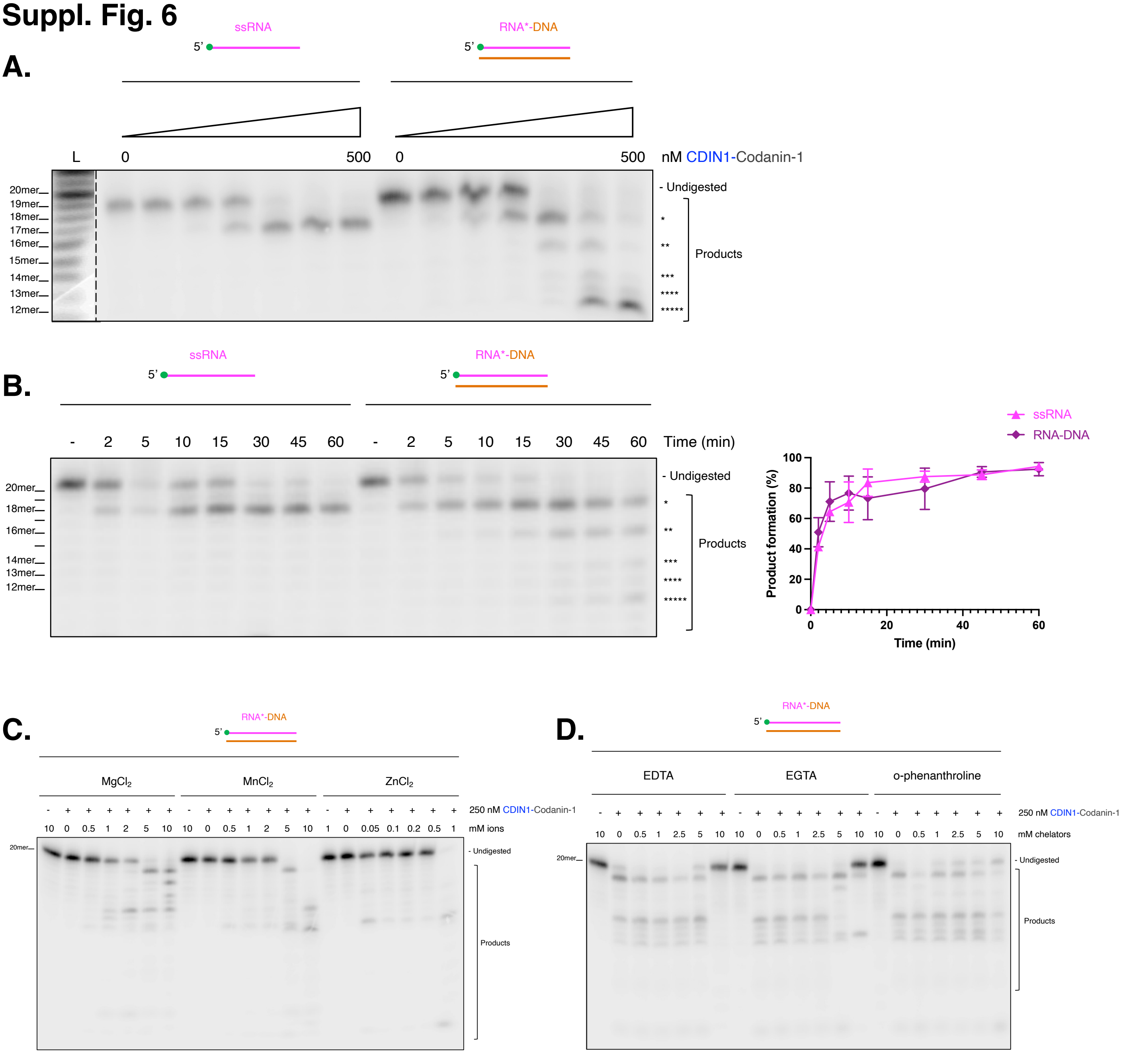

A.
ssRNA
5'
RNA*-DNA
5'
L
0
500
0
500
nM CDIN1-Codanin-1
20mer
19mer
18mer
17mer
16mer
15mer
14mer
13mer
12mer
- Undigested
*
**
***
****
*****
Products
B.
ssRNA
5'
RNA*-DNA
5'
- 2 5 10 15 30 45 60 - 2 5 10 15 30 45 60 Time (min)
20mer
18mer
16mer
14mer
13mer
12mer
- Undigested
*
**
***
****
*****
Products
ssRNA
RNA-DNA
Product formation (%)
100
80
60
40
20
0
0
20
40
60
Time (min)
C.
RNA*-DNA
5'
MgCl2
MnCl2
ZnCl2
- + + + + + + - + + + + + + - + + + + + +
250 nM CDIN1-Codanin-1
10 0 0.5 1 2 5 10 10 0 0.5 1 2 5 10 1 0 0.05 0.1 0.2 0.5 1
mM ions
20mer
- Undigested
Products
D.
RNA*-DNA
5'
EDTA
EGTA
o-phenanthroline
- + + + + + + - + + + + + + - + + + + + +
250 nM CDIN1-Codanin-1
10 0 0.5 1 2.5 5 10 10 0 0.5 1 2.5 5 10 10 0 0.5 1 2.5 5 10
mM chelators
20mer
- Undigested
Products

Suppl. Fig. 7

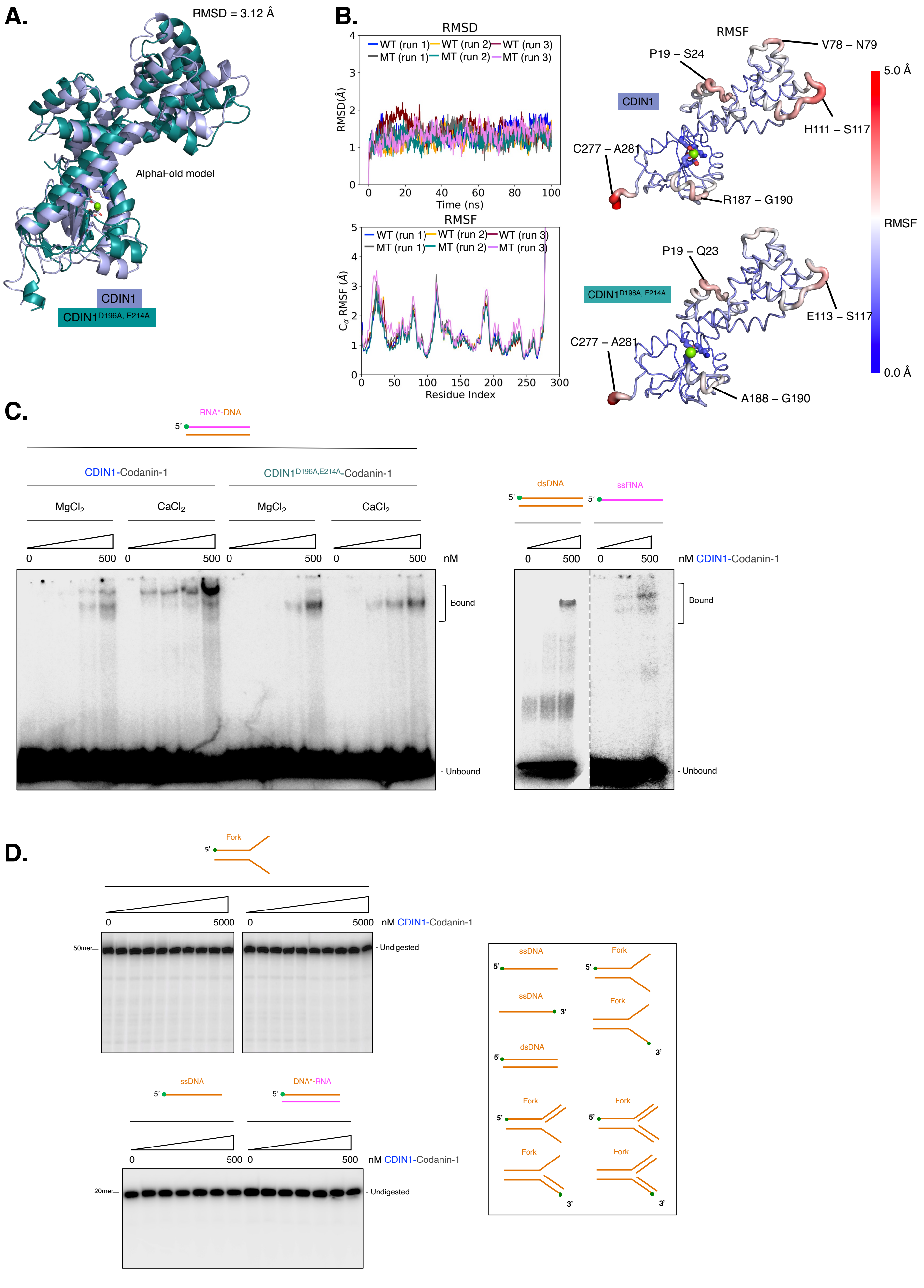
A.
RMSD = 3.12 Å
AlphaFold model
CDIN1
CDIN1D196A, E214A
B.
RMSD
WT (run 1)
WT (run 2)
WT (run 3)
MT (run 1)
MT (run 2)
MT (run 3)
RMSD(Å)
Time (ns)
RMSF
Cα RMSF (Å)
Residue Index
RMSF
V78 – N79
P19 – S24
CDIN1
H111 – S117
C277 – A281
R187 – G190
5.0 Å
RMSF
0.0 Å
P19 – Q23
CDIN1D196A, E214A
E113 – S117
C277 – A281
A188 – G190
C.
RNA*-DNA
5'
CDIN1-Codanin-1
CDIN1D196A,E214A-Codanin-1
MgCl2
CaCl2
MgCl2
CaCl2
0
500
nM
Bound
- Unbound
dsDNA
ssRNA
nM CDIN1-Codanin-1
D.
Fork
0
5000
nM CDIN1-Codanin-1
50mer
- Undigested
ssDNA
DNA*-RNA
0
500
nM CDIN1-Codanin-1
20mer
- Undigested
ssDNA
Fork
ssDNA
Fork
3'
dsDNA
Fork
Fork
Fork
Fork

Suppl. Fig. 8

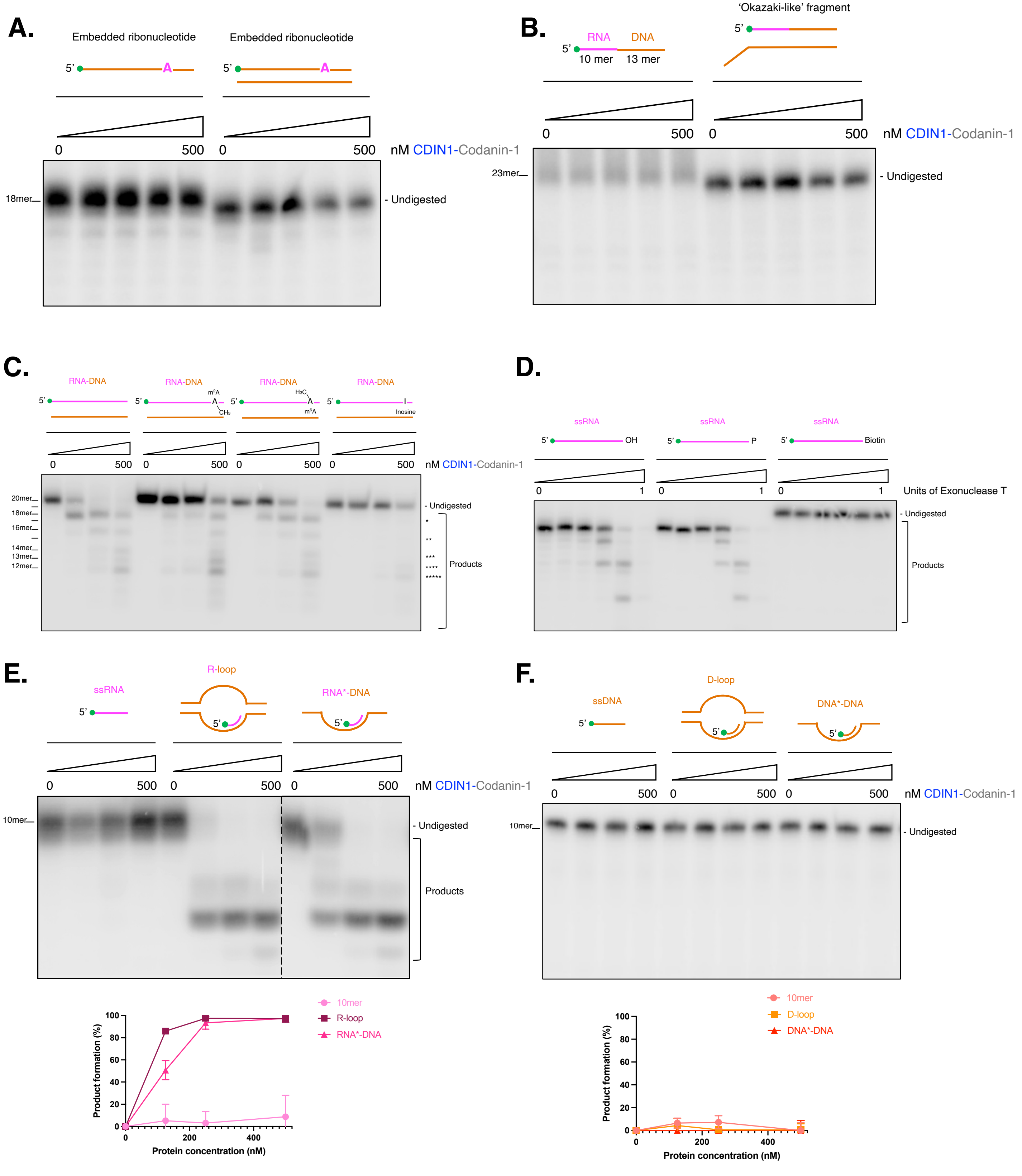
A.
Embedded ribonucleotide
Embedded ribonucleotide
5'
A
0
500
nM CDIN1-Codanin-1
18mer
- Undigested
B.
RNA
DNA
10 mer
13 mer
'Okazaki-like' fragment
23mer
C.
RNA-DNA
m²A
H₃C
m⁶A
CH₃
Inosine
20mer
18mer
16mer
14mer
13mer
12mer
Products
D.
ssRNA
OH
P
Biotin
Units of Exonuclease T
0
1
E.
ssRNA
R-loop
RNA*-DNA
10mer
Product formation (%)
Protein concentration (nM)
R-loop
F.
ssDNA
D-loop
DNA*-DNA
D-loop
100
80
60
40
20
200
400

Suppl. Fig. 9

A.

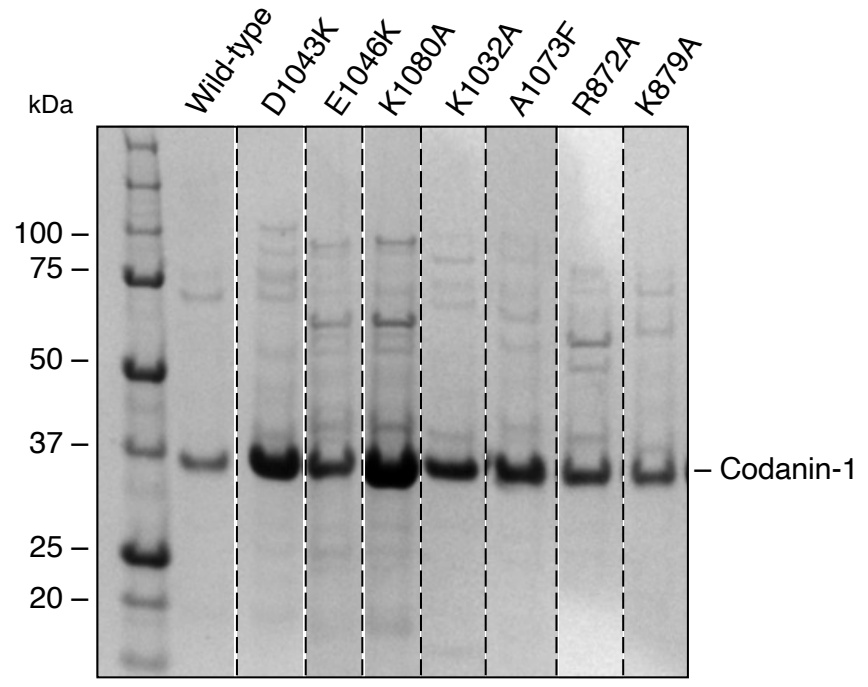

B.

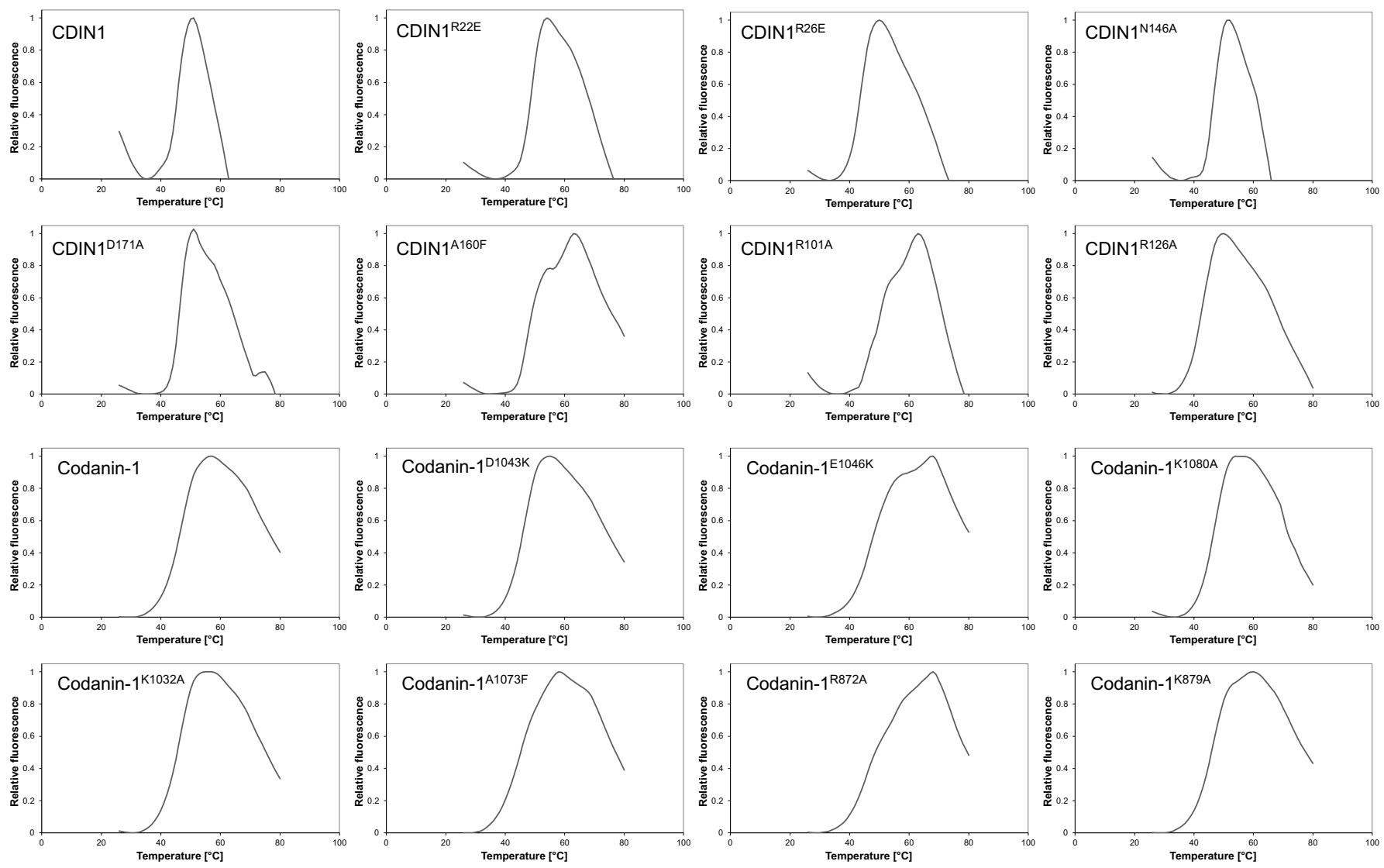

	ΔTm(°C ± SD)
CDIN1	45 ± 0.18
CDIN1^{R22E}	48.46 ± 0.06
CDIN1^{R26E}	44.77 ± 0.07
CDIN1^{N146A}	47.10 ± 0.07
CDIN1^{D171A}	45.60 ± 0.07
CDIN1^{A160F}	49.20 ± 0.30
CDIN1^{R101A}	50.04 ± 0.34
CDIN1^{R126A}	44.30 ± 0.08

	ΔTm(°C ± SD)
Codanin-1	46.40 ± 0.08
Codanin-1^{D1043K}	44.86 ± 0.06
Codanin-1^{E1046K}	47.92 ± 0.18
Codanin-1^{K1080A}	45.54 ± 0.07
Codanin-1^{K1032A}	44.75 ± 0.08
Codanin-1^{A1073F}	45.17 ± 0.07
Codanin-1^{R872A}	49.65 ± 0.29
Codanin-1^{K879A}	45.97 ± 0.04

C.

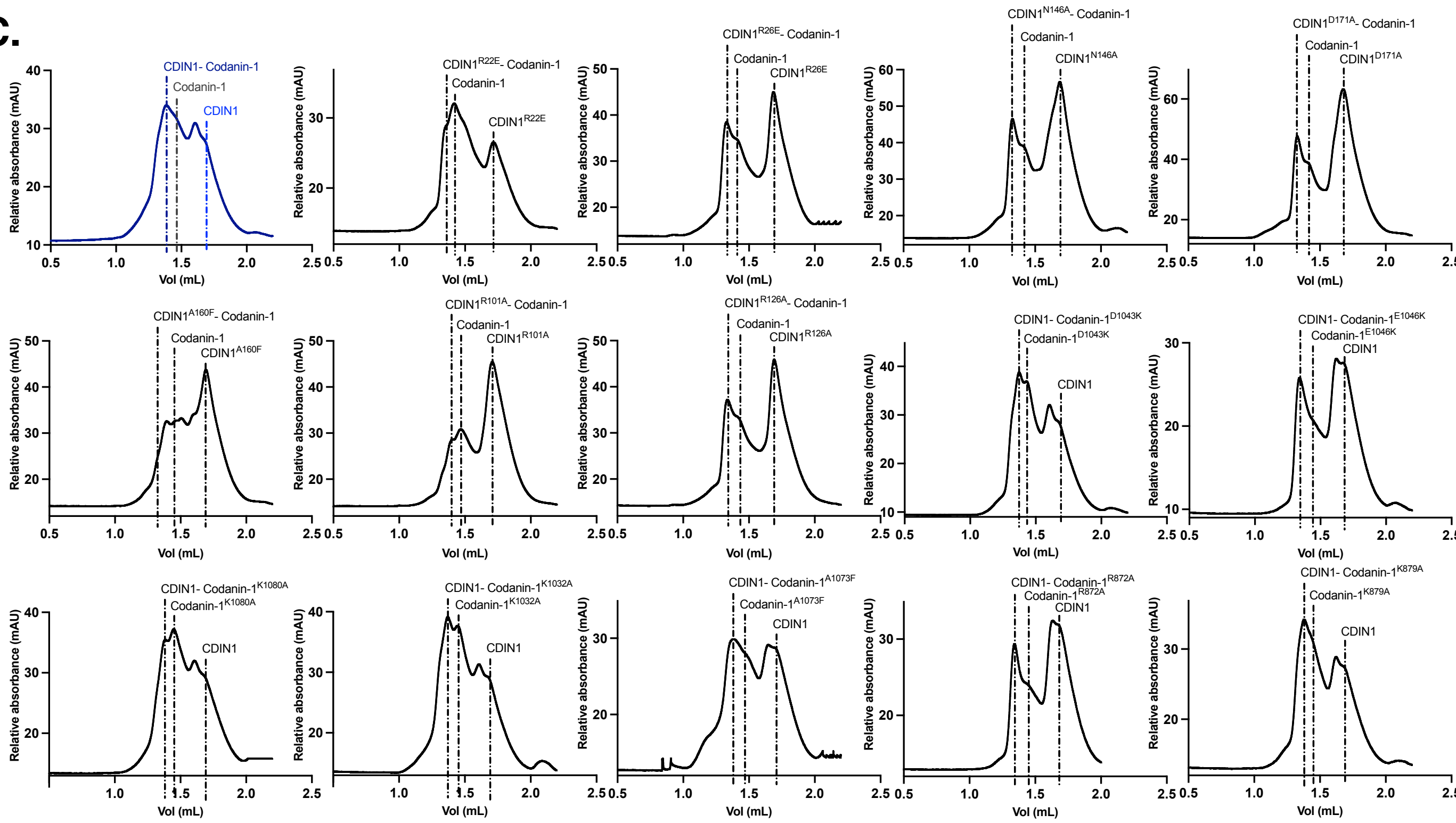

Suppl. Fig. 10

A.

Sample	ka (1/Ms)	kd (1/s)	Kd (M)	Rmax (RU)	Chi² (RU²)	Model
CDIN1	3.84E+04	4.05E-04	1.05E-08	731.2	249	1:1 with Drift
CDIN1^{D171A}	2.78E+04	6.82E-04	2.45E-08	294.2	354	1:1 with Drift
CDIN1^{R22E}	8.01E+03	1.02E-03	1.27E-07	192.5	29.1	1:1 with Drift
CDIN1^{R26E}	1.34E+04	3.52E-03	2.63E-07	108	50.5	1:1 with Drift
CDIN1^{A160F}	4.32E+03	2.24E-03	5.19E-07	261.7	354	1:1 with Drift
CDIN1^{N146A}	1.64E+04	1.95E-02	1.19E-06	170.8	82.4	1:1 with Drift

	Ka (1/Ms)	Kd (1/s)	Kd (M)	Rmax (RU)	Chi² (RU²)	Model
Codanin-1	27700	0.0015	5.42E-08	811.3	1140	1:1 with Drift
Codanin-1^{D1043K}	48400	0.00479	9.91E-08	835.8	1190	1:1 with Drift
Codanin-1^{A1073F}	21700	0.00533	2.46E-07	684.1	253	1:1 with Drift
Codanin-1^{K1080A}	6480	0.0017	2.62E-07	711.4	338	1:1 with Drift
Codanin-1^{K1032A}	5770	0.00152	2.64E-07	524	264	1:1 with Drift
Codanin-1^{E1046K}	3400	0.00126	3.7E-07	623.4	763	1:1 with Drift

B.

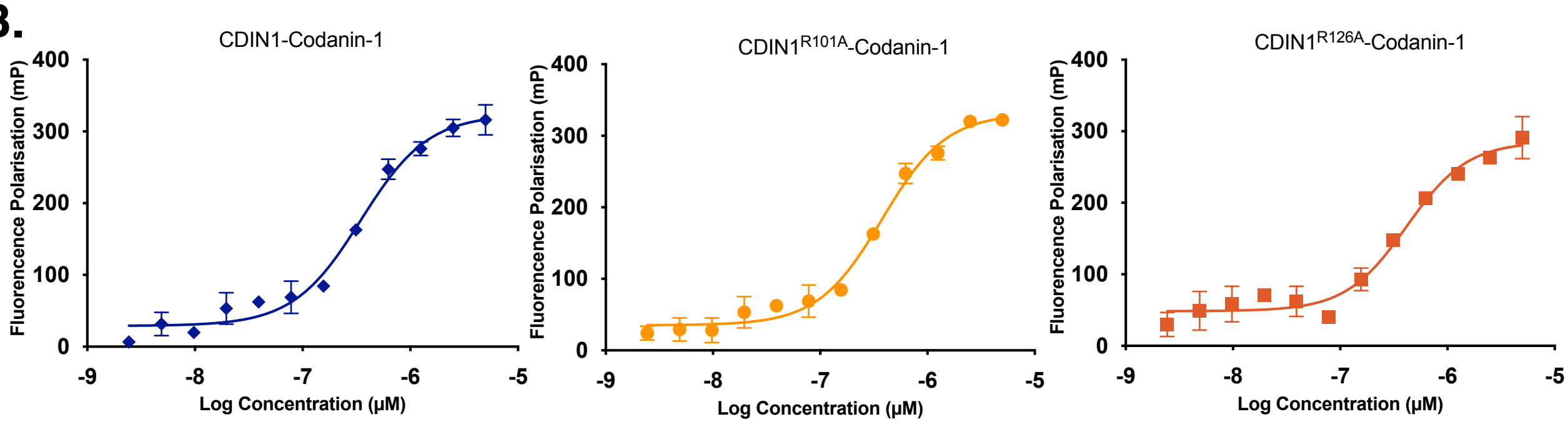

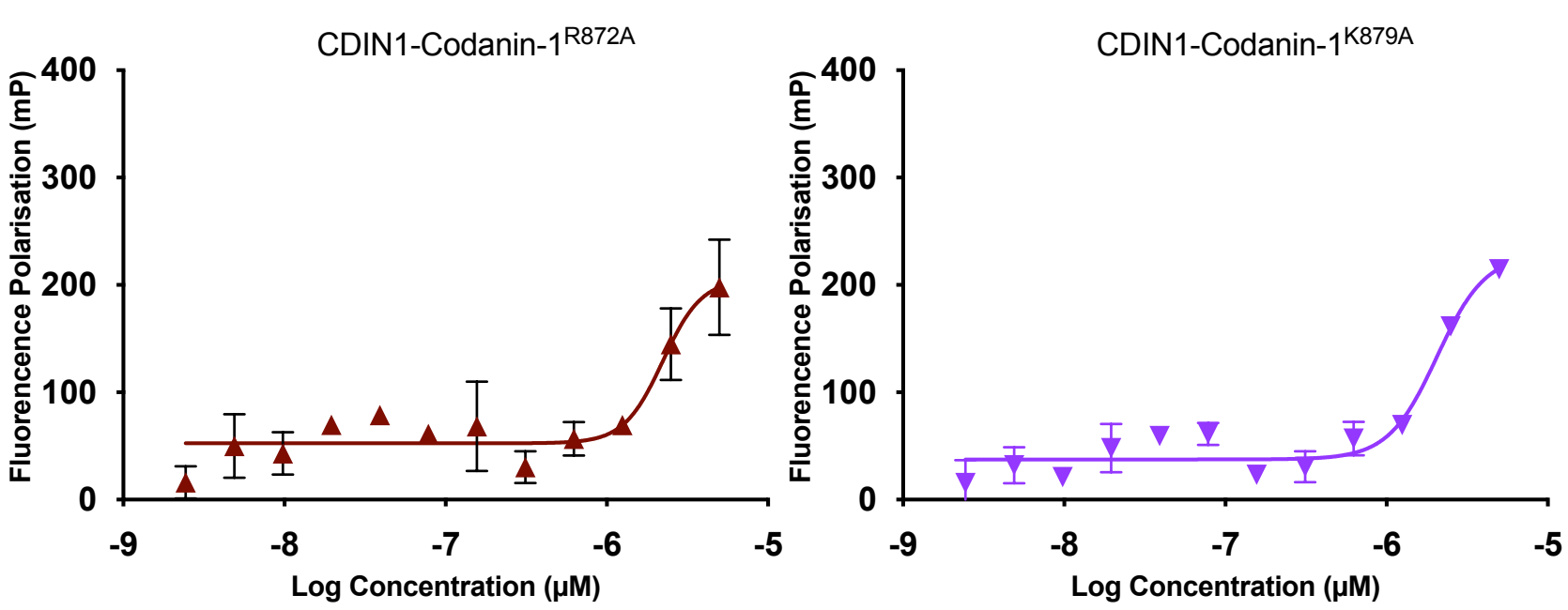

	Kd (µM)	Range (µM)
CDIN1-Codanin-1	0.347	0.287 to 0.433
CDIN1^{R101A}-Codanin-1	0.376	0.320 to 0.453
CDIN1^{R126A}-Codanin-1	0.416	0.324 to 0.580
CDIN1-Codanin-1^{R872A}	2.22	1.57 to not determined
CDIN1-Codanin-1^{K879A}	2.05	1.65 to 3.65

C.

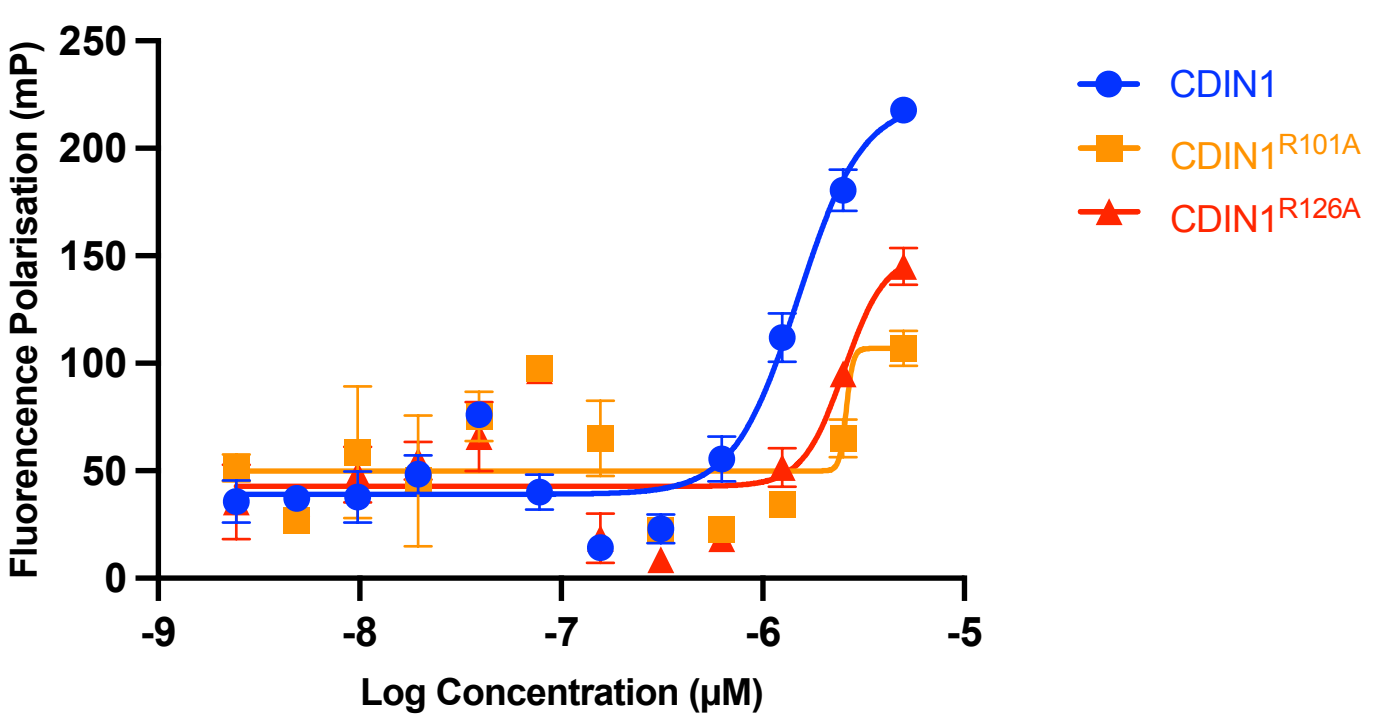

	Kd (µM)	Range (µM)
CDIN1	1.50	1.22 to 1.99
CDIN1^{R101A}	2.59	Not determined
CDIN1^{R126A}	2.53	1.64 to not determined

Suppl. Fig. 11

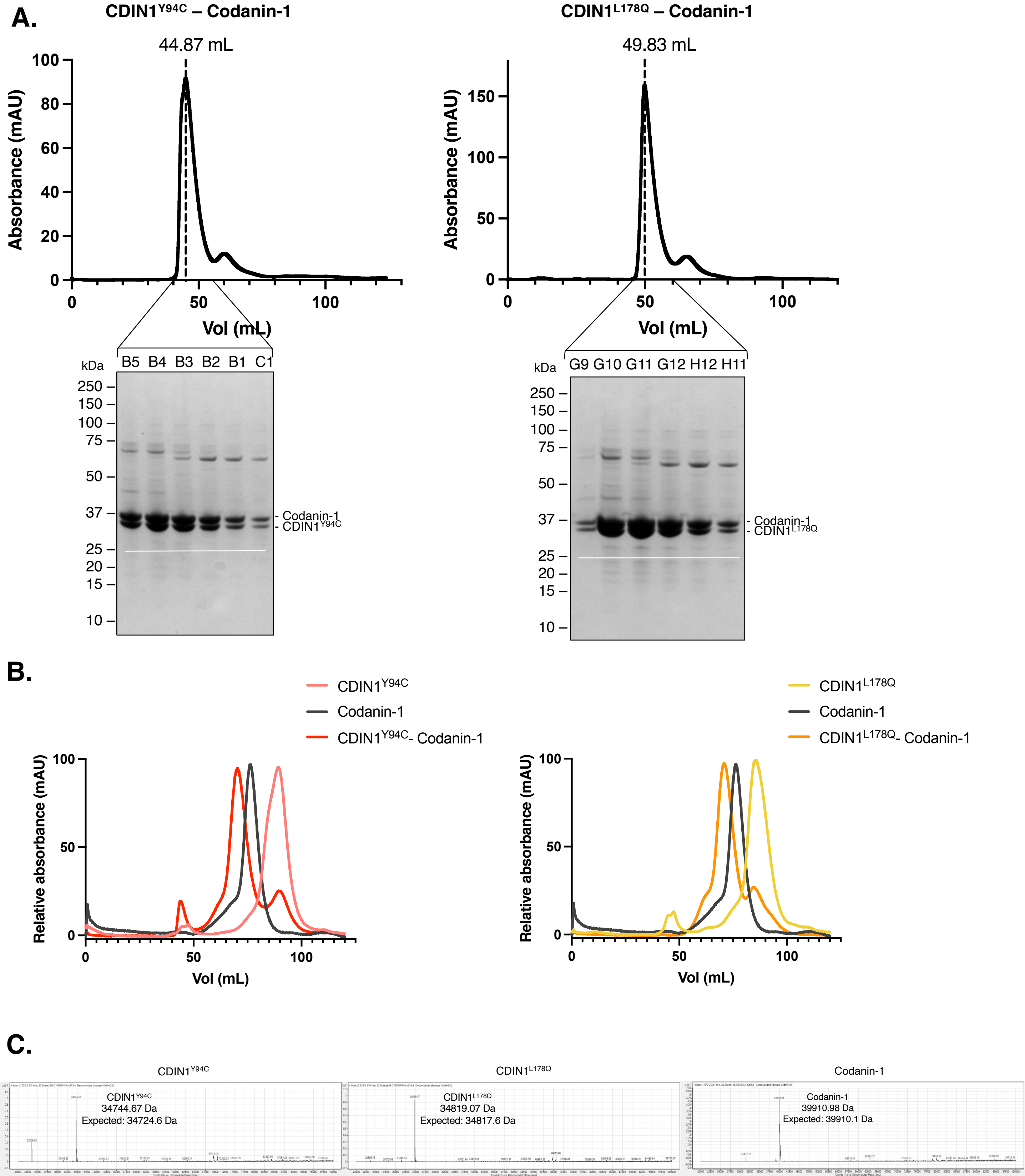
A.
CDIN1Y94C – Codanin-1
44.87 mL
Absorbance (mAU)
Vol (mL)
kDa
B5 B4 B3 B2 B1 C1
250 150 100 75 50 37 25 20 15 10
Codanin-1
CDIN1Y94C
CDIN1L178Q – Codanin-1
49.83 mL
G9 G10 G11 G12 H12 H11
CDIN1L178Q
B.
CDIN1Y94C
Codanin-1
CDIN1Y94C- Codanin-1
CDIN1L178Q
CDIN1L178Q- Codanin-1
Relative absorbance (mAU)
Vol (mL)
C.
CDIN1Y94C
34744.67 Da
Expected: 34724.6 Da
CDIN1L178Q
34819.07 Da
Expected: 34817.6 Da
Codanin-1
39910.98 Da
Expected: 39910.1 Da

Suppl. Fig. 12

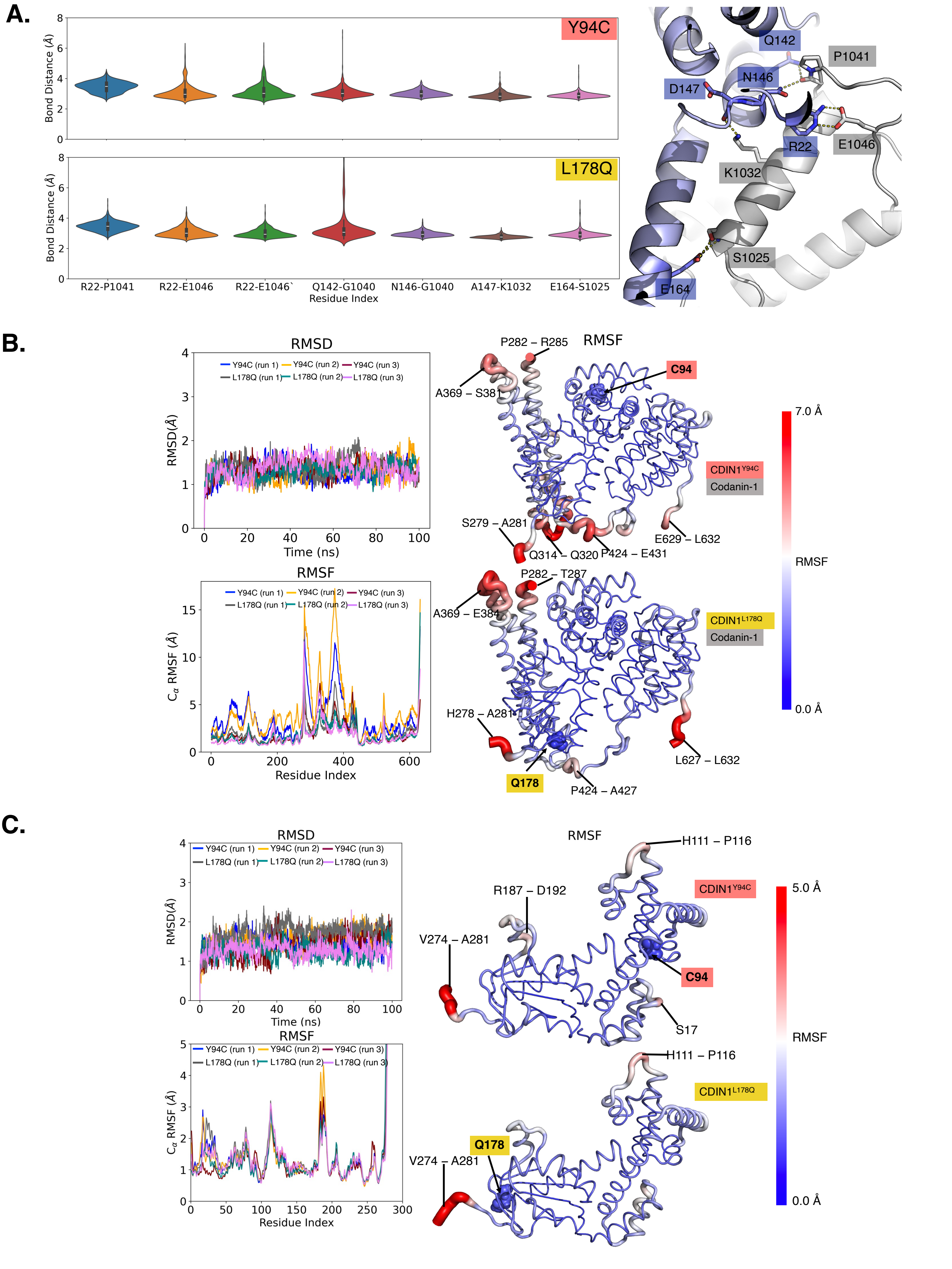
A.
Y94C
L178Q
Bond Distance (Å)
R22-P1041
R22-E1046
R22-E1046`
Q142-G1040
N146-G1040
A147-K1032
E164-S1025
Residue Index
Q142
P1041
N146
D147
R22
E1046
K1032
S1025
E164
B.
RMSD
Y94C (run 1)
Y94C (run 2)
Y94C (run 3)
L178Q (run 1)
L178Q (run 2)
L178Q (run 3)
RMSD(Å)
Time (ns)
RMSF
Cα RMSF (Å)
Residue Index
P282 – R285
A369 – S381
C94
CDIN1Y94C
Codanin-1
S279 – A281
Q314 – Q320
P424 – E431
E629 – L632
P282 – T287
A369 – E384
CDIN1L178Q
H278 – A281
Q178
P424 – A427
L627 – L632
7.0 Å
0.0 Å
C.
H111 – P116
R187 – D192
V274 – A281
S17
5.0 Å

Suppl. Fig. 13

A.

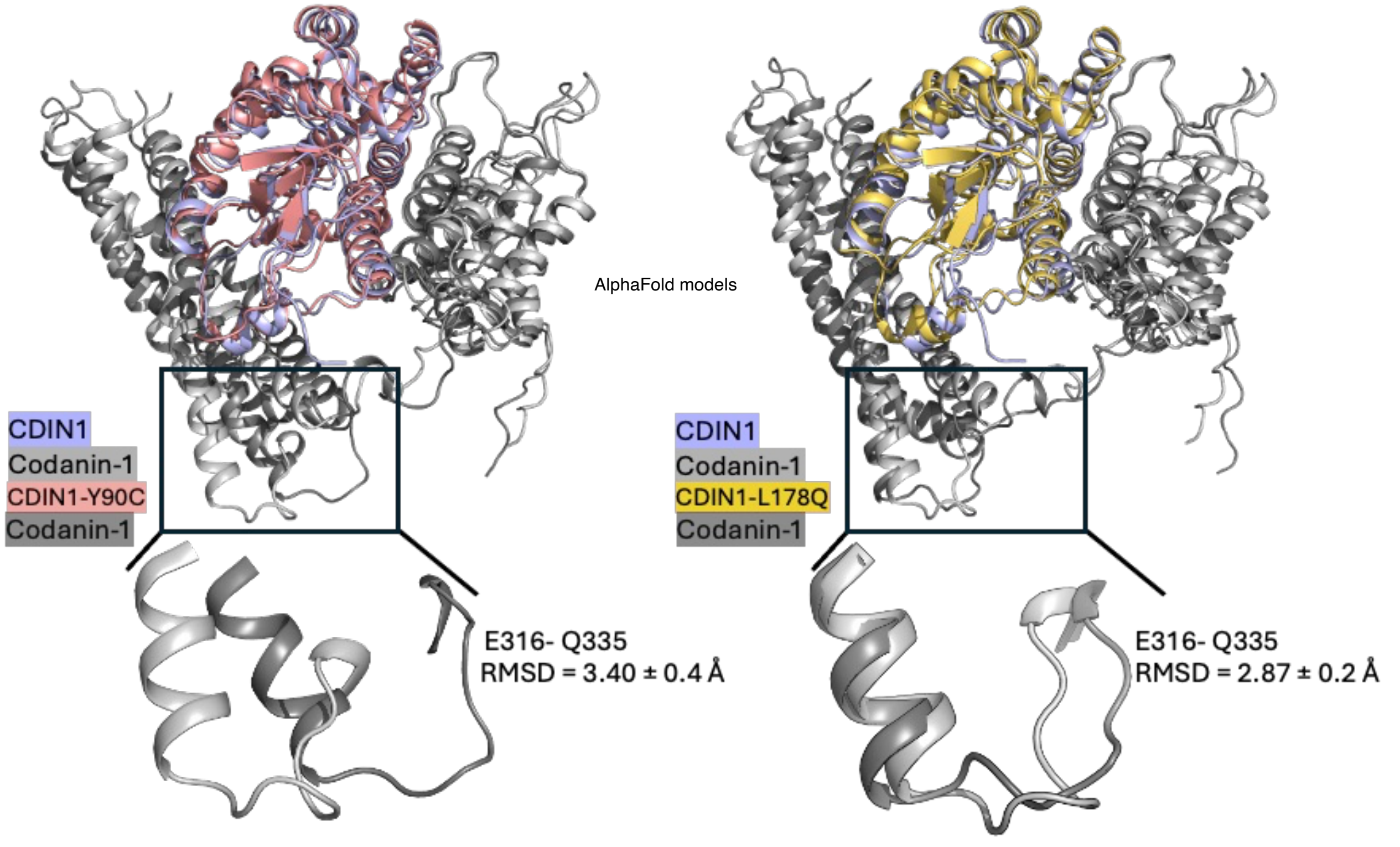

B.

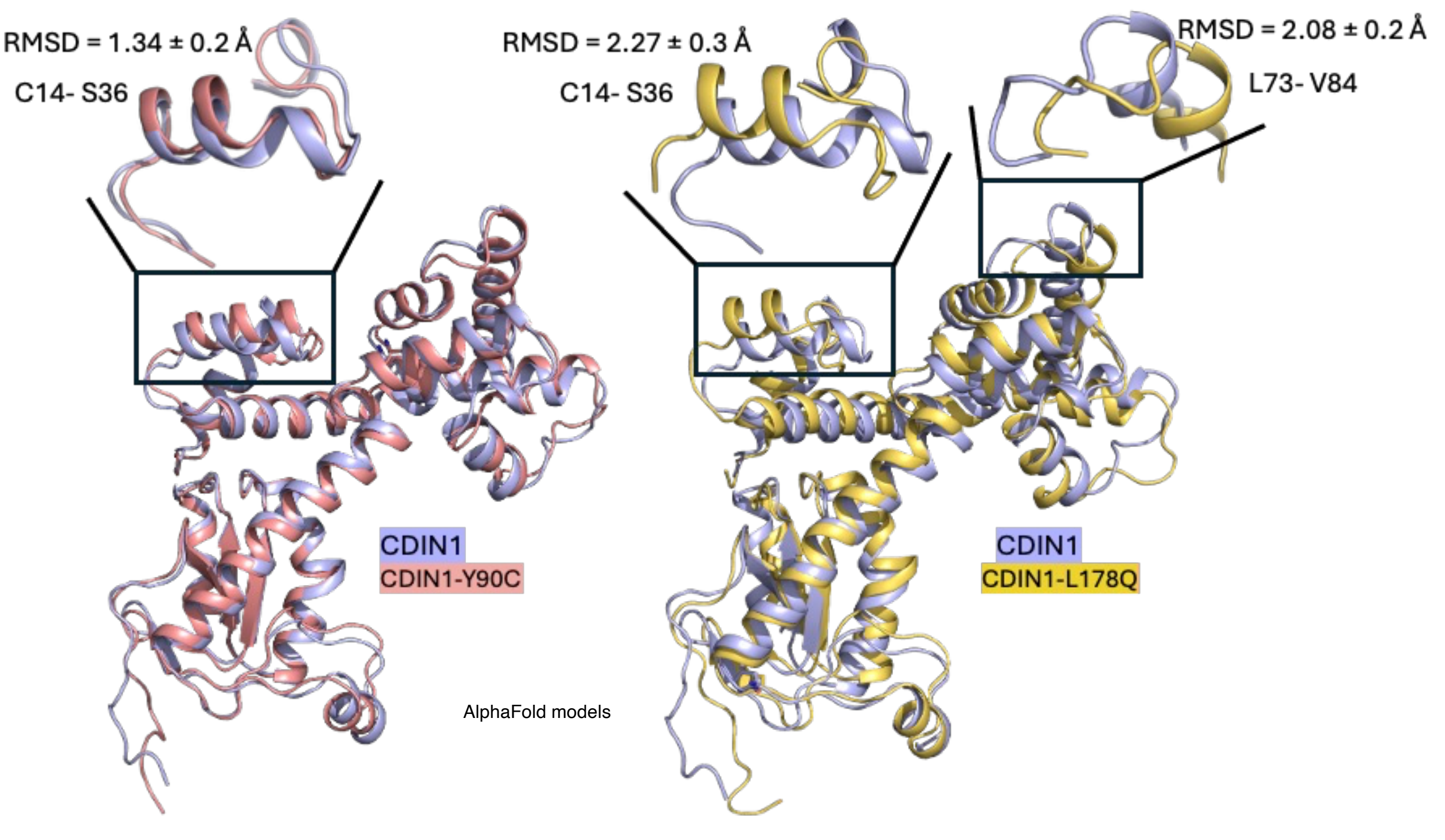

Suppl. Fig. 14

A.

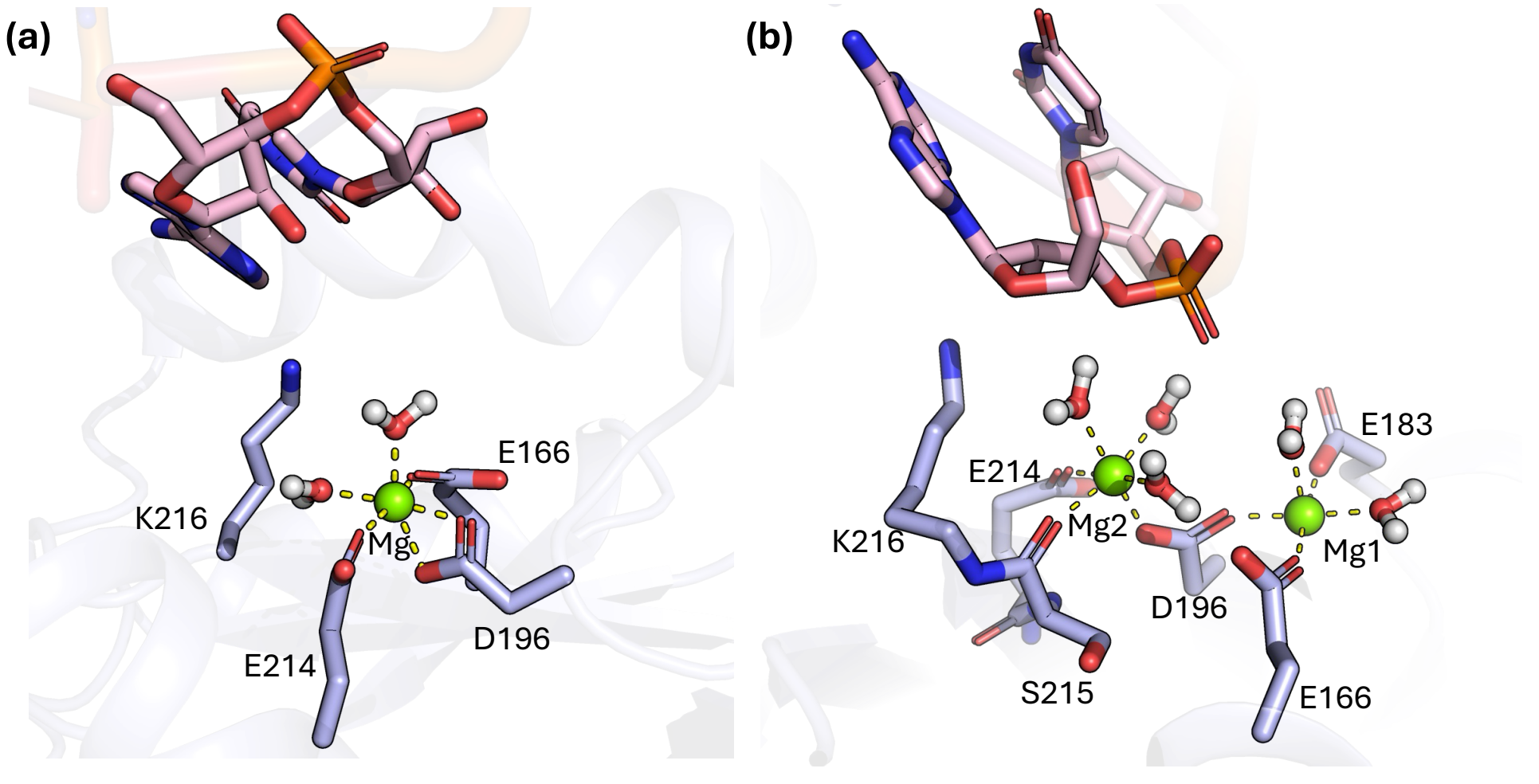

AlphaFold models

B.

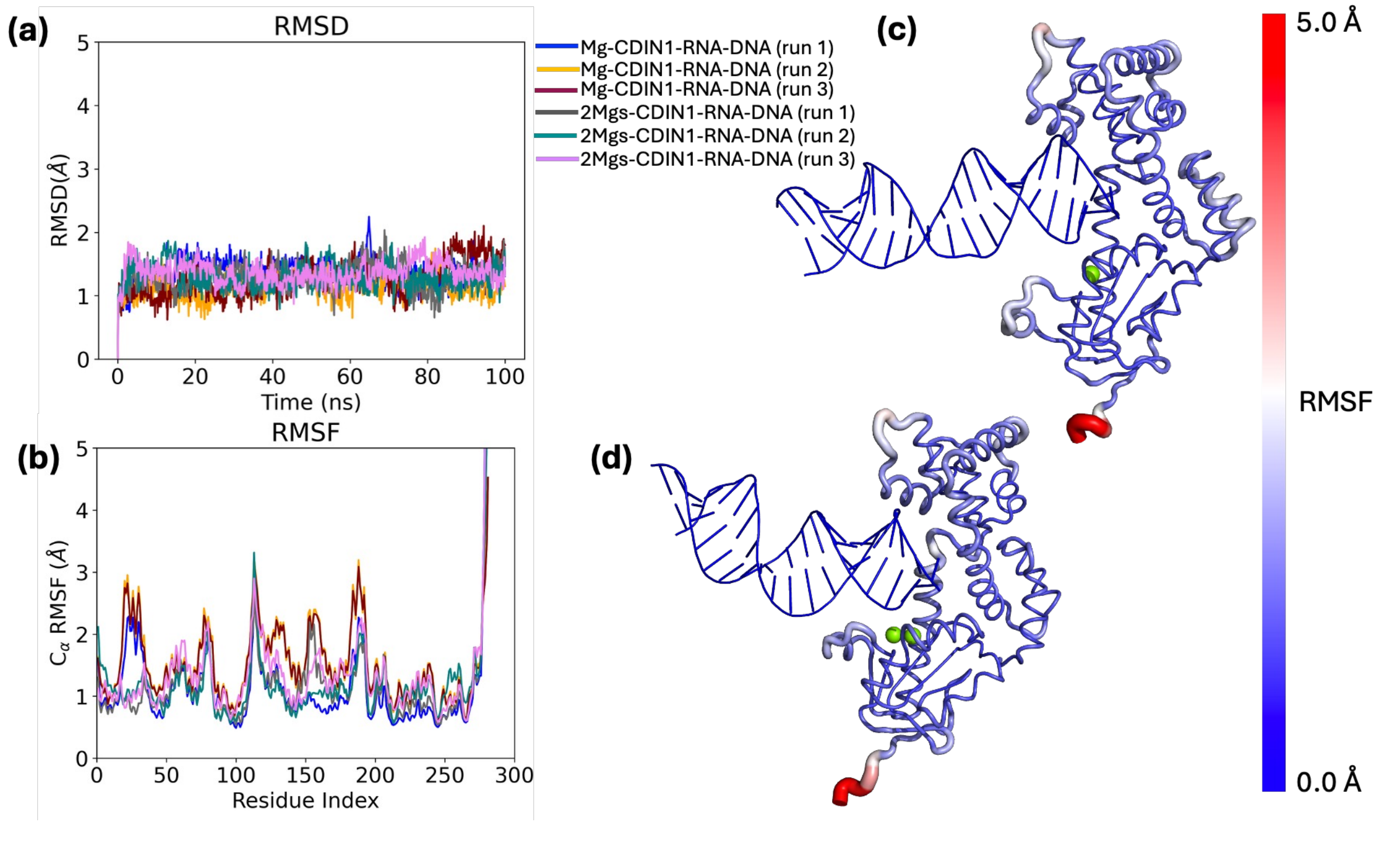

Suppl. Fig. 15

A.

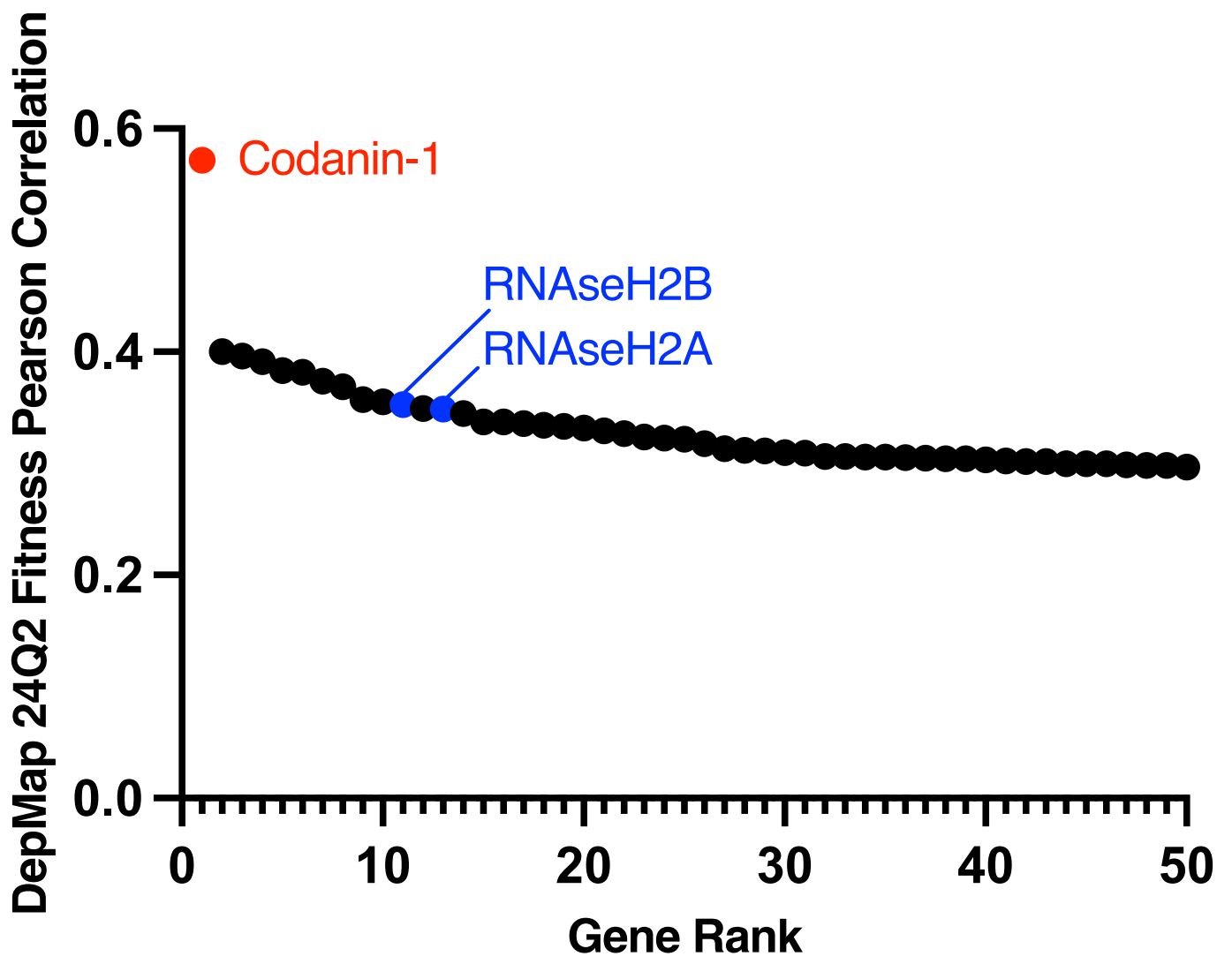

Suppl. Fig. 16

A.

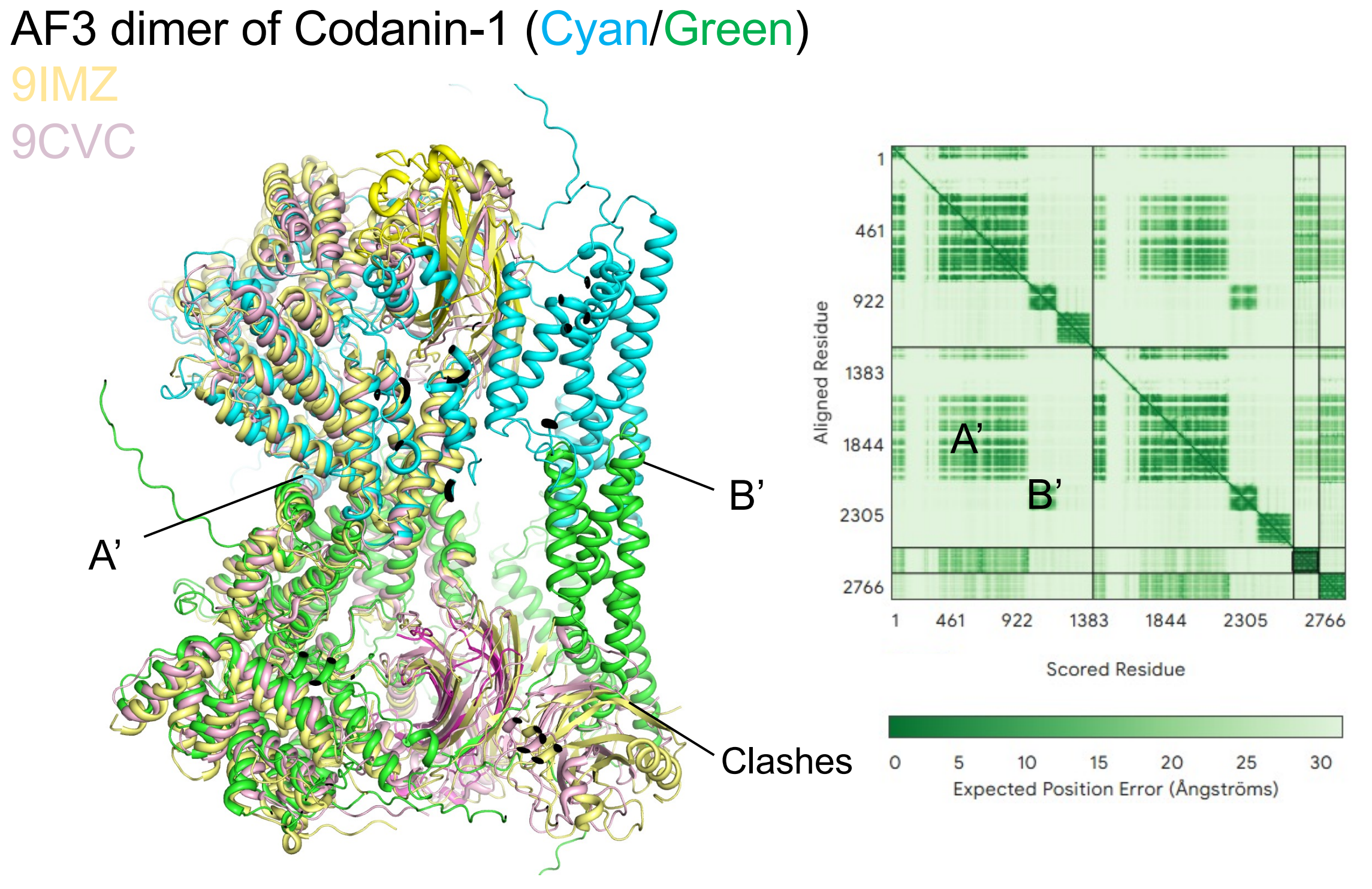

Suppl. Table 1

A.

Name	Description	Sequence (5' – 3')
1	10 mer RNA	AUA AUU UGA U
2	10 mer DNA	ATC AAA TTA T
3	20 mer RNA	AUA AUU UGA UCA UCU AUU AU
4	20 mer RNA	AUA AUA GAU GAU CAA AUU AU
5	20 mer RNA with 3'-Phosphate	AUA AUU UGA UCA UCU AUU AU [PHO]
6	20 mer RNA with 3'-Biotin	AUA AUU UGA UCA UCU AUU AU [BIOTEG]
7	20 mer DNA	ATA ATA GAT GAT CAA ATT AT
8	20 mer DNA	ATA ATT TGA TCA TCT ATT AT
9	30 mer RNA	GUU GAG AGA GAG AGA GUU UGG AGA GUU UGU
10	30 mer DNA	ACA AAC TCT CCA AAC TCT CTC TCT CTC AAC
11	18 mer DNA with a single ribonucleotide at position 15	GAT CTG AGC CTG GG[A] GCT
12	18 mer DNA	AGC TCC CAG GCT CAG ATC
13	10 mer RNA-13 mer DNA	UGC CUG CAG G- TCG ACT CTA GAG G
14	40 mer DNA flap	CC TCT AGA GTC GAC CTG CAG GCA TGC AAG CTT TTG TTC CC
15	50 mer DNA loop top strand	CAA TTA GCT GAG GGT CAC GTT TAA TTA ATT ATT TAC GGA AGA GTA TGA AG
16	50 mer DNA loop bottom strand	TCT TCA CAC TCT TCC GTA GGA CCG ACG GAT TTG GTG ACC CTC AGC TAA TT
17	10 mer RNA for R-loop	CCG UCG GUC C
18	10 mer DNA for D-loop	CCG TCG GTC C
19	20 mer RNA with a 2-methyladenosine at position 19	AUA AUU UGA UCA UCU AUU A*U
20	20 mer RNA with a N6-methyladenosine at position 19	AUA AUU UGA UCA UCU AUU A*U
21	20 mer RNA with an inosine at position 19	AUA AUU UGA UCA UCU AUU I*U
22	DNA fork top strand	ATA AAT ATT TTT TAT TAA TAA TAG ATC A CC TTT CTT TCT CTT CTC CCC TT
23	DNA fork bottom strand	TT CCC CTC CTC TCC TTC CTT CCT GATC TAT TAT TAA TAA AAA ATA TTT AT
24	DNA leading strand	AAG GGG AGA AGA GAA AGA AAG G
25	DNA lagging strand	GGA AGG AAG GAG AGG AGG GGA

B.

Structure	Code
10 mer RNA-DNA hybrid	1 + 2
20 mer RNA-DNA hybrid	3 + 7
30 mer RNA-DNA hybrid	14 + 16
Double-stranded RNA	3 + 4
Double-stranded DNA	7+8
R-loop	15 + 16 + 17
D-loop	15 + 16 +18
Double-stranded RNA with a 2-methyladenosine on the top strand	19 + 4
Double-stranded RNA with a N6-methyladenosine on the top strand	20 + 4
Double-stranded RNA with an inosine on the top strand	21 + 4
Double-stranded RNA with a single ribonucleotide on the top strand	11 + 12
'Okazaki'-like structure	13 + 14
DNA fork	22 + 23
DNA fork with a leading strand	22 + 23 + 24
DNA fork with a lagging strand	22 + 23 + 25
DNA 'replication' fork	22 + 23 + 24 + 25

Suppl. Table 2

MD simulation systems	Simulation replicas X times	Box dimensions (X, Y, Z Å) initial	Box dimensions (X, Y, Z Å) final	Number of atoms	Number of waters	Number of ions (Na+)
**CDIN1**	3 X 100 ns	101.0, 101.0, 101.0	97.5, 97.5, 97.5	71804	22427	6
**CDIN1^{Y94C}**	3 X 100 ns	101.0, 101.0, 101.0	97.5, 97.5, 97.5	71794	22427	6
**CDIN1^{L178Q}**	3 X 100 ns	101.0, 101.0, 101.0	97.6, 97.6, 97.6	71802	22427	6
**Full Length Codanin-1**	3 X 100 ns	162.2, 162.2, 162.2	169.6, 169.6, 169.6	300442	93836	8
**C-terminal Codanin-1**	3 X 100 ns	110.7, 110.7, 110.7	106.8, 106.8, 106.8	18926	29450	6
**Mg-CDIN1**	3 X 100 ns	94.7, 94.7, 94.7	91.4, 91.4, 91.4	58612	18030	4
**Mg-CDIN1$^{D196A, E214A}$**	3 X 100 ns	94.7, 94.7, 94.7	91.5, 91.5, 91.5	58615	18034	2
**Codanin-1_CDIN1**	3 X 100 ns	99.2, 99.2, 99.2	94.6, 94.6, 94.6	65553	18523	13
**Codanin-1_CDIN1^{Y94C}**	3 X 100 ns	99.2, 99.2, 99.2	94.5, 94.5, 94.5	65510	18512	13
**Codanin-1 _CDIN1^{L178Q}**	3 X 100 ns	99.2, 99.2, 99.2	94.5, 94.5, 94.5	65521	18513	13
**CDIN1_DNA-RNA**	3 X 100 ns	102.5, 102.5, 102.5	98.7, 98.7, 98.7	74019	22731	44
**CDIN1_Codanin-1 _DNA-RNA**	3 X 100 ns	120.7, 120.7, 120.7	132.3, 132.3, 132.3	122014	36909	51
**Mg-CDIN1_DNA-RNA**	3 X 100 ns	104.6, 104.6, 104.6	101.6, 101.6, 101.6	80429	24349	42
**2Mg-CDIN1_DNA-RNA**	3 X 100 ns	104.1, 104.1, 104.1	100.2, 100.2, 100.2	77470	23882	40

Suppl. Fig. 1. Endogenous CDIN1 interacts with Codanin-1 and has a compact structure

A. Endogenous CDIN1 and Codanin-1 interact in the HUDEP-2 cell model. Endogenously C-terminal Flag-tagged CDIN1^{3xFLAG} was used in immunoprecipitation experiments followed by immunoblotting where the output (lane IP:FLAG) was blotted for CDIN1 and Codanin-1. Increasing salt concentrations (up to 500 mM NaCl) were used. CDIN1^{3xFLAG} and Codanin-1 interact and this interaction is resistant to high salt concentrations (500 mM) implying a tight association. Input: cell extract before immunoprecipitation; Supernatant (SN): Unbound fraction; IP: FLAG: Output fraction showing specific interaction partners of CDIN1.
B. AlphaFold3 model structure of human CDIN1 (AF-Q9Y2V0-F1), colours represent the per-residue confidence score (pLDDT) (left panel). Predicted active site residues E166, D196, E214 and K216 in CDIN1 are represented by Van der Waals (VDW) spheres (middle panel). Predicted aligned error (PAE) matrix showing confidence of the AlphaFold model is represented in the right panel. Expected position error colouring ranges from 0.00 to 30.00 Å and is displayed in a dark green-light green-white gradient.
C. RMSD (Root Mean Square Deviation) of Cα atoms (left top panel) and RMSF (Root Mean Square Fluctuations, bottom left panel) in each of the three 100 ns equilibrium MD (molecular dynamics) simulations for model structure of CDIN1. The right panel shows the structural representation of CDIN1, colouring is based on 0.0 to 5.00 Å of blue-white-red scale of the RMSF values.

Suppl. Fig. 2. Structure and molecular dynamics model of Codanin-1

A. AlphaFold3 model structure of full-length Codanin-1 (AF-Q8IWY9-F1), colours represent the per-reside confidence score (pLDDT) (left panel) and predicted aligned error (PAE) matrix showing confidence of the AlphaFold model is represented in the right panel. Expected position error colouring ranges from 0.00 to 30.00 Å and is displayed in a dark green-light green-white gradient.
B. AlphaFold3 model of full-length (light grey) Codanin-1 with the C-terminal region (residues 866-1227) of Codanin-1 in dark grey.
C. Convergence analysis of 3 × 100 ns of equilibrium MD simulations on the model structure of full-length Codanin-1 (left panel) compared to the C-terminal (residues 866-1227) of Codanin-1 (right panel). Cα of RMSD and RMSF as well as structural representation of Codanin-1 with RMSF colouring based on 0.0 to 5.00 Å of blue-white-red scale of the RMSF values are shown.

Suppl. Fig. 3. Structure and molecular dynamics model of the CDIN1-Codanin-1 complex

A. AlphaFold3 model structure of CDIN1 with the C-terminus of Codanin-1$^{866-1227}$, colours represent the per-reside confidence score (pLDDT) (left panel) and AlphaFold3 docked model of CDIN1 (light blue) in complex with Codanin-1$^{866-1227}$ (gray), active site residues of CDIN1 are highlighted as VDW spheres (middle panel). PAE matrix showing confidence of the AlphaFold model is represented in the right panel. Expected position error colouring ranges from 0.00 to 30.00 Å and is displayed in a dark green-light green-white gradient.

B. Structural analysis of 3 × 100 ns of equilibrium MD simulations on the model structure of the CDIN1-Codanin-1 complex. Cα RMSD of the bound complex and Cα RMSF values are shown in the top and bottom left panel. Cα RMSF representation of the bound complex structure is represented in the right panel, colouring is based on 0.0 to 7.00 Å of blue-white-red scale of the RMSF values.

C. Key binding interactions of the CDIN1-Codanin-1 complex during the course of 3 × 100 ns equilibrium MD simulations.

Suppl. Fig. 4. CDIN1-Codanin-1 forms a complex in an approximate 1:1 molar ratio

A. Size exclusion chromatogram for wild-type CDIN1-Codanin-1 (designated CDIN1-Codanin-1) and a control, predicted nuclease-inactive complex bearing alanine substitutions at residues D196 and E214 (CDIN1^{D196A,E214A}- Codanin-1). Wild-type CDIN1-Codanin-1 and CDIN1^{D196A,E214A}- Codanin-1 were run on a Superdex 200 16/60 and elute at 78 mL and 63 mL respectively. SDS-PAGE analysis of the fraction across the peak confirms the presence of both CDIN1 and Codanin-1 in the indicated fractions.

B. Intact mass spectrometry analysis of wild-type CDIN1-Codanin-1 and CDIN1^{D196A,E214A}- Codanin-1. LC-MS chromatogram shows the observed mass of all proteins is within range of the calculated mass.

C. Multiple sequence alignment showing the conserved residues in the predicted nuclease domain of CDIN1. Active site residues D196 and E214 are well conserved and were mutated to alanine in the predicted 'nuclease-dead' complex. Following species were used: Q551B4_DICDI; Q9vUU6_DROME; Q7Q6Y3_ANOGA; D6WU13_TRICA; Q9Y2V0_HUMAN, B7P753_IXOSC; E9HMN8_DAPPU; F7XKG7_METZD; D5E891_METMS; Q12UW9_METBU; Q8PUY2_METMA; D2RFH1_ARCPA; D3S125_FERPA; O29402_ARCFU; F2KPU2_ARCVS. Red: Conserved amino acid residues; Blue: Poorly conserved amino acid residues or acidic amino acids; Green: Hydroxyl, sulfhydryl and amine residues; Orange: Nonessential amino acids.

D. Analytic gel filtration trace of wild-type CDIN1-Codanin-1 on a Superdex S200 10/300 GL. Molecular weight standards were run on the column and are

indicated in the trace. The CDIN1-Codanin-1 elutes at a volume of 12.9 mL corresponding to an approximate mass of 130 kDa.

Suppl. Fig. 5. Structure and molecular dynamics models of the CDIN1 and CDIN1-Codanin-1 complex with an RNA:DNA substrate

A. AlphaFold3 model structure of CDIN1 with an RNA:DNA substrate, colours represent the per-reside confidence score (pLDDT) (left panel) and the docked model of CDIN1 (light blue) with an RNA:DNA substrate (RNA in light pink, DNA in light orange).
B. Structural analysis of 3 × 100 ns of equilibrium MD simulations on the model structure of CDIN1 with an RNA:DNA substrate. Cα RMSD and Cα RMSF values are shown in the left panel and a Cα RMSF representation of the bound complex structures of CDIN1 and RNA:DNA substrate is shown in the right panel, colouring is based on 0.0 to 5.00 Å of blue-white-red scale of the RMSF values.
C. AlphaFold3 model structure of the CDIN1-Codanin-1 complex with an RNA:DNA substrate, colours represent the per-reside confidence score (pLDDT) (left panel) and the docked model of CDIN1 (light blue)-Codanin-1 (grey) with an RNA:DNA substrate (RNA in light pink, DNA in light orange).
D. Structural analysis of 3 × 100 ns of equilibrium MD simulations on the model structure of the CDIN1-Codanin-1 complex with an RNA:DNA substrate. Cα RMSD and Cα RMSF values are shown in the left panel and a Cα RMSF representation of the bound complex structures of CDIN1-Codanin-1 and RNA:DNA substrate is shown in the right panel, colouring is based on 0.0 to 5.00 Å of blue-white-red scale of the RMSF values.
E. Model of the electrostatic potential of the CC domain of Codanin-1 complexed with an RNA:DNA substrate (RNA in light pink, DNA in light orange). The red colour indicates a negative potential and the blue colour indicates a positive potential. The substrate is nested within the positively-charged surface area of the CC domain of Codanin-1, potentially helping with its role in substrate stabilisation.

Suppl. Fig. 6. CDIN1-Codanin-1 is a RNA nuclease with a complex digestion pattern

A. CDIN1-Codanin-1 is an RNA nuclease with a complex digestion pattern, digesting from the 3'-end of the RNA strand in a 2-nucleotide step followed by a 1-nucleotide step fashion. Main products are labelled *, **, ***, **** and ***** corresponding to 18-mer, 16-mer, 14-mer, 13-mer and 12-mer respectively. A single nucleotide ladder was used to determine the size of the released products. CDIN1-Codanin-1 is able to digest ssRNA with a single cut (product *, 18-mer) whilst the complex shows a more complex digestion pattern on an

RNA:DNA hybrid where the RNA strand is labelled. Oligo 3 and 7 were used. More details on substrates can be found in Suppl. table 1A and B.

B. Time course assays containing 125 nM CDIN1-Codanin-1 for ssRNA and an RNA*:DNA substrate were used to define the linear range of product formation. Product formation (%) comparing CDIN1-Codanin-1 nuclease activity on ssRNA and RNA*:DNA over time was quantified as outlined in Methods and Materials. All data are shown as mean ± s.e.m. Oligo 3 and 7 were used. * indicates the labelled strand.
C. CDIN1-Codanin-1 activity is enhanced by the addition of $MgCl_2$, $MnCl_2$ and $ZnCl_2$. We determined 10 mM $MgCl_2$ as optimal for activity.
D. Consistent with its requirement for metal ions, CDIN1-Codanin-1 activity is inhibited by the addition of metal ion chelators EDTA, EGTA, and *o*-phenanthroline.

Increasing concentrations of protein (as indicated) were incubated with substrate at 37°C for 45 min and reactions were subsequently analysed by 20% denaturing PAGE to visualize product formation. For ions and chelators gels, 125 nM of protein was incubated with increasing concentrations of ions or chelators (as indicated) on ice for 15 min before adding substrate and incubating the reaction at 37°C for 45 min. Reactions were subsequently analysed by 20% denaturing PAGE to visualize product formation. Size of products was determined as shown in Suppl. Fig 6A. Main products are labelled *, **, ***, **** and ***** corresponding to 18-mer, 16-mer, 14-mer, 13-mer and 12-mer respectively. All oligos used are indicated in Suppl. table 1A and 1B.

Suppl. Fig. 7. CDIN1 is an ion-dependent RNA nuclease

A. Overlay of MD final snapshots of wild-type CDIN1 in light blue colour and mutant $CDIN1^{D196A,E214A}$ in teal colour having Mg^{2+} in the active site showing overall conformational changes upon ion-coordination loss.
B. Convergence analysis of 3 × 100 ns equilibrium MD simulations on the model structure of wild-type CDIN1 containing Mg^{2+} in the active site (WT) and mutant $CDIN1^{D196A,E214A}$ (MT). Cα RMSD and Cα RMSF values are shown in the left panel and a Cα RMSF representation of the structures are shown in the right panel, colouring is based on 0.0 to 5.00 Å of blue-white-red scale of the RMSF values. Mg^{2+} is presented by a green VDW sphere.
C. Electrophoretic mobility shift assays showing wild-type CDIN1-Codanin-1 and $CDIN1^{D196A,E214A}$ -Codanin-1 are able to bind to an RNA*:DNA hybrid, in the presence of both $MgCl_2$ and $CaCl_2$. CDIN1-Codanin-1 is also able to bind ssRNA and dsDNA, even though the complex has no activity on DNA. Increasing concentrations of protein (as indicated) were incubated with substrates for 15 min on ice, reactions were subsequently analysed by 20%

denaturing PAGE to visualise product formation. Oligos 3, 7 and 8 were used. * indicates the labelled strand.

D. CDIN1-Codanin-1 has no activity on DNA substrates mimicking common DNA replication intermediates or the DNA strand of an RNA:DNA substrate, even at concentrations 10x higher than used for all other assays. All DNA structures tested are indicated in the right panel. Increasing concentrations of protein (as indicated) were incubated with substrate at 37°C for 45 min and reactions were subsequently analysed by 20% denaturing PAGE to visualize product formation. Oligos used are listed in Suppl. table 1A and 1B.

Suppl. Fig. 8. CDIN1-Codanin-1 is a versatile RNA nuclease complex

A. CDIN1-Codanin-1 has no activity on substrates with an embedded ribonucleotide and requires a free 3’-RNA terminus for its activity.
B. CDIN1-Codanin-1 is not able to digest a substrate mimicking an ‘Okazaki-like’ structure or a substrate with a 5'-RNA section linked to a 3'-DNA section.
C. CDIN1-Codanin-1 is able to process common chemical modifications of RNA, namely 2-methyladenine (m^2A), 6-methyladenine (m^6A) whereas inosine (I) is inhibitory. An unmodified RNA:DNA substrate was used for reference.
D. Exonuclease T was used to demonstrate a classic RNA exonuclease activity. Increasing concentrations of protein (0 to 1 U) were incubated with substrate at 30°C for 45 min and reactions were subsequently analysed by 20% denaturing PAGE. With a substrate containing a 3'-biotin group, no activity is observed confirming ExoT has no endonuclease activity while the substrates with a 3'-hydroxyl or phosphate exhibit nearly identical product formation. Oligos 3, 5, and 6 were used. All oligos used are indicated in Suppl. Table 1A and 1B.
E. CDIN1-Codanin-1 is able to incise the RNA strand in an R-loop structure but where the loop structure is not a requirement for its activity. Product formation (%) was quantified as outlined in the Methods and Materials. All data are shown as mean ± s.e.m and at least three biological replicated were used for each substrate.
F. CDIN1-Codanin-1 has no discernible activity on a D-loop structure. Product formation (%) was quantified as outlined in the Methods and Materials. All data are shown as mean ± s.e.m and at least three biological replicated were used for each substrate.

Increasing concentrations of protein (as indicated) were incubated with substrate at 37°C for 45 min and reactions were subsequently analysed by 20% denaturing PAGE to visualize product formation. Size of products was determined as shown in Suppl. Fig 6A. Main products are labelled *, **, ***, **** and ***** corresponding to 18-mer, 16-mer, 14-mer, 13-mer and 12-mer respectively. Increasing concentrations of protein (as indicated) were incubated with substrate at 37°C for 45 min and reactions were subsequently analysed by

20% denaturing PAGE to visualize product formation. All oligos used are indicated in Suppl. table 1A and 1B.

Suppl. Fig. 9. Validation of our AF/MD structural model through site-directed mutagenesis

A. SDS-PAGE analysis of the final fraction of $CDIN1^{R22E}$, $CDIN1^{R26E}$, $CDIN1^{N146A}$, $CDIN1^{D171A}$, $CDIN1^{A160F}$, $CDIN1^{R101A}$, $CDIN1^{R126A}$, Codanin-1^{D1043K}, Codanin-1^{E1046K}, Codanin-1^{K1080A}, Codanin-1^{K1032A}, Codanin-1^{A1073F}, Codanin-1^{R872A} and Codanin-1^{K879A} confirms the presence of all mutant CDIN1 and Codanin-1 proteins.
B. Thermal shift assay traces of all generated mutants in CDIN1 and Codanin-1 show minimal changes in melting temperature compared to wild-type, indicating correct protein folding.
C. Analytical size-exclusion traces (run on a S200 increase 5/150 G/L column) showing all interaction mutants of CDIN1 and Codanin-1 co-migrate to form a complex, similar to wild-type complex.

Suppl. Fig. 10. Analysis of CDIN1 and Codanin-1 interaction mutants

A. Extended SPR parameters of analysis of interaction mutants in CDIN1 and Codanin-1 used to generate Fig. 5B. Apparent on and off rates as well as generated K_d values are displayed.
B. Analysis of DNA binding by fluorescence polarization of nucleic-acid mutant $CDIN1^{R101A}$ and $CDIN1^{R126A}$ compared to wild-type CDIN1. Both mutations in CDIN1 affect DNA binding, with around a 2-fold reduction in binding capacity compared to wild-type CDIN1 (K_d=2.5 μM compared to K_d=1.5 μM).

Suppl. Fig. 11. The patient mutants $CDIN1^{Y94C}$ and $CDIN1^{L178Q}$ form a complex with Codanin-1 *in vitro*

C. Size exclusion chromatogram for patient mutant complexes $CDIN1^{Y94C}$-Codanin-1 and $CDIN1^{L178Q}$-Codanin-1. $CDIN1^{Y94C}$-Codanin-1 and $CDIN1^{L178Q}$-Codanin-1 were run on a Superdex 75 and elute at 44.87 mL and 49.83 mL respectively. SDS-PAGE analysis of the fraction across the peak confirms the presence of both mutant CDIN1 and Codanin-1 in the indicated fractions.
D. Analytical size-exclusion trace showing patient mutant $CDIN1^{Y94C}$ and $CDIN1^{L178Q}$ and Codanin-1 co-migrate to form a complex. The identity of the present proteins in the mutant CDIN1-Codanin-1 peaks was confirmed by intact mass spectrometry, see Suppl. Fig. 9C.

E. Intact mass spectrometry analysis of patient mutant CDIN1^{Y94C}, CDIN1^{L178Q} and Codanin-1. LC-MS chromatogram shows the observed mass of all proteins is within range of the calculated mass.

Suppl. Fig. 12. The patient mutants CDIN1^{Y94C} and CDIN1^{L178Q} cause subtle but significant conformational changes to the CDIN1 predicted structure

A. Key binding interactions of the patient mutants CDIN1^{Y94C} and CDIN1^{L178Q} in complex with Codanin-1 during the course of 3 × 100 ns equilibrium MD simulations. No significant changes are observed.
B. Convergence analysis of 3 × 100 ns equilibrium MD simulations on the model structure of patient mutants CDIN1^{Y94C} and CDIN1^{L178Q} in complex with Codanin-1. Cα RMSD and Cα RMSF values are shown in the left panel and a Cα RMSF representation of the structures are shown in the right panel, colouring is based on 0.0 to 5.00 Å of blue-white-red scale of the RMSF values.
C. Convergence analysis of 3 × 100 ns equilibrium MD simulations on the model structure of patient mutants CDIN1^{Y94C} and CDIN1L178Q. Cα RMSD and Cα RMSF values are shown in the left panel and a Cα RMSF representation of the structures are shown in the right panel, colouring is based on 0.0 to 5.00 Å of blue-white-red scale of the RMSF values.

Suppl. Fig. 13. The patient mutants CDIN1^{Y94C} and CDIN1^{L178Q} cause subtle but significant conformational changes to the CDIN1 and Codanin-1 predicted structure

A. Overlay of MD-equilibrated wild-type CDIN1-Codanin-1 structure (light blue and grey) with CDIN1^{Y94C}-Codanin-1 (left panel, cyan and yellow) and CDIN1^{L178Q}-Codanin-1 (right panel, salmon and wheat) highlighting the conformational changes. Residues and RMSD values are indicated for the regions of conformational change. Notable, the Y94C substitution in CDIN1 has an effect on Codanin-1 conformation while the L178Q substitution shows milder effects.
B. Overlay of MD-equilibrated wild-type CDIN1 structure alone (light blue) with CDIN1^{Y94C} (left panel, cyan) and CDIN1^{L178Q} (right panel, salmon) highlighting the conformational changes. Residues and RMSD values are indicated for the regions of conformational change. Fluctuations are observed in both cases with increased conformation changes for CDIN1^{L178Q}.

Suppl. Fig. 14. The substrate-bound CDIN1 structures illustrating single- and di-Mg^{2+} coordination states.

A. The single-Mg^{2+} binding mode, with Mg^{2+} (green, Van der Waals sphere) coordinated by active-site residues and (b) The two-Mg^{2+} configuration, including the additional Mg^{2+} ion and its extended coordination network. These

models highlight that CDIN1 can accommodate both metal arrangements in the presence of substrate.

B. Convergence analysis of 3 × 100 ns equilibrium MD simulations on the model structure of CDIN1 with hybrid RNA/DNA substate containing single and di metal Mg^{2+} in the active site. a) Cα RMSD and b-d) Cα RMSF values are shown in the left panel and a Cα RMSF representation of the structures are shown in the right panel, colouring is based on 0.0 to 5.00 Å of blue-white-red scale of the RMSF values. Mg^{2+} is presented by a green VDW sphere.

Suppl. Fig. 15. *CDIN1* and *CDAN1* are co-dependent genes

A. Plot showing the 50 top hit for positively correlated co-dependent genes for *CDIN1* using the DepMap platform. The top hit is *CDAN1*. Interestingly, several key RNAses such as *RNAseH2* are also amongst the top hits.

Suppl. Fig. 16. Comparisons of AF3 predicted and experimental cryo-EM Codanin-1/ ASF-1 dimer models

A. Left panel shows the structure overlay with elements of the Codanin-1 dimer interface (labelled A' and B') indicated in the PAE plot shown on the left. The majority of the dimerization interface A' is formed from the N-terminal region of Codanin-1 whilst a secondary small interface (B') is formed by contacts involving the CC domain. This predicted part (B') of the interface is not found in the experimental cryo-EM structures 9IMZ and 9CVC and actually clashes with the ASF1-A2 subunit[10,11].

Suppl. Table 1: List of RNA and DNA oligonucleotide sequences used to generate simple and complex RNA and DNA-containing substrates

A. Each oligonucleotide sequence is numbered and a general description included.
B. To generate complex RNA and DNA structures, single-stranded oligos were annealed as outlined in the Materials and Methods and designated by the numbers.

Suppl. Table 2: Details of simulation time, box dimensions, number of water molecules and number of counter ions (salt concentration) in each simulation condition
