# Supplementary material for: CDIN1-Codanin-1 complex defective in congenital dyserythropoietic anaemia type I is an RNA nuclease
Source: Nat Commun. 2026 Jul 2;17:8253. doi: 10.1038/s41467-026-74766-7 (PMC13463051; doi:10.1038/s41467-026-74766-7)
Supplement: Supplementary file 2 [file 41467_2026_74766_MOESM2_ESM.pdf]

Description of Additional Supplementary Files

File name: Supplementary Data 1

Description:

List of all results obtained from immunoprecipitation followed by MS analysis of CDIN13xFLAG (previously C15orf41)

A. List of all proteins obtained from MS analysis for untagged HUDEP-2 cells (Control sample, referred to as wild-type (WT) across 4 repeats (1,2,3,4) obtained as outlined in Materials & Methods. PSM: peptide-spectrum mass.

and HUDEP-2 CDIN13xFLAG cells (Experimental sample, referred to as CDIN/C15orf41 across 4 repeats (1,2,3,4) obtained as outlined in Materials & Methods. PSM: peptidespectrum mass.

B. List of label-free quantification by normalised spectral abundance factor (NSAF) of all proteins obtained from MS analysis as outlined in Material & Methods. WT refers to untagged HUDEP-2 cells ; CDIN1 refers to CDIN13xFLAG cells across 4 repeats (1,2,3,4). SAF: spectral abundance factor.

C. List of Power law global error model (PLGEM) statistics for all proteins obtained from MS analysis obtained as outlined in Materials & Methods. IPCDIN1 refers to experimental sample ; Control refers to untagged sample. STN: Signal-to-noise
