# Supplementary material for: CDIN1-Codanin-1 complex defective in congenital dyserythropoietic anaemia type I is an RNA nuclease
Source: Nat Commun. 2026 Jul 2;17:8253. doi: 10.1038/s41467-026-74766-7 (PMC13463051; doi:10.1038/s41467-026-74766-7)
Supplement: Supplementary file 3 [file 41467_2026_74766_MOESM4_ESM.pdf]

nature portfolio

# Peer Review File

# CDIN1-Codanin-1 complex, defective in Congenital Dyserythropoietic Anaemia type I, is an RNA nuclease

Corresponding Author: Professor Peter McHugh

**This file contains all reviewer reports in order by version, followed by all author rebuttals in order by version.**

Version 0:

Reviewer comments:

Reviewer #1

(Remarks to the Author)
The manuscript entitled “The CDIN1-Codanin-1 complex….is a RNA nuclease” describes a combination of cellular, biochemical, and computational studies that jointly demonstrate that CDIN1is a novel 3’-5 exo-ribonuclease, that is able to digest a variety of single-stranded and double-stranded RNA substrates, that requires a free RNA 3’ terminus and a minimal length of substrate for substrate digestion, and that requires association with codanin-1 for fully activation of its enzymatic activities (particularly towards single-stranded RNA). The study also demonstrates the effect of a pair of point mutations associated with CDA-I (Congenital Dyserythropoietic Anaemia type-I) on enzyme activity.

The study is built around:

1. Demonstration of protein-protein association using FLAG-tagged CDIN1 in a HUDEP-2 cell line to directly pull down untagged Codanin-1 (the reverse experiment does not appear to have been conducted, but the results of this experiment appear to be reliable).

2. Validation of protein-protein interaction with an apparent low- to slightly sub-micromolar affinity using three mutually orthogonal approaches: SEC, ITC and SPR.

3. Molecular modeling of the individual protein subunits, the protein-protein complex, and protein-nucleic acid complex(es) using AlphaFold.

4. Molecular dynamics simulations of the resulting AF models, that (in particular) provide insight into the register of substrate strands in the enzyme PD-D/E-xK active site of CDIN1.

5. Biochemical analyses of the effect of RNA length, chemical composition, and structure on activity.

6. Demonstration of the effect of two disease-associated mutations on RNAase activity.

Overall, this is a very straightforward, rigorous study that presents an over-arching set of conclusions (stated above) that appear to be well-supported by rigorous experimentation and appropriate conclusions based on the results of those experiments. This reviewer only offers relatively minor queries and suggestions for the authors’ attention.

1. The authors state that they tested the effect of individual point mutations in the nuclease active site on RNase activity (line 370 and onwards) and saw a loss of activity, as expected. However, this reviewer was not able to spot any experiments in which mutations were generated within the predicted protein-protein interface that might be expected to abrogate or interfere with the interaction of codanin-1 with CDIN1 (and thereby block the ability of codanin-1 to accentuate the latter's enzymatic activity, particularly towards ssRNA substrates. For a study that relies entirely on computational prediction, that would seem to this reviewer to be a rather important line of validation of the modeling exercise.

2. The ITC isotherm presented in Figure 1E does not appear to present a sigmoidal approach to baseline as the number of

injections (and the corresponding total concentration of the analyte protein) increase. Generally, such isotherms are somewhat concerning and indicative of a pathology in the experiments, that might range from incorrect protein concentration assumptions, to incomplex or inadequate mixing at each injection, to instrument mis-calibration or misfunction, to buffer mismatch or incompatibility, to equilibration times during each injection that are too fast or slow for the experiment.

Since the experiment does in fact indicate an exothermic binding event and gives a Kd value that is not far away from the SPR-based measurement, the experimental data can probably stay, but a sentence mentioning this issue might be warranted.

3. The title appears to have a grammatical error: shouldn't it ready "…is an RNA nuclease" instead of "in a RNA nuclease" (?)

4. I had to stop everything and look up both "enucleation" and "pathognomonic" before continuing with my reading of the introduction. This reviewer would recommend removing those two jargon-ish terms and replacing them with simpler, more explicit phrases that correspond to their definitions.

5. I would personally be interested in whether the authors put any effort into actually determining the structure of the protein complex (130 kD) in the absence or presence of dsRNA and calcium (should add about 10 kD) using either X-ray crystallography or CryoEM. The lack of such studies is not a deal-breaker for me (although in their absence, the suggested examination of point mutations predicted to block protein-protein association and test the quality of the modeled interface would seem all the more important) but I think a statement regarding such an attempt might be warranted.

Reviewer #2

(Remarks to the Author)
The manuscript by S. Brolih et al. investigates the enzymatic function of the Codanin-1–CDIN1 complex and its potential role in the pathogenesis of Congenital Dyserythropoietic Anaemia type I (CDA-I). CDA-I is a rare inherited disorder characterized by defective erythropoiesis and a distinctive 'spongy' heterochromatin nuclear phenotype, primarily caused by mutations in CDAN1 or CDIN1. The authors demonstrate that CDIN1 and Codanin-1 form a stable complex with ribonucleolytic activity against both dsRNA-DNA hybrids and ssRNA. Functional analysis of disease-associated mutations provides novel insights into the molecular mechanisms contributing to CDA-I.
Major points:
Introduction part:
Please clarify that the CDA-I disease protein referred to as CDA-1 is also known as Codanin-1 (CDAN1) to avoid confusion. The current wording is unclear to readers unfamiliar with the terminology.
The sentence referencing Codanin-1's homology to CNOT1 as supporting evidence for its scaffolding role with ASF1A/ASF1B and CDIN1 is ambiguous. The authors should elaborate on how this homology supports the proposed role—particularly for readers outside the field.
The statement regarding "L178Q CDA-I patient mutations on nuclease activity, providing a possible explanation for the disease phenotype" requires clarification. How does a non-specific ss/dsRNase activity relate directly to a cellular phenotype such as spongy heterochromatin? If possible, the authors should provide mechanistic insights or direct evidence to clarify.
Results part:
In Figure 1, please include a domain overview of both Codanin-1 and CDIN1. Indicate the exact residues used in the AlphaFold models, as this is essential for interpretation—especially in light of the recent cryo-EM structures of Codanin-1 in complex with ASF1, which show structural flexibility in the same regions. The current text (e.g., "We next examined the structure of CDIN1 in complex with Codanin-1…") lacks sufficient detail. Clearly specify which regions of Codanin-1 are included in the AlphaFold model, as this is not apparent in the figure or description.
The active site mentioned in line 182 is supposedly in a β-sheet but is barely visible in Supplementary Fig. 1B. Please enhance visibility or provide a more detailed zoom-in view.
The structural figures show surfaces around the models, which may mislead readers into thinking these are experimentally solved structures. AlphaFold models, even with high confidence, are predictions and should be clearly labeled as such. Consider removing the surface renderings or clearly distinguishing predicted from validated regions.
The Codanin1–CDIN1 complex model in Fig. 1 would be more informative if the predicted interaction interfaces were highlighted. Include close-up views with annotated residues and some degree of surface complementarity analysis. Functional validation via mutational and biochemical interaction studies would significantly strengthen the conclusions made using the models. Similarly, the AlphaFold substrate binding models are currently hypothetical. Please provide biochemical evidence for the predicted ds DNA-RNA binding sites, such as mutagenesis of the proposed interface residues.
The AlphaFold predictions are modeled as 1:1 Codanin/CDIN1 complexes, whereas size exclusion chromatography and the published cryo-EM data (Sedor et al.) indicate a dimeric Codanin. Please explain why the AlphaFold models were not constructed as heterotetramers, and discuss how dimerization may affect the predictions.
All substrate binding models are presented using the monomeric complex, which conflicts with the reported oligomeric state. Please also clarify whether the conformation of the dsRNA-DNA–bound model differs from, or resembles, that of the apo Codanin–CDIN1 complex.
Figure 3C: The color distinction between the curves is insufficient—particularly between light blue and light grey in the legend. Please revise for better visual accessibility (e.g., by using more contrasting colors or line patterns).

Figure 4: The RNA-DNA digestion assay includes a CDIN1 active site mutant control, but a similar control for ssRNA digestion is missing. This control is critical and should be included.
Figure 5 and related text: In my opinion, the differences between the AlphaFold models of the point mutants and the wild-type protein are not particularly informative and do not significantly enhance our understanding of the disease. A close-up view of the mutation site, along with an indication of the nuclease active site's position relative to the mutation, would be more helpful.
In conclusion, the authors convincingly identify a novel 3'-5' RNA exonuclease activity for CDIN1 and demonstrate that Codanin-1 modulates this activity. Their findings establish that the Codanin–CDIN1 complex can digest a variety of ss- and ds-RNA substrates. However, the AlphaFold-based structural models remain hypothetical without experimental validation. The predicted protein-protein and protein-substrate interfaces must be tested through point mutations and biochemical assays. Moreover, the use of 1:1 complex models is inconsistent with biochemical and cryo-EM evidence for a 2:2 stoichiometry, which should be addressed. Clarifying these structural and functional points would significantly enhance the manuscript's quality.

Reviewer #3

(Remarks to the Author)
Brolih et al describe a characterization of CDIN1-Codanin-1 complex, which is a novel nuclease. Mutations in the two proteins lead to a rare genetic anemia CDA-I. In the first part of this work the authors confirmed that CDIN1 and Codanin-1 interact using immunoprecipitation from erythroid cells. Next, they prepared AlphaFold models of the two proteins and their complex. Stability of these models was verified using Molecular Dynamics. Interaction of the two proteins was further investigated using biophysical methods. Recombinant stabilized version of CDIN1 with three point mutations and C-terminal region of Codanin-1 (residues 866-1227) were used. The authors then demonstrated a weak RNase activity of this complex and generated AlphaFold models of complexes between RNA/DNA and CDIN1 or CDIN1-Codanin-1. The latter showed that Codanin-1 may participate in the stabilization of nucleic acid. Indeed, in vitro the complex of the two proteins had higher RNase activity. Activity assays with numerous nucleic acid substrates led authors to conclude that CDIN1 is a 3'-5' RNA exonuclease. In the last part two pathogenic mutations in CDIN1 were studied which showed that they affect the nuclease activity.

Overall, this study is technically sound and concerns an interesting protein complex with relevance to human disease. However, in the opinion of this referee this manuscript contributes limited new information to our understanding of the activities, mechanism of action and role of this complex.

Many conclusions of the paper are based on computational methods – AlphaFold predictions coupled with MD simulations. However, the paper does not describe experimental validation of the computational structural models. The most desired validation would include structural studies or mass spectrometry after chemical cross-linking. However, mutagenesis of the residues predicted to be involved in protein-protein or protein-nucleic acid contacts and checking the effect on complex stability and enzymatic activity would already provide some support for the structural models. The evolutionary conservation of the residues predicted to participate in contacts is not discussed.

Importantly, the structural predictions should provide information which would help understand the mechanism of the studied complex, for example the structural basis of the specificity of the nuclease for RNA. This should be readily testable using relevant protein variants with point substitutions or modified substrates. Another basic check of the correctness of their modelling, which is not shown, is the analysis of the interactions of the phosphodiester backbone (in particular scissile phosphate) of the RNA with the active site in their models. Is the trajectory of the cleaved RNA strand, its polarity and positioning similar to those observed in experimental structures of other PD(D/E)XK nucleases? Finally, the model of the complex interacting with RNA/DNA suggests that Codanin-1 provides additional contacts with the nucleic substrate. Therefore, CDIN-1 alone should have lower affinity for the nucleic acid than the complex of the two proteins which has not been tested.

It is important to note that the nuclease activity observed in the in vitro assays is rather weak. 25-fold molar excess of the nuclease and 6 to 12.5-fold molar excess of the complex over substrate was required to observe nuclease activity. This is not unprecedented that in vitro nuclease assays require excess of the enzyme. However, how can the Authors ensure that the observed activity is physiological given that such high nuclease concentration needs to be used in the vitro assay? On a related note Supplementary Fig 8E appears to show that CDIN1-Codanin-1 has much more robust activity on an R-loop-like substrate. This is not explored further or discussed.

Authors also used in silico approaches to study the interactions of $Mg^{2+}$ cofactor with the active site. These analyses showed binding of one metal ion. They are, however, probably not relevant to the actual catalytic mechanism, because two metal ions are involved in nucleic acid hydrolysis and they would be properly organized at the active site in the presence of the appropriately bound nucleic acid.

In summary, this manuscript does not provide sufficient validation of the computational results. The main finding is the demonstration of the RNase activity of CDIN1 and the modification of this activity by Codanin-1. This is however, relatively limited new information.

Minor points:
Line 164: the title of the second section states "in vitro interaction" of the two proteins which is misleading because this

section describes in silico modelling and MD simulations.

The presentation of the data could be improved by including domain schematics of the studied proteins. For example, in Fig. 1 the model labeled Codanin-1 is in fact the C-terminal module and only after checking the Supplementary Fig. 2 it becomes apparent that this is only a part of the structure of the protein.

The Authors need to discuss whether the mutations they introduce to stabilize CDIN1 could affect nucleic acid binding, enzymatic activity and interactions with Codanin-1.

Line 336: "negatively-charged face forms an extensive contact with the RNA-DNA substrate". It is usually positively charged patches that interact with the phosphodiester backbone of nucleic acids. Is this a typo?

Version 1:

Reviewer comments:

Reviewer #1

(Remarks to the Author)
The authors have satisfactorily responded to and addressed the queries and suggestions offered in peer review.

Reviewer #2

(Remarks to the Author)
Referee Report
I thank the authors for their revised manuscript and for their response to my previous referee report. However, several of the major concerns raised earlier have still not been adequately addressed. In some cases, the authors state in their response that changes were implemented (e.g. Figure 1 and inclusion of a domain overview), but these changes are either not evident in the manuscript or do not sufficiently resolve the issues raised. Overall, while the biochemical data are interesting and suggest that the Codanin-1–CDIN1 complex exhibits RNA exonuclease activity that is enhanced by Codanin-1, substantial clarification, correction, and more careful interpretation are still required. In addition, brackets are repeatedly used for already published or well-established results; these should generally be removed to improve clarity and tone.
Major Points
- Lack of a clear domain overview in Figure 1
There is still no meaningful domain overview of Codanin-1 in Figure 1. In particular, I cannot identify a clear diagram indicating which Codanin-1 domains are excluded in the modeling. This was explicitly requested in my previous report and remains unresolved.
- Neglect of Codanin-1 / CDIN1 dimerization
At line 87, the phrase "(potentially homodimeric)" is placed in brackets, which is misleading. Cryo-EM and accompanying biochemical data by Jeong et al. clearly demonstrate that the Codanin-1–CDIN1–ASF1 complex, as well as the Codanin-1–CDIN1 complex, are dimeric. This established finding is neither properly acknowledged nor discussed in the Introduction or Results, despite its obvious relevance for the structural and functional interpretation of the data presented here.
- Figure 1B: labeling and Western blot interpretation
In Figure 1B, the Codanin-1 label appears to be positioned at an incorrect height. In addition, a second band is visible in the Codanin-1 Western blot. Please clarify what this band represents.
- SPR (Biacore) data interpretation (Figure 1F)
In the Biacore experiment shown in Figure 1F, the association curves flatten at high protein concentrations. This behavior suggests that the interaction may be limited by surface saturation or mass transport rather than intrinsic binding kinetics. While the reported KD value is likely within the correct range, applying a global kinetic fit to the SPR data—excluding concentrations affected by surface saturation—would further validate the binding analysis.
- Unclear sentence at page 3, line 96
The sentence starting at line 96 on page 3 remains unclear and poorly written and should be rewritten for clarity.

- Misleading surface representations
Surface representations that give the impression of experimentally determined cryo-EM structures are still present in the supplementary figures. These representations are misleading and should be revised or clearly labeled as models.

- Critical residue mislabeling (K216 vs R216)
In Supplementary Figures 1, 2, 4, 6, and Supplementary Figure 14, residue 216 is labeled as R216, whereas the correct residue is Lysine (K216), as shown in the sequence and in the structural representations. Since this residue is part of the catalytic triad, this error is substantial and must be corrected in all figures. Notably, in the sequence alignment in Supplementary Figure 4, the residue is correctly labeled as K216.
- Missing consideration of the dimeric Codanin-1–CDIN1 complex in AF3 modeling
I ran AlphaFold3 on the dimeric Codanin-1–CDIN1 complex, and the prediction suggests that the dimer interface may interfere with the CDIN1 binding site proposed in this manuscript when considering the full-length protein. At minimum, the authors should comment on this issue. Ideally, they should show an AF3 prediction of the Codanin-1 dimer or overlay the

dimeric cryo-EM structure with their complex prediction (e.g. in Supplementary Figure 2 and page 7, line 228). While I understand that the interacting C-terminus of Codanin-1 may be flexible and potentially unaffected by dimerization, it would still be important to show where the N-terminal ~865 residues would be located and how a full-length dimeric complex could be arranged. Furthermore, the authors demonstrate that Codanin-1–CDIN1 forms a dimer–dimer complex; it is therefore unclear why this configuration was not used for the AF3 prediction, which is technically feasible.
- Excessively long subtitles
The very long subtitles (e.g. "Cloning and site directed mutagenesis of CDIN1S … Codanin-1 complex") should be shortened. For example, this subtitle could simply be changed to:
"Cloning and site-directed mutagenesis of CDIN1 and Codanin-1."
- Overstatement of mutational analysis
In the Results/Discussion section (around line 650), the conclusions drawn from the mutational analysis are overstated. While some mutations support the predicted interface, others do not show an effect. In addition, several patient mutations not located in the predicted interface have a stronger impact on nuclease activity. A more careful formulation would be appropriate, for example:
"The mutational analysis partially supports the predicted model. Notably, several patient-derived mutations outside the proposed interface show a more pronounced effect on nuclease activity."
Minor Points:
Line 593: "Cadanin-1-CDIN1" should be corrected to "Codanin-1–CDIN1."

Line 745: In the phrase "and Codanin-1 and demonstrate for that the," please remove "for."

Line 756: In the sentence referring to previous studies, remove "neither study."

Line 800: Please clarify the question mark in "The footprint of the CC domain may offer an explanation as to why a strong preference?"

Line 818: Please clarify whether the statement refers to something that is likely lethal or has been experimentally shown to be lethal.
Conclusion
In conclusion, while the biochemical data are intriguing and generally support a model in which the Codanin-1–CDIN1 complex exhibits RNA exonuclease activity that is enhanced by Codanin-1, key structural and mechanistic aspects remain insufficiently addressed. In particular, the absence of a dimer-aware AF3 model (with or without nucleic acid) or at least a critical discussion thereof, as well as the issues with SPR and ITC data interpretation, weaken the overall conclusions. The Discussion section requires revision to more accurately reflect the limitations of the current analyses.
I therefore still recommend revision addressing the points above.

Reviewer #3

(Remarks to the Author)
The authors appropriately addressed my comments and introduced new data, thereby improving the manuscript.

I have one additional comment: the fits in Figure 5C seem unusual. The experimental points are far from the fitted binding curve, and it is not clear whether the values in the table confirm the importance of the selected residues. Plots for each variant should be shown separately so that readers can evaluate the quality of the fit.

Version 2:

Reviewer comments:

Reviewer #2

(Remarks to the Author)
The authors have satisfactorily addressed all of my comments and questions, and I have no further concerns.

**Open Access** This Peer Review File is licensed under a Creative Commons Attribution 4.0 International License, which permits use, sharing, adaptation, distribution and reproduction in any medium or format, as long as you give appropriate credit to the original author(s) and the source, provide a link to the Creative Commons license, and indicate if changes were made.
In cases where reviewers are anonymous, credit should be given to 'Anonymous Referee' and the source.
The images or other third party material in this Peer Review File are included in the article's Creative Commons license, unless indicated otherwise in a credit line to the material. If material is not included in the article's Creative Commons license and your intended use is not permitted by statutory regulation or exceeds the permitted use, you will need to obtain permission directly from the copyright holder.
To view a copy of this license, visit https://creativecommons.org/licenses/by/4.0/

**Reviewer #1 (Remarks to the Author):**

The manuscript entitled “The CDIN1-Codanin-1 complex….is a RNA nuclease” describes a combination of cellular, biochemical, and computational studies that jointly demonstrate that CDIN1 is a novel 3’-5 exo-ribonuclease, that is able to digest a variety of single-stranded and double-stranded RNA substrates, that requires a free RNA 3’ terminus and a minimal length of substrate for substrate digestion, and that requires association with codanin-1 for fully activation of its enzymatic activities (particularly towards single-stranded RNA). The study also demonstrates the effect of a pair of point mutations associated with CDA-I (Congenital Dyserythropoietic Anaemia type-I) on enzyme activity.

The study is built around:

1. Demonstration of protein-protein association using FLAG-tagged CDIN1 in a HUDEP-2 cell line to directly pull down untagged Codanin-1 (the reverse experiment does not appear to have been conducted, but the results of this experiment appear to be reliable).

2. Validation of protein-protein interaction with an apparent low- to slightly sub-micromolar affinity using three mutually orthogonal approaches: SEC, ITC and SPR.

3. Molecular modeling of the individual protein subunits, the protein-protein complex, and protein-nucleic acid complex(es) using AlphaFold.

4. Molecular dynamics simulations of the resulting AF models, that (in particular) provide insight into the register of substrate strands in the enzyme PD-D/E-xK active site of CDIN1.

5. Biochemical analyses of the effect of RNA length, chemical composition, and structure on activity.

6. Demonstration of the effect of two disease-associated mutations on RNAase activity.

Overall, this is a very straightforward, rigorous study that presents an over-arching set of conclusions (stated above) that appear to be well-supported by rigorous experimentation and appropriate conclusions based on the results of those experiments. This reviewer only offers relatively minor queries and suggestions for the authors’ attention.

1. The authors state that they tested the effect of individual point mutations in the nuclease active site on RNase activity (line 370 and onwards) and saw a loss of activity, as expected. However, this reviewer was not able to spot any experiments in which mutations were generated within the predicted protein-protein interface that might be expected to abrogate or interfere with the interaction of codanin-1 with CDIN1 (and thereby block the ability of codanin-1 to accentuate the latter's enzymatic activity, particularly towards ssRNA substrates. For a study that relies entirely on computational prediction, that would seem to this reviewer to be a rather important line of validation of the modeling exercise.

We thank the reviewer for their careful reading of the manuscript, and helpful, constructive suggestions.

This first point is also raised by the other reviewers: the need to generate mutations that interfere with the protein-protein interaction between Codanin-1 and CDIN1, as well as between the complex and nucleic acids. We agree that this is an important validation step to test our modelling data. We have carried out extensive mutagenesis, coupled with purification of the variant forms of the protein, to address these points.

New interaction mutants and substrate binding mutants made based on structure predictions and quantitatively validated with surface plasmon resonance, analytical size exclusion and substrate-binding assays (FP). Please see New Fig 5 as well as further information added to Fig 3 (and Suppl. Fig 9 and Suppl. Fig 10) and associated text on pages 18-20.

2. The ITC isotherm presented in Figure 1E does not appear to present a sigmoidal approach to baseline as the number of injections (and the corresponding total concentration of the analyte protein) increase. Generally, such isotherms are somewhat concerning and indicative of a pathology in the experiments, that might range from incorrect protein concentration assumptions, to incomplex or inadequate mixing at each injection, to instrument mis-calibration or misfunction, to buffer mismatch or incompatibility, to equilibration times during each injection that are too fast or slow for the experiment.

Since the experiment does in fact indicate an exothermic binding event and gives a Kd value that is not far away from the SPR-based measurement, the experimental data can probably stay, but a sentence mentioning this issue might be warranted.

We accept this point and suspect that a non-ideal buffer match may be the likely culprit. In this paper we wanted to use as many orthogonal techniques as possible (SPR, ITC and SEC) to demonstrate that the interaction between CDIN1 and Codanin-1 is robust. As requested, we have added wording to place a caveat around this ITC data on page 10:

*'Isothermal titration calorimetry (ITC) experiments confirm a direct interaction between CDIN1 andCodanin-1 with an apparent affinity in the low µM range ($K_d$= 1.66 ± 0.54 µM) (Fig. 1E). We note that the ITC isotherm does not show a sigmoidal approach to the baseline as the analyse concentration increases, indicating a possible buffer mismatch or other pathology in the data. Nevertheless, the ITC data confirm an exothermic binding event with approximate 1:1 stoichiometry confirming that CDIN1 and the C-terminal 866-1227 amino acids of Codanin-1 form a stable high-affinity complex.'*

3. The title appears to have a grammatical error: shouldn't it ready "…is an RNA nuclease" instead of "in a RNA nuclease" (?)

Correct, and amended.

4. I had to stop everything and look up both "enucleation" and "pathognomonic" before continuing with my reading of the introduction. This reviewer would recommend removing those

two jargon-ish terms and replacing them with simpler, more explicit phrases that correspond to their definitions.

We agree, and have changed the language to a more accessible wording.

5. I would personally be interested in whether the authors put any effort into actually determining the structure of the protein complex (130 kD) in the absence or presence of dsRNA and calcium (should add about 10 kD) using either X-ray crystallography or CryoEM. The lack of such studies is not a deal-breaker for me (although in their absence, the suggested examination of point mutations predicted to block protein-protein association and test the quality of the modeled interface would seem all the more important) but I think a statement regarding such an attempt might be warranted.

We did try quite extensively to crystallize both the complex and CDIN1 alone, both in the presence or absence of DNA, DNA/RNA hybrids and RNA. Ultimately, we did not get any crystals. As the reviewer mentioned and in our experience with other systems, it is important to try and prevent the nuclease from digesting its substrate, and we tried to use DNA (not cleaved by CDIN1) or calcium ions (prevent cleavage in our assays) but ultimately failed. We note that this complex also appears challenging for Cryo-EM likely due to its dynamic nature, as even the two recent studies of Codanin-1 structure by Cryo-EM (Sedor *et al*, 2024 and Jeong *et* al 2024) failed to resolved the C-terminal part of Codanin-1 and CDIN1 that is studied in our paper.

**Reviewer #2 (Remarks to the Author):**

The manuscript by S. Brolih et al. investigates the enzymatic function of the Codanin-1–CDIN1 complex and its potential role in the pathogenesis of Congenital Dyserythropoietic Anaemia type I (CDA-I). CDA-I is a rare inherited disorder characterized by defective erythropoiesis and a distinctive 'spongy' heterochromatin nuclear phenotype, primarily caused by mutations in CDAN1 or CDIN1. The authors demonstrate that CDIN1 and Codanin-1 form a stable complex with ribonucleolytic activity against both dsRNA-DNA hybrids and ssRNA. Functional analysis of disease-associated mutations provides novel insights into the molecular mechanisms contributing to CDA-I.

Major points:

Introduction part:
Please clarify that the CDA-I disease protein referred to as CDA-1 is also known as Codanin-1 (CDAN1) to avoid confusion. The current wording is unclear to readers unfamiliar with the terminology.

Throughout, we thank the reviewer for their constructive comments. We agree with this point have clarified this wording.

The sentence referencing Codanin-1's homology to CNOT1 as supporting evidence for its scaffolding role with ASF1A/ASF1B and CDIN1 is ambiguous. The authors should elaborate on how this homology supports the proposed role—particularly for readers outside the field.
The statement regarding "L178Q CDA-I patient mutations on nuclease activity, providing a possible explanation for the disease phenotype" requires clarification. How does a non-specific ss/dsRNase activity relate directly to a cellular phenotype such as spongy heterochromatin? If possible, the authors should provide mechanistic insights or direct evidence to clarify.

We have further qualified the potential leads derived from the observed homology between Codanin-1 and CNOT1. The Discussion at the end of the article has been amended to make the potential link between patient pathology and the observed biochemical activity of the Codanin-1-CDIN1 complex more explicit, as we think this is the appropriate place to address this issue.

Results part:
In Figure 1, please include a domain overview of both Codanin-1 and CDIN1. Indicate the exact residues used in the AlphaFold models, as this is essential for interpretation—especially in light of the recent cryo-EM structures of Codanin-1 in complex with ASF1, which show structural flexibility in the same regions. The current text (e.g., "We next examined the structure of CDIN1 in complex with Codanin-1…") lacks sufficient detail. Clearly specify which regions of Codanin-1 are included in the AlphaFold model, as this is not apparent in the figure or description.

Thank you for this request, with which we agree, and we have amended to improve this section. We have now revised Figure 1 to include a domain overview of both Codanin-1 and CDIN1, clearly indicating the regions used in the AlphaFold-Multimer (AF3) modelling. This includes the key interacting residues and the stability of these interactions as observed during molecular dynamics (MD) simulations. We have also updated the text in the manuscript for improved clarity. The revised sentence now reads:

*"We next examined the predicted structure of CDIN1 in complex with Codanin-1, using AF3[24], focusing on full-length (1–281) of CDIN1 and the C-terminal region comprising residues 866–1227 of Codanin-1 (Fig. 1C, right panel; Suppl. Fig. 3A)."*

The active site mentioned in line 182 is supposedly in a β-sheet but is barely visible in Supplementary Fig. 1B. Please enhance visibility or provide a more detailed zoom-in view.
The structural figures show surfaces around the models, which may mislead readers into thinking these are experimentally solved structures. AlphaFold models, even with high confidence, are predictions and should be clearly labelled as such. Consider removing the surface renderings or clearly distinguishing predicted from validated regions.

Thanks, we agree. To address this, we have removed the surface renderings from all structural figures in both the main paper and the Supplementary Information to avoid any confusion regarding the nature of the models. We have also ensured that all AlphaFold-derived structures are clearly labelled as predicted models in the figure legends and main text.

The Codanin1–CDIN1 complex model in Fig. 1 would be more informative if the predicted interaction interfaces were highlighted. Include close-up views with annotated residues and some degree of surface complementarity analysis. Functional validation via mutational and biochemical interaction studies would significantly strengthen the conclusions made using the models. Similarly, the AlphaFold substrate binding models are currently hypothetical. Please provide biochemical evidence for the predicted ds DNA-RNA binding sites, such as mutagenesis of the proposed interface residues.

This is addressed in our revised Figure 1, as noted in our earlier response. We have included close-up views highlighting the predicted interaction interfaces between Codanin-1 and CDIN1, with annotated key residues. Additionally, all interacting residues were observed to form stable interactions throughout the course of the MD simulations, supporting the reliability of the predicted interface.

We have also carried out extensive mutagenesis, coupled with purification of the variant forms of the protein, to address this important point. New interaction mutants and substrate binding mutants made based on structure predictions and quantitatively validated with surface plasmon resonance, analytical size exclusion and substrate-binding assays (FP). Please see New Fig 5 as well as further information added to Fig 3 (and Suppl. Fig 9 and Suppl. Fig 10) and associated text on pages 18-20.

The AlphaFold predictions are modeled as 1:1 Codanin/CDIN1 complexes, whereas size exclusion chromatography and the published cryo-EM data (Sedor et al.) indicate a dimeric Codanin. Please explain why the AlphaFold models were not constructed as heterotetramers, and discuss how dimerization may affect the predictions.

Thank you for raising this. In our current study, the AlphaFold3 models were generated as 1:1 Codanin-1: CDIN1 complexes to focus specifically on the direct interaction interface between the two proteins. Modelling the full heterotetrameric complex was beyond the current scope due to limitations in AlphaFold-Multimer when handling large, multi-chain assemblies.

Additionally, cryo-EM studies involving flexible or fragmentary proteins, such as the C-terminal region of Codanin-1, often yield multimeric states, yet the overall interaction interface and binding mode typically remain consistent.

All substrate binding models are presented using the monomeric complex, which conflicts with the reported oligomeric state. Please also clarify whether the conformation of the dsRNA-DNA–bound model differs from, or resembles, that of the apo Codanin–CDIN1 complex.

Thank you for your observation. As shown in new Fig. 3B, we have now performed a comparative analysis between the apo and the dsRNA-DNA–bound models. Overall, the binding modes and key interactions remained largely consistent between the two states. Notably, the CC domain of the Codanin-$1^{866\text{-}1227}$ region exhibits conformational flexibility, adopting a more open configuration in the bound model to accommodate both RNA and DNA substrates. This conformational adaptability helps reconcile the observed binding with the reported oligomeric

state. We have now added these details and clarifications to the related main text of the manuscript for improved clarity and completeness.

Figure 3C: The color distinction between the curves is insufficient—particularly between light blue and light grey in the legend. Please revise for better visual accessibility (e.g., by using more contrasting colors or line patterns).

This has been improved throughout.

Figure 4: The RNA-DNA digestion assay includes a CDIN1 active site mutant control, but a similar control for ssRNA digestion is missing. This control is critical and should be included.

We agree -- this has been added to amended Fig. 4A

Figure 5 and related text: In my opinion, the differences between the AlphaFold models of the point mutants and the wild-type protein are not particularly informative and do not significantly enhance our understanding of the disease. A close-up view of the mutation site, along with an indication of the nuclease active site's position relative to the mutation, would be more helpful.

We agree and have amended accordingly – please see new Fig. 6C.

In conclusion, the authors convincingly identify a novel 3'-5' RNA exonuclease activity for CDIN1 and demonstrate that Codanin-1 modulates this activity. Their findings establish that the Codanin–CDIN1 complex can digest a variety of ss- and ds-RNA substrates. However, the AlphaFold-based structural models remain hypothetical without experimental validation. The predicted protein-protein and protein-substrate interfaces must be tested through point mutations and biochemical assays. Moreover, the use of 1:1 complex models is inconsistent with biochemical and cryo-EM evidence for a 2:2 stoichiometry, which should be addressed. Clarifying these structural and functional points would significantly enhance the manuscript's quality.

**Reviewer #3 (Remarks to the Author):**

Brolih et al describe a characterization of CDIN1-Codanin-1 complex, which is a novel nuclease. Mutations in the two proteins lead to a rare genetic anemia CDA-I. In the first part of this work the authors confirmed that CDIN1 and Codanin-1 interact using immunoprecipitation from erythroid cells. Next, they prepared AlphaFold models of the two proteins and their complex. Stability of these models was verified using Molecular Dynamics. Interaction of the two proteins was further investigated using biophysical methods. Recombinant stabilized version of CDIN1 with three point mutations and C-terminal region of Codanin-1 (residues 866-1227) were used. The authors then demonstrated a weak RNase activity of this complex and generated AlphaFold models of complexes between RNA/DNA and CDIN1 or CDIN1-Codanin-1. The latter showed

that Codanin-1 may participate in the stabilization of nucleic acid. Indeed, in vitro the complex of the two proteins had higher RNase activity. Activity assays with numerous nucleic acid substrates led authors to conclude that CDIN1 is a 3'-5' RNA exonuclease. In the last part two pathogenic mutations in CDIN1 were studied which showed that they affect the nuclease activity.

Overall, this study is technically sound and concerns an interesting protein complex with relevance to human disease. However, in the opinion of this referee this manuscript contributes limited new information to our understanding of the activities, mechanism of action and role of this complex.

Many conclusions of the paper are based on computational methods – AlphaFold predictions coupled with MD simulations. However, the paper does not describe experimental validation of the computational structural models. The most desired validation would include structural studies or mass spectrometry after chemical cross-linking. However, mutagenesis of the residues predicted to be involved in protein-protein or protein-nucleic acid contacts and checking the effect on complex stability and enzymatic activity would already provide some support for the structural models. The evolutionary conservation of the residues predicted to participate in contacts is not discussed.

We thank the reviewer for their careful reading of the text and constructive suggestions. Reviewers 1&2 also cited the need to generate mutations that interfere with the protein-protein interaction between Codanin-1 and CDIN1, as well as between the complex and nucleic acids. We agree that this is an important validation step to test our modelling data. We have therefore carried out extensive mutagenesis, coupled with purification of the variant forms of the protein, to address these points.

New interaction mutants and substrate binding mutants made based on structure predictions and quantitatively and thoroughly validated with surface plasmon resonance, analytical size exclusion and substrate-binding assays (FP). Please see New Fig 5 as well as further information added to Fig 3 (and Suppl. Fig 9 and Suppl. Fig 10) and associated text on pages 18-20.

Importantly, the structural predictions should provide information which would help understand the mechanism of the studied complex, for example the structural basis of the specificity of the nuclease for RNA. This should be readily testable using relevant protein variants with point substitutions or modified substrates. Another basic check of the correctness of their modelling, which is not shown, is the analysis of the interactions of the phosphodiester backbone (in particular scissile phosphate) of the RNA with the active site in their models. Is the trajectory of the cleaved RNA strand, its polarity and positioning similar to those observed in experimental structures of other PD(D/E)XK nucleases? Finally, the model of the complex interacting with RNA/DNA suggests that Codanin-1 provides additional contacts with the nucleic substrate. Therefore, CDIN-1 alone should have lower affinity for the nucleic acid than the complex of the two proteins which has not been tested.

We agree that these points were important to address. In regard of the nucleic acid strand trajectory, we have made substantial improvements in the new version of this manuscript. As

shown in Fig. 3B, we have now performed a comparative analysis between the apo and the dsRNA-DNA–bound models. Overall, the binding modes and key interactions remained largely consistent between the two states. Notably, the CC domain of the Codanin-$1^{866-1227}$ region exhibits conformational flexibility, adopting a more open configuration in the bound model to accommodate both RNA and DNA substrates. This conformational adaptability helps reconcile the observed binding with the reported oligomeric state. We have now added these details and clarifications to the related main text of the manuscript for improved clarity and completeness.

The reviewer makes an insightful prediction regarding the contribution of Codanin-1 to nucleic acid interaction which were able to test by fluorescence polarization (FP) new Fig. 3C. FP analysis also revealed that the nucleic acid-binding affinity of complexes was affected most by Codanin-1 mutations, consistent with this prediction (new Fig 5C, new Suppl. Fig. 10).

It is important to note that the nuclease activity observed in the in vitro assays is rather weak. 25-fold molar excess of the nuclease and 6 to 12.5-fold molar excess of the complex over substrate was required to observe nuclease activity. This is not unprecedented that in vitro nuclease assays require excess of the enzyme. However, how can the Authors ensure that the observed activity is physiological given that such high nuclease concentration needs to be used in the vitro assay? On a related note Supplementary Fig 8E appears to show that CDIN1-Codanin-1 has much more robust activity on an R-loop-like substrate. This is not explored further or discussed.

As the reviewer rightly mentions, a challenge for the nuclease field is that reactions often require an excess of enzyme – in fact this is typical for mammalian nuclease enzymes *in vitro*. This could be for several reasons, but it is tempting to speculate that expression of super-active and highly processive nucleases could be dangerous to cells, and therefore such activity is primarily spatially regulated and directed to substrates in a highly controlled manner preventing deleterious off-target digestion.

It is of interest that this complex with process R-loop-type structures. That said, the presence of the R-loop itself is not specifically required for this activity (cleavage occurs efficiently without the actual "loop" part on a related RNA-DNA hybrid, Suppl. Fig. 8E). We have therefore been cautious not to overinterpret this activity, while agreeing that is interesting.

Authors also used in silico approaches to study the interactions of Mg2+ cofactor with the active site. These analyses showed binding of one metal ion. They are, however, probably not relevant to the actual catalytic mechanism, because two metal ions are involved in nucleic acid hydrolysis and they would be properly organized at the active site in the presence of the appropriately bound nucleic acid.

Thank you for this comment. We agree that nucleic acid hydrolysis commonly proceeds through a two-metal-ion mechanism, and the proper catalytic geometry typically requires precise organization of both $Mg^{2+}$ ions around the scissile phosphate.

In our *in silico* studies, we explored both one-metal and two-metal configurations in the presence of substrate as well. As shown in new Suppl. Fig14, the active site is capable of supporting either a single bound $Mg^{2+}$ (a) or a transiently populated two-metal arrangement (b). In the two-metal simulations, one $Mg^{2+}$ ion consistently adopts a stable coordination pattern with catalytic

residues, while the second ion is more weakly stabilized and samples a broader range of positions. This behavior is consistent with the idea that the second $Mg^{2+}$ may only become fully ordered under specific catalytic conditions or along the reaction coordinate. Instead, we note that both the one-metal and two-metal configurations observed in our models are compatible with nuclease activity, and both have been reported in related systems. A comprehensive investigation of the catalytic pathway and the role of the two-metal geometry will be addressed in future work.

In summary, this manuscript does not provide sufficient validation of the computational results. The main finding is the demonstration of the RNase activity of CDIN1 and the modification of this activity by Codanin-1. This is however, relatively limited new information.

We hope the reviewer agrees our extensive mutagenesis, related functional studies and substrate modelling work represents a very significant improvement and provides the requested validation.

Minor points:
Line 164: the title of the second section states "in vitro interaction" of the two proteins which is misleading because this section describes in silico modelling and MD simulations.

We agree and have changed to '*in silico* modelling'.

The presentation of the data could be improved by including domain schematics of the studied proteins. For example, in Fig. 1 the model labeled Codanin-1 is in fact the C-terminal module and only after checking the Supplementary Fig. 2 it becomes apparent that this is only a part of the structure of the protein.

We agree, and have updated Figure 1 accordingly, using the nomenclature Codanin-1-$^{1866-1277}$ rather than just Codanin-1, as we are specifically referring to the C-terminal region of Codanin-1, not the full-length protein.

The Authors need to discuss whether the mutations they introduce to stabilize CDIN1 could affect nucleic acid binding, enzymatic activity and interactions with Codanin-1.

We believe this is extremely unlikely, since all the stabilisation mutants reside in non-conserved residues.

Line 336: "negatively-charged face forms an extensive contact with the RNA-DNA substrate". It is usually positively charged patches that interact with the phosphodiester backbone of nucleic acids. Is this a typo?

Thanks for spotting this, we have amended.

Reviewer #1 (Remarks to the Author):

The authors have satisfactorily responded to and addressed the queries and suggestions offered in peer review.

We thank the reviewer for their previous constructive comments and suggestions.

Reviewer #2 (Remarks to the Author):

Referee Report
I thank the authors for their revised manuscript and for their response to my previous referee report. However, several of the major concerns raised earlier have still not been adequately addressed. In some cases, the authors state in their response that changes were implemented (e.g. Figure 1 and inclusion of a domain overview), but these changes are either not evident in the manuscript or do not sufficiently resolve the issues raised. Overall, while the biochemical data are interesting and suggest that the Codanin-1–CDIN1 complex exhibits RNA exonuclease activity that is enhanced by Codanin-1, substantial clarification, correction, and more careful interpretation are still required. In addition, brackets are repeatedly used for already published or well-established results; these should generally be removed to improve clarity and tone.

Major Points
- Lack of a clear domain overview in Figure 1
There is still no meaningful domain overview of Codanin-1 in Figure 1. In particular, I cannot identify a clear diagram indicating which Codanin-1 domains are excluded in the modeling. This was explicitly requested in my previous report and remains unresolved.

We thank the reviewer for the suggestion, which is a good one, and realise perhaps we did not previously fully appreciate what was being requested. A separate colour-coded schematic diagram has now been added to Fig. 1C clearly illustrating the constructs and domains that were focused on in this study.

- Neglect of Codanin-1 / CDIN1 dimerization
At line 87, the phrase “(potentially homodimeric)” is placed in brackets, which is misleading. Cryo-EM and accompanying biochemical data by Jeong et al. clearly demonstrate that the Codanin-1–CDIN1–ASF1 complex, as well as the Codanin-1–CDIN1 complex, are dimeric. This established finding is neither properly acknowledged nor discussed in the Introduction or Results, despite its obvious relevance for the structural and functional interpretation of the data presented here.

The finding was published around the time of initial submission, hence its omission from our original manuscript. In the paper, only the structure of Codanin-1 (residues 1-833) and Asf1 are observed. However, the Cryo-EM structure of Codanin-$1^{866-1227}$ and CDIN1 are not seen in the reported structure so there is no experimental proof of dimerization of the relevant complex. Please also see our other comments about the Cryo-EM data below. Because the CDIN1-Codanin-$1^{866-1227}$ complex elutes at a volume consistent with a homodimer (Suppl. Fig. 4D), we agree this is a possibility and indeed acknowledge this in the text related to this panel : ‘the two protein co-

migrate on size exclusion chromatography with an elution volume on an analytical column with a mass corresponding to 130 kDa, this is consistent with a theoretical mass of a dimer-dimer complex (aabb complex)'. Taken together we agree that the complex is likely to be dimeric, but this is not definitively proven. We therefore have changed 'potentially homodimeric' to 'likely homodimeric'.

- Figure 1B: labeling and Western blot interpretation
In Figure 1B, the Codanin-1 label appears to be positioned at an incorrect height. In addition, a second band is visible in the Codanin-1 Western blot. Please clarify what this band represents.

Thank you for your comment. The band placement has now been amended. The second band present on the Codanin-1 western blot is present in the antibody data sheet across several cell lines (Bethyl A304-951A). It is a non-specific band that appears in certain cell-lines. This has previously been described in the literature (Ask et al. 2012) where the authors performed siRNA experiments to confirm the top band represents Codanin-1. We have annotated the panel to indicate that the second band is a non-specific band.

- SPR (Biacore) data interpretation (Figure 1F). In the Biacore experiment shown in Figure 1F, the association curves flatten at high protein concentrations. This behavior suggests that the interaction may be limited by surface saturation or mass transport rather than intrinsic binding kinetics. While the reported KD value is likely within the correct range, applying a global kinetic fit to the SPR data—excluding concentrations affected by surface saturation—would further validate the binding analysis.

Thanks – we accept this point, as on closer examination the data fitted in panel Fig. 1F do show signs of surface saturation. We did attempt to fit the data using a kinetic fit, but as the reviewer suggests a satisfactory fit cannot be obtained using the entire concentration series, suggesting responses are affected by mass transport. Following the reviewer's suggestion, we applied a global kinetic fit to the data excluding the three highest concentrations. This data was possible to fit approximately and gives kinetic parameters more in line with the data we subsequently measured using single cycle kinetics in Fig. 5B (Kd= 26.8 nM). We have updated the plot in Fig. 1F to remove the highest 3 concentrations from the dose response curve which now also fits slightly better with a Kd ~50 nM, and added the following to the manuscript text:

"We further characterised this interaction by surface plasmon resonance (SPR) (Figure 1F). The data were fitted using a steady state affinity model showed a double-digit nM range interaction affinity ($K_d$=49.3 ± 17 nM) although signs of mass transport limitation were evident in the data especially at higher protein concentrations."

We have also altered the discussion in page 20 that addressed the discrepancy between the single cycle and multicycle SPR affinities since they are now more consistent: "which is in accordance with the previously-measured multi-cycle analysis in Fig. 1F".

- Unclear sentence at page 3, line 96

The sentence starting at line 96 on page 3 remains unclear and poorly written and should be rewritten for clarity.

We agree, the sentence had become corrupted at some point and has been rewritten.

- Misleading surface representations
Surface representations that give the impression of experimentally determined cryo-EM structures are still present in the supplementary figures. These representations are misleading and should be revised or clearly labeled as models.

Thank you for your suggestion. We have now changed the rendering in all supplementary figures (as we did previously for the main figures) and now also added clear labels under each figure stating “AlphaFold model”.

- Critical residue mislabeling (K216 vs R216)
In Supplementary Figures 1, 2, 4, 6, and Supplementary Figure 14, residue 216 is labeled as R216, whereas the correct residue is Lysine (K216), as shown in the sequence and in the structural representations. Since this residue is part of the catalytic triad, this error is substantial and must be corrected in all figures. Notably, in the sequence alignment in Supplementary Figure 4, the residue is correctly labeled as K216.

This has now been fixed in all cases.

- Missing consideration of the dimeric Codanin-1–CDIN1 complex in AF3 modeling
I ran AlphaFold3 on the dimeric Codanin-1–CDIN1 complex, and the prediction suggests that the dimer interface may interfere with the CDIN1 binding site proposed in this manuscript when considering the full-length protein. At minimum, the authors should comment on this issue. Ideally, they should show an AF3 prediction of the Codanin-1 dimer or overlay the dimeric cryo-EM structure with their complex prediction (e.g. in Supplementary Figure 2 and page 7, line 228). While I understand that the interacting C-terminus of Codanin-1 may be flexible and potentially unaffected by dimerization, it would still be important to show where the N-terminal ~865 residues would be located and how a full-length dimeric complex could be arranged. Furthermore, the authors demonstrate that Codanin-1–CDIN1 forms a dimer–dimer complex; it is therefore unclear why this configuration was not used for the AF3 prediction, which is technically feasible.

We thank the reviewer for the comment and would like to offer following clarifications:

We also ran AF3 using a dimeric (2:2) CDIN1-Codanin-$1^{866\text{-}1227}$ (C-terminus) complex, this dimer model is indeed somewhat different from our predicted monomer in that the Codanin-1 predicted dimer interface is formed by the CC domain which no longer contacts CDIN1 in the same manner (image below left). We note that in this prediction the remaining interface between CDIN1 and eight α-helix bundle of Codanin-1 is the same as our predicted monomer (1:1) complex. The off-diagonal elements of the PAE matrix for this prediction indicated low or medium confidence for relative positioning of Codanin-1 dimers (regions marked A, B and C on the PAE

matrix below right), with arguably only the CC domain (marked A) having significant confidence.

AF of dimeric CDIN1-Codanin-1 complex:

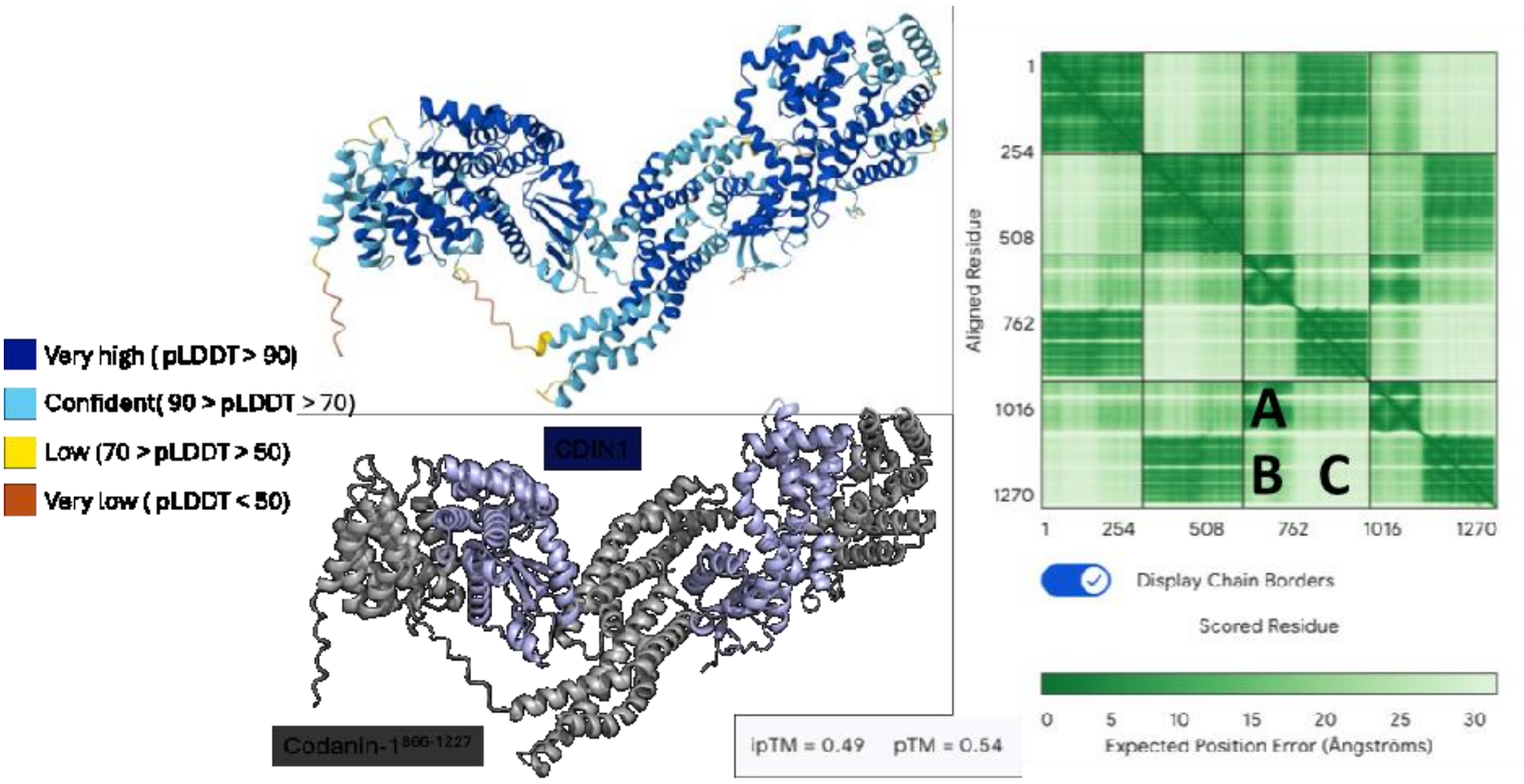


AF3 predictions using either a full length Codanin-1-ASF1 dimer (as for the cryo-EM structures) or a full length CDIN1-Codanin-1 (2:2) complex reveal a dimer interface predominantly centred around the Codanin-1 N-terminus which agrees with the recent cryo-EM structures and has high confidence for the relative positioning of the two Codanin-1 protomers across this interface (shown below and labelled A'). Again, a secondary interface is predicted between the CC domains with medium confidence (marked B' below) but importantly this region was not observed as ordered density in the experimental cryo-EM structure and actually is overlapped by the secondary ASF1 binding site in the 9CVC structure (see overlay below).

AF model of dimeric full length Codanin-1:

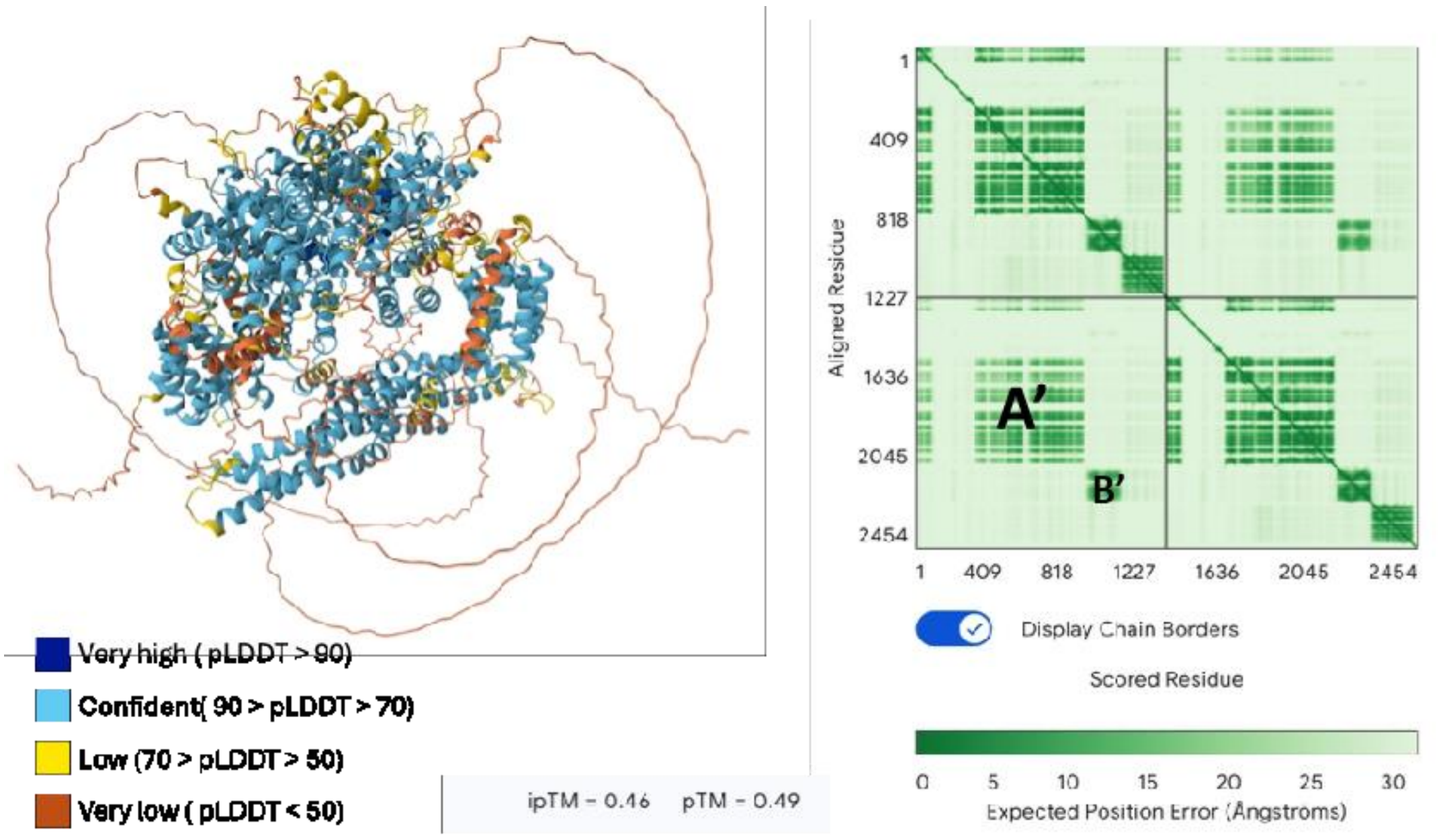


Overlay of AF3 full length Codanin-1 ASF-1 complex dimer and 9CVC/9IMZ Cryo-EM structures:

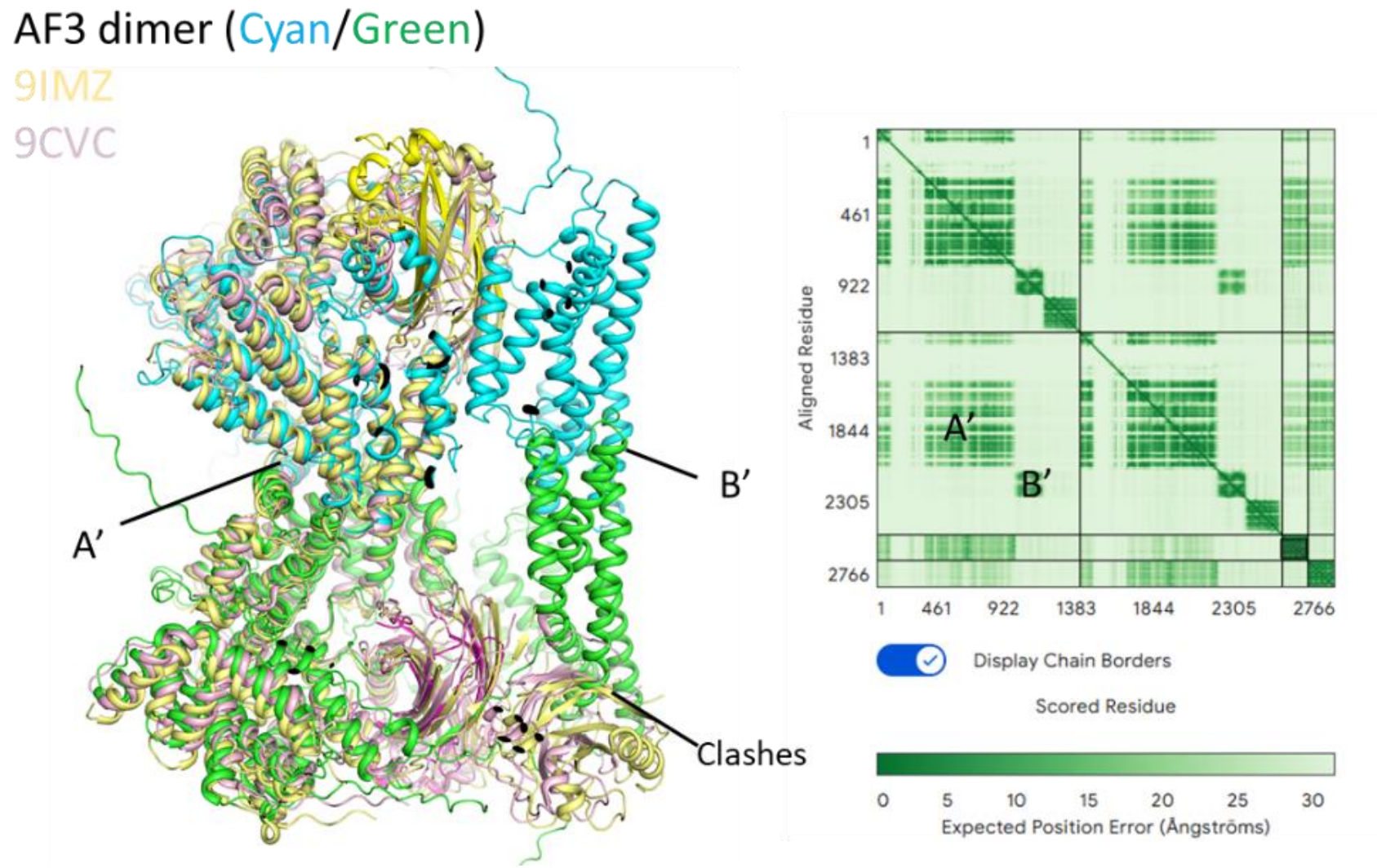


Taken together, AF3 clearly predicts a dimer interface similar to that identified by cryo-EM. The predictions also indicate that that N-terminal and C-terminal regions of Codanin-1 are independent and dynamic; features also seen in the recent cryo-EM structures. There is a portion of the dimer interface that is predicted by AF3 to feature an association of the CC domain but this part of the interface has not been validated experimentally.

The cryo-EM structures published in Jeong et al. and Sedor et al., both used full length Codanin-1 although, in both cases, ordered density only covers residues 1-833. The models lack any density from the C-terminal Codanin-1$^{866-1227}$ (or any CDIN1 density) which is disordered and thus we have no reliable way of arranging the C-terminal Codanin-1 on to this dimer model. We believe that further conclusions on full-length CDIN1-Codanin-1 complex requires further structural experimental studies, although we accept the point of the reviewer that these issues should at

least be discussed. We have added the following to the manuscript, page 23 (discussion section):

"It is not understood how the C-terminal regions of Codanin-1 used in our study may contribute to these dimer/multimer formations: AF3 modelling suggests a possible additional contribution provided by the CC domain which is distinct from the CC domain interaction with CDIN1 and RNA described here (Suppl. Fig. 16A). However, this part of the interface lacks experimental support and overlaps with the ASF1-A2 site in the cryo-EM structures, suggesting careful interpretation is needed. As there is no overlap between the resolved cryo-EM structures and our AF/MD models, we tentatively suggest a model where both functional ends of Codanin-1 (namely the ASF1-Codanin-1 and the CDIN1-Codanin-1 end) are likely linked by the large flexible regions identified in our AF models."

- Excessively long subtitles
The very long subtitles (e.g. "Cloning and site directed mutagenesis of CDIN1S … Codanin-1 complex") should be shortened. For example, this subtitle could simply be changed to:
"Cloning and site-directed mutagenesis of CDIN1 and Codanin-1."

These have been fixed, and we agree that those in the Methods were cumbersome in the previous draft.

- Overstatement of mutational analysis
In the Results/Discussion section (around line 650), the conclusions drawn from the mutational analysis are overstated. While some mutations support the predicted interface, others do not show an effect. In addition, several patient mutations not located in the predicted interface have a stronger impact on nuclease activity. A more careful formulation would be appropriate, for example:
"The mutational analysis partially supports the predicted model. Notably, several patient-derived mutations outside the proposed interface show a more pronounced effect on nuclease activity."

Thank you for your comment. In order to avoid any overstatements, we have amended our conclusions in the results/discussion to offer a more nuanced interpretation of our mutagenesis. The relevant sections now state:

p20 "Taken together, these results are consistent with our predicted interface derived from the AF/MD models. While substitutions within the proposed interface reduced complex formation, the magnitude of effects varied, suggesting that individual residues contribute differently to complex stability. Notably, no single mutation abolished the interaction, indicating that cooperative contributions from multiple residues likely stabilize the complex."

p21 "This data is consistent with the predictions from our structural modelling"

p22" Looking at our structural models, both residues are located away from the predicted interaction domain of CDIN1 and Codanin-1."

p24 “We also identify key residues involved in the binding interface between CDIN1 and Codanin-1 and provide functional support for our AF/MD approach using site-directed mutagenesis on these residues. Collectively, the data are consistent with the proposed interface, although the variable impact of individual mutations suggests residues contribute differentially to complex stability. Disruption of multiple residues may be required to more substantially destabilize the complex.”

P26 “Both patient mutations are not located in the predicted interface between CDIN1 and Codanin-1.”

Minor Points:

Line 593: “Cadanin-1-CDIN1” should be corrected to “Codanin-1–CDIN1.”

Corrected

Line 745: In the phrase “and Codanin-1 and demonstrate for that the,” please remove “for.”

Corrected

Line 756: In the sentence referring to previous studies, remove “neither study.”

Corrected by removing ‘in both cases’ as this was redundant with ‘neither study’.

Line 800: Please clarify the question mark in “The footprint of the CC domain may offer an explanation as to why a strong preference?”

We could not see this question mark in our Word draft, so are unable to amend. Might be a pdf conversion artefact?

Line 818: Please clarify whether the statement refers to something that is likely lethal or has been experimentally shown to be lethal.

No null mutations have been reported in patients so far and DepMap indicates CDIN1 is strongly selective for survival across many cell lines, as cited on the same page of the Discussion below. We have changed ‘likely’ to ‘could’ as this point is not definitively resolved.

Conclusion

In conclusion, while the biochemical data are intriguing and generally support a model in which the Codanin-1–CDIN1 complex exhibits RNA exonuclease activity that is enhanced by Codanin-1, key structural and mechanistic aspects remain insufficiently addressed. In particular, the absence of a dimer-aware AF3 model (with or without nucleic acid) or at least a critical discussion thereof, as well as the issues with SPR and ITC data interpretation, weaken the overall conclusions. The Discussion section requires revision to more accurately reflect the limitations of the current analyses.

I therefore still recommend revision addressing the points above.

Reviewer #3 (Remarks to the Author):

The authors appropriately addressed my comments and introduced new data, thereby improving the manuscript.

I have one additional comment: the fits in Figure 5C seem unusual. The experimental points are far from the fitted binding curve, and it is not clear whether the values in the table confirm the importance of the selected residues. Plots for each variant should be shown separately so that readers can evaluate the quality of the fit.

Thank you for flagging this up, the reviewer is correct. There was a mistake in the colour scheme where lines and points didn't match up which has now been amended. For the curve fits, we decided to fit the collection of curves using a 4-parameter logistic fit with shared parameters for the top and bottom for all data sets. The top and bottom represent the FP values for fully bound and unbound DNA complexes and are not expected to change amongst the mutants. This fitting is more robust and gives more reasonable values for the two mutants R872A and K879A that do not approach saturation and are otherwise extrapolated to very wide values. We have also added plots for each variant in Suppl. Fig. 10B for further clarification.
